# The deubiquitinase USP44 promotes Treg function during inflammation by preventing FOXP3 degradation

**DOI:** 10.15252/embr.202050308

**Published:** 2020-07-09

**Authors:** Jing Yang, Ping Wei, Joseph Barbi, Qianru Huang, Evan Yang, Yakun Bai, Jia Nie, Yanhang Gao, Jinhui Tao, Ying Lu, Chichu Xie, Xiaoxia Hou, Jiazi Ren, Xingmei Wu, Jian Meng, Ying Zhang, Juan Fu, Wei Kou, Yayi Gao, Zuojia Chen, Rui Liang, Andy Tsun, Dan Li, Wenzhi Guo, Shuijun Zhang, Song‐Guo Zheng, Junqi Niu, Paul Galardy, Xuemei Tong, Guochao Shi, Huabin Li, Fan Pan, Bin Li

**Affiliations:** ^1^ Shanghai Institute of Immunology and Department of Immunology and Microbiology Shanghai Jiao Tong University School of Medicine Shanghai Jiao Tong University Shanghai China; ^2^ Department of Otolaryngology Ministry of Education Key Laboratory of Child Development and Disorders National Clinical Research Center for Child Health and Disorders (Chongqing) China International Science and Technology Cooperation base of Child Development and Critical Disorders Children's Hospital of Chongqing Medical University Chongqing China; ^3^ Key Laboratory of Molecular Virology & Immunology CAS Center for Excellence in Molecular Cell Science Unit of Molecular Immunology Institut Pasteur of Shanghai Shanghai Institutes for Biological Sciences Chinese Academy of Sciences Shanghai China; ^4^ Department of Immunology Roswell Park Comprehensive Cancer Center Buffalo NY USA; ^5^ Immunology and Hematopoiesis Division Department of Oncology Sidney Kimmel Comprehensive Cancer Center Johns Hopkins University School of Medicine Baltimore MD USA; ^6^ Henan Key Laboratory of Digestive Organ Transplantation Department of Hepatobiliary and Pancreatic Surgery The First Affiliated Hospital of Zhengzhou University Henan China; ^7^ Department of Hepatology First Hospital Jilin University Changchun, Jilin China; ^8^ Shanghai Key Laboratory for Tumor Microenvironment and Inflammation Department of Biochemistry and Molecular Cell Biology Shanghai Jiao Tong University School of Medicine Shanghai China; ^9^ Division of Rheumatology Department of Medicine Penn State Hershey College of Medicine Hershey PA USA; ^10^ Department of Pulmonary and Critical Care Medicine Institute of Respiratory Diseases Ruijin Hospital Shanghai Jiao Tong University School of Medicine Shanghai China; ^11^ ENT Department, Affiliated Eye and ENT Hospital Fudan University Shanghai China; ^12^ Department of Pediatric and Adolescent Medicine Mayo Clinic Rochester MN USA; ^13^ Institutes of Advanced Technology Chinese Academy of Science Shenzhen China

**Keywords:** deubiquitinase, FOXP3, induced regulatory T cells, tumor immunity, USP44, Immunology, Post-translational Modifications, Proteolysis & Proteomics, Signal Transduction

## Abstract

The transcription factor forkhead box P3 (FOXP3) is essential for the development of regulatory T cells (Tregs) and their function in immune homeostasis. Previous studies have shown that in natural Tregs (nTregs), FOXP3 can be regulated by polyubiquitination and deubiquitination. However, the molecular players active in this pathway, especially those modulating FOXP3 by deubiquitination in the distinct induced Treg (iTreg) lineage, remain unclear. Here, we identify the ubiquitin‐specific peptidase 44 (USP44) as a novel deubiquitinase for FOXP3. USP44 interacts with and stabilizes FOXP3 by removing K48‐linked ubiquitin modifications. Notably, TGF‐β induces USP44 expression during iTreg differentiation. USP44 co‐operates with USP7 to stabilize and deubiquitinate FOXP3. Tregs genetically lacking USP44 are less effective than their wild‐type counterparts, both *in vitro* and in multiple *in vivo* models of inflammatory disease and cancer. These findings suggest that USP44 plays an important role in the post‐translational regulation of Treg function and is thus a potential therapeutic target for tolerance‐breaking anti‐cancer immunotherapy.

## Graphical abstract

The ubiquitin‐specific peptidase 44 (USP44) interacts with FOXP3 and stabilizes the transcription factor by removing K48‐linked ubiquitin modifications. USP44 is essential for the establishment of fully functional regulatory T cells.

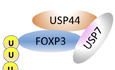

## Introduction

Regulatory T cells (Tregs) expressing the transcription factor forkhead box P3 (FOXP3) are essential for maintaining self‐tolerance and immune homeostasis (Sakaguchi *et al*, 2008). In humans, mutation of the *FOXP3* gene results in immunodysregulation polyendocrinopathy and enteropathy, X‐linked syndrome (IPEX) (Bennett *et al*, 2001a,b). Concomitantly, in mice, a lack of functional FOXP3 is associated with a similar, widespread loss of immune control that underlies the “Scurfy” phenotype (Brunkow *et al*, 2001). Historically, Treg cells have been classified into two different subtypes, determined by the tissue where they develop. Thymus‐derived or “natural” Treg (tTreg or nTreg) constitute the majority of circulating FOXP3^+^ Tregs and are crucial for preventing autoimmunity (Miyara & Sakaguchi, 2007; Schmidt *et al*, 2012). Tregs induced in peripheral tissues (pTregs) or *ex vivo* (iTregs) arise from naïve T cells that acquire FOXP3 expression and suppressive function. This occurs through the activation of the TGF‐β and IL‐2 signaling pathways (Josefowicz *et al*, 2012a). Other compounds such as all‐trans retinoic acid can further potentiate the differentiation of FOXP3^+^ Treg cells while inhibiting the generation of related but functionally opposite Th17 cells from naïve precursors (Samanta *et al*, 2008; Liu *et al*, 2015). Cells of this pTreg population have been characterized as specialized enforcers of immune homeostasis at mucosal barrier sites (Josefowicz *et al*, 2012b).

While the unique contributions of each of these subsets to immune control and the characterization of surface markers able to differentiate between them (e.g., Helios, Nrp1) are subjects of ongoing investigations (Thornton *et al*, 2010; Yadav *et al*, 2012; Zabransky *et al*, 2012; Szurek *et al*, 2015), FOXP3^+^ Treg cells generally suppress immune responses by multiple mechanisms. These include, but are not limited to, cytokine (IL‐2) deprivation, CTLA‐4‐mediated subversion of antigen‐presenting cell function, and inhibition of effector T cell proliferation by release of anti‐inflammatory cytokines (such as IL‐35 and IL‐10) (Miyara & Sakaguchi, 2007; Schmidt *et al*, 2012). These suppressive mechanisms are underwritten by a distinct Treg signature gene expression profile that is established and maintained in large part by FOXP3 (Fontenot *et al*, 2003; Fu *et al*, 2012).

As FOXP3 is centrally important for Treg function, it is essential to understand the factors and processes responsible for modulating its expression and function. Recent studies have shown that in addition to pathways regulating transcription of the *FOXP3* gene, the expression of this key regulator also depends on complex and multilayered mechanisms operating at the post‐transcriptional and post‐translational levels as well. Recently, several mechanisms of protein‐level regulation of FOXP3 expression and function have also been brought to light. For instance, FOXP3 interacts physically or functionally with a number of co‐regulatory factors, including itself, other transcription factors, and epigenetic modifier enzymes—all participants in the so‐called “FOXP3 Interactome” (Li *et al*, 2007b; Hori, 2012). These include nuclear factor of activated T cells (NFAT), acute myeloid leukemia‐1/runt‐related transcription factor (AML1/RUNX1), Eos, and IRF4 (Wu *et al*, 2006; Ono *et al*, 2007; Pan *et al*, 2009; Zheng *et al*, 2009).

Post‐translational modifications are essential for the expression, localization, stability, and function of many target proteins. These modifications can be made through the processes of acetylation, methylation, phosphorylation, poly(ADP‐ribosyl)ation ubiquitination, and sumoylation. Recently, it was clearly shown that the activity and stability of the FOXP3 protein pool are heavily influenced by such modifications. Specifically, acetylation of FOXP3 can stabilize the transcription factor and enhance its activity (Li *et al*, 2007a; Tao *et al*, 2007; van Loosdregt *et al*, 2010), while phosphorylation can have both positive and negative effects on FOXP3 activity and Treg function, depending on the specific residues targeted (Morawski *et al*, 2013; Nie *et al*, 2013; Li *et al*, 2014). PARP‐1‐mediated poly(ADP‐ribosyl)ation of FOXP3 impairs Treg function and suggests that PARP‐1 inhibitor could be potential drug for autoimmune diseases (Luo *et al*, 2015). Our previous findings uncovered STUB1 as an E3 ligase capable of targeting FOXP3. The up‐regulation of STUB1 in Treg cells upon exposure to LPS, pro‐inflammatory cytokines, or heat shock was found to promote FOXP3 polyubiquitination. This process promoted the proteasomal degradation of the transcription factor, leading to impairment of Treg suppressive function (Chen *et al*, 2013).

Just as the ubiquitination of FOXP3 has significant implications for Treg function and phenotypic stability, deubiquitination is a process equally important in Tregs. There are more than 100 deubiquitinases (DUBs) in humans and nearly 20 that are expressed in human T cells (Jin *et al*, 2007). A recent study found that a particular DUB called ubiquitin‐specific protease 7 (USP7) both associates with FOXP3 and deubiquitinates the transcription factor. Expression of USP7 was also found to be important for preserving cellular pools of FOXP3 and the immunosuppressive function of primary Tregs (van Loosdregt *et al*, 2013). We also identified the TCR signaling induced deubiquitinase USP21 as a crucial regulator of preventing FOXP3 protein depletion and a controller of Treg lineage stability (Li *et al*, 2016). While these discoveries marked important steps in the characterization of ubiquitin‐dependent regulation of FOXP3, the cast of molecular participants impacting the expression and activity of this key Treg transcription factor remains mostly unknown. Additionally, the relative importance of protein‐level FOXP3 regulation and individual DUBs in the biology of different FOXP3^+^ Treg subsets is equally uncertain.

Previously, we used a tandem affinity purification method to purify factors found in a complex with FOXP3 in a Jurkat cell line possessing stable expression of HA‐FOXP3 (Chen *et al*, 2013; Gao *et al*, 2015). Mass spectroscopy of the FOXP3 complex identified a novel, Treg‐relevant DUB known as ubiquitin‐specific protease 44 (USP44) among the factors physically interacting with FOXP3. USP44 is known to play important roles in the cell cycle (Stegmeier *et al*, 2007; Visconti *et al*, 2012), tumor progression, and embryonic stem cell differentiation (Fuchs *et al*, 2012; Zhang *et al*, 2012). In this study, we confirmed that USP44 indeed associates with FOXP3. Furthermore, we found that it counteracts degradative, K48‐linked ubiquitination of the transcription factor, preserving both its protein level and regulatory activity. USP44 knockdown in Tregs decreased FOXP3 expression and disrupted patterns of Treg gene expression (i.e., suppression of IL‐2 expression). We also found Tregs lacking USP44 to be functionally deficient *in vitro* and less effective suppressors of inflammation *in vivo* than wild‐type controls. Moreover, these shortcomings were linked to highly unstable FOXP3 expression in the absence of USP44. We also found transcriptional up‐regulation of USP44 in response to TGF‐β‐driven Smad3 activation, while inflammatory cues effectively down‐regulated USP44 levels. Interestingly, USP44 co‐operated with USP7 to deubiquitinate and stabilize FOXP3.

These findings identify USP44 as a potent stabilizer of the FOXP3 protein pool. They also add to our grasp of the molecular pathways acting on this important T cell lineage regulator at the post‐translational level. Importantly, USP44 presents a potential target for immunotherapeutic intervention for dysregulated immune responses such as those seen in autoimmune diseases and cancer.

## Results

### USP44 is a part of the FOXP3 complex and is preferentially expressed by Tregs among the CD4^+^ T cell subsets

FOXP3 has been reported to interact with a number of factors that include epigenetic modifying enzymes and transcriptional co‐regulators (Hori, 2012). In prior studies, we used a tandem affinity purification approach to characterize molecules capable of interacting with FOXP3 (Chen *et al*, 2013; Gao *et al*, 2015). In this approach, Jurkat T cells stably expressing a HA‐tagged FOXP3 were lysed, and anti‐HA immunoprecipitation allowed for the recovery of FOXP3 molecules with their associated factors (the FOXP3 complex). Mass spectrometry analysis of the peptides associated with FOXP3 led to the identification of several chaperones and co‐factors (Gao *et al*, 2015). In addition, a polypeptide belonging to the known sequence of the DUB called USP44 was also present at low but detectable levels in the FOXP3 complexes recovered.

**Figure 1:**
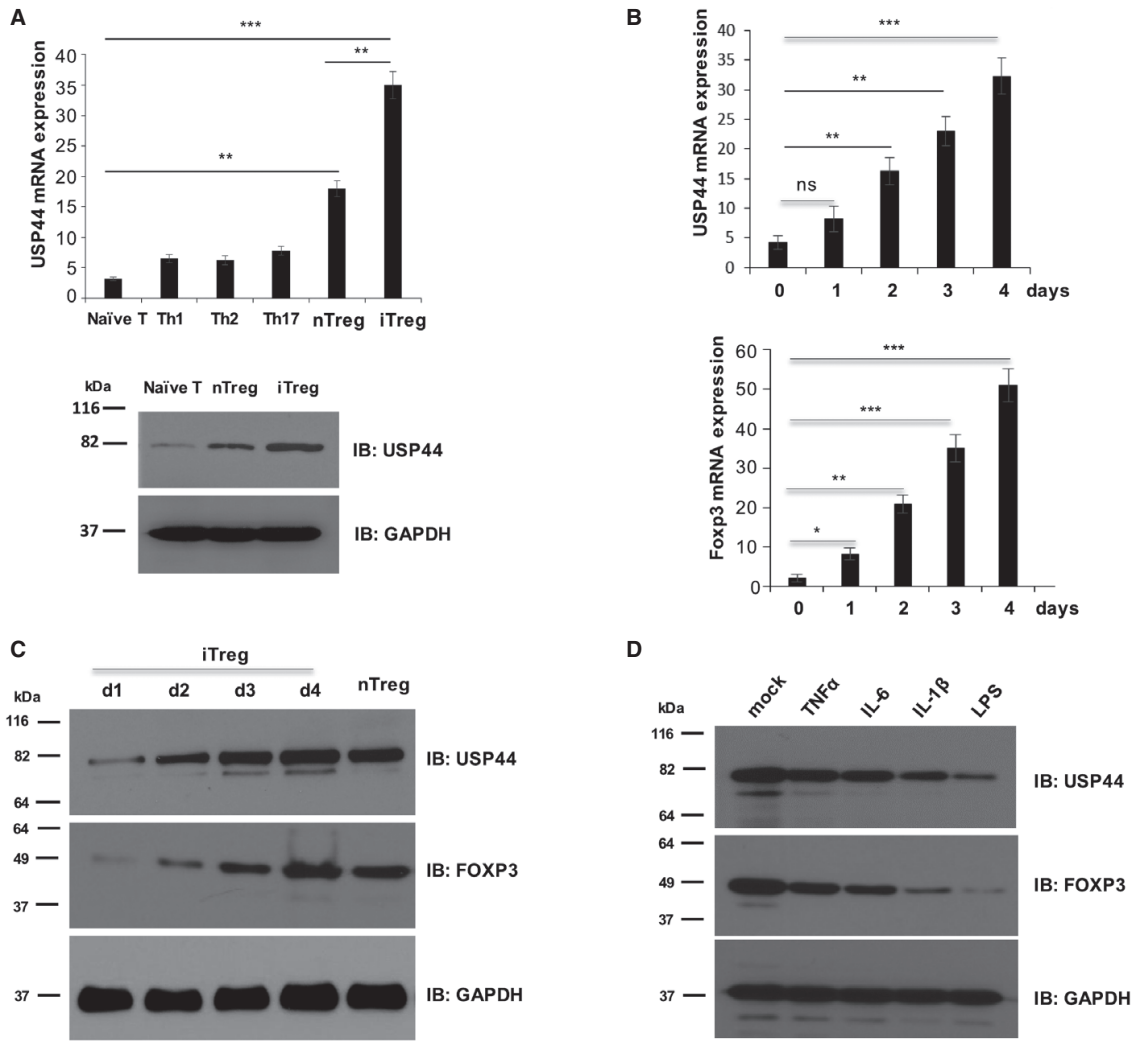
USP44 is preferentially expressed in Treg subsets (Top) USP44 transcript levels across CD4^+^ T cell populations. Suspension of the lymph node and spleen cells of 6‐ to 8‐week‐old female C57BL/6 mice (*n* = 5 experiments) was obtained, and naïve (CD62L^high^/CD25^−^) CD4^+^ T cells and nTregs (CD4^+^/CD25^high^) were obtained by FACS. The indicated effector T cell populations were generated by activating 1 × 10^6^ naïve CD4^+^ T precursors with anti‐CD3 and anti‐CD28 antibodies (1 and 4 μg/ml, respectively) for 4 days under the indicated *in vitro* skewing conditions (described in the Materials and Methods section). After harvesting RNA by TRIzol reagent and generating cDNA, the Usp44 mRNA levels expressed by the resulting Teff and iTreg, as well as freshly isolated nTreg cells, were determined by qRT–PCR. (Bottom) Suspension of lymph node and spleen cells of 6‐ to 8‐week‐old female C57BL/6 mice was obtained, and naïve (CD62L^high^CD25^−^) CD4^+^ T cells and nTregs (CD4^+^/CD25^high^) were sorted by FACS. iTregs were generated by activating 1 × 10^6^ naïve CD4^+^ T cells with anti‐CD3 and anti‐CD28 antibodies (1 and 4 μg/ml, respectively) for 4 days in the presence of IL‐2 (100 μg/ml) and TGF‐β (5 ng/ml) before the cells were harvested for SDS–PAGE.Naïve murine CD4^+^ T cells were obtained and differentiated into the iTreg lineage as in A. Expression levels of *Usp44* and *Foxp3* mRNA were assessed daily over time by qRT–PCR.Expression of USP44 and FOXP3 protein by T cells during *in vitro* differentiation was also measured. After 1–4 days of iTreg skewing, cell lysates were resolved by SDS–PAGE following by immunoblotting with the indicated antibodies.Murine iTregs generated *in vitro* as above were activated in the presence of recombinant TNFα, IL‐6, or IL‐1β (each at a 10 ng/ml concentration), or LPS (1 μg/ml) for 24 h. Treated Tregs were then harvested, and USP44 protein levels were determined as above.Data information: Panels (A) and (B) depict the mean mRNA expression normalized to the housekeeping gene GAPDH. Shown are the representative results of three biological replicates for all panels. Error bars represent the SEM. *P < 0.05, **P < 0.02, ***P < 0.002; Student's *t*‐test. Source data are available online for this figure.

**Figure EV1:**
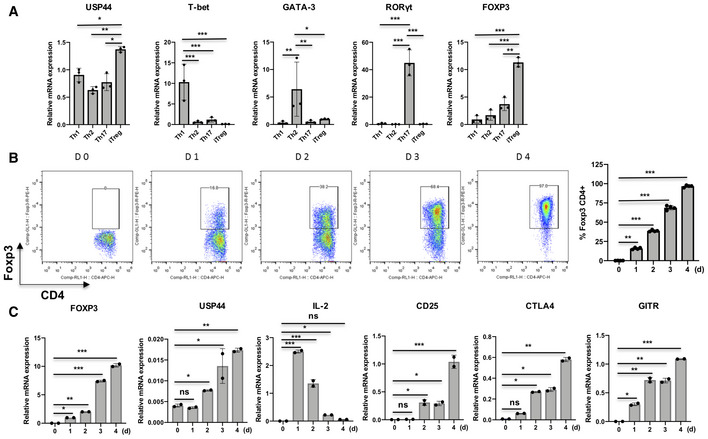
USP44 is up‐regulated in Tregs and largely tracked with the progressive elevation of Treg‐associated transcript levels Naïve CD4^+^ T cells were isolated from the peripheral blood of healthy donors (*n* = 2–3/experiment) and differentiated into the indicated T helper lineages by activation (anti‐CD3/anti‐CD28 antibodies; 1 and 4 μg/ml, respectively) in the presence of specific skewing reagents (described in the Materials and Methods section). Cells were harvested after 4 days, and mRNA was isolated for qRT–PCR analysis of USP44 message and that of key lineage‐defining transcription factors.iTregs were generated by *in vitro* skewing as above, and cells were harvested at different time points. Flow cytometry confirmed the progressive up‐regulation of FOXP3 under iTreg skewing conditions, and bar graph was displayed.qRT–PCR analysis revealed the levels of FOXP3, USP44, IL‐2, CD25, CTLA‐4, and GITR encoding transcripts in iTregs.Data information: Shown in panel (B) are representative flow plots. For panels (A) and (C), mRNA expression was normalized for housekeeping gene GAPDH. Shown are mean values of biological replicates across three independent experiments ± SEM.

While this ubiquitin‐specific protease is known to be important in the cell cycle and tumor biology (Sargin *et al*, 2007), no such role for USP44 in Tregs or immune regulation had been uncovered. Suggesting that such a role for this DUB indeed exists, USP44 mRNA and protein levels were found to be significantly elevated in both murine Tregs freshly isolated from lymphoid tissue (nTreg) and iTregs subsets compared to naive CD4^+^ T cells. iTregs similarly displayed significantly more USP44 transcript than any other *in vitro* differentiated effector CD4^+^ T cell subsets that we generated (i.e., Th1, Th2, and Th17 cells) (Fig 1A). Similar results were obtained when surveying USP44 mRNA levels in human CD4^+^ effect T cells differentiated *in vitro* and validated by measuring expression of canonical transcription factors (Fig EV1A). While nTregs expressed higher levels of USP44 mRNA and protein than their non‐Treg counterparts, *in vitro* generated iTregs consistently showed even greater expression of USP44 (Fig 1A).

Also linking USP44 expression to the process of Treg differentiation were observations that USP44 message and protein levels were progressively increased in murine naïve CD4^+^ T cells throughout their *in vitro* induction of *Foxp3* and commitment to the iTreg lineage (Figs 1B and C, and EV1B). This up‐regulation largely tracked with the progressive elevation of Treg‐associated transcript levels (including FOXP3, CD25, CTLA4, and GITR) and the silencing of IL‐2 expression (Fig EV1C), a gene expression profile typical of Tregs.

### USP44 expression is up‐regulated by TGF‐β during Treg differentiation

**Figure EV2:**
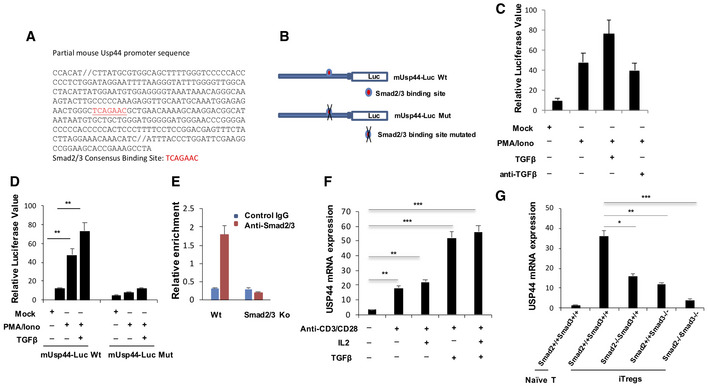
USP44 expression is induced by TGFβ under Treg differentiating conditions A, BThe USP44 promoter sequence is represented with its SMAD binding site indicated. The effect of SMAD2/3 binding site loss was found by making the mutations depicted in (B).CJurkat T cells were transfected with an USP44 promoter‐driven firefly luciferase reporter by electroporation. Cells were stimulated with PMA and ionomycin in the presence or absence of TGF‐β (2 ng/ml) or anti‐TGF‐β (10 μg/ml) for 8 h before being harvested for measurement of luciferase activity, which was normalized to Renilla luciferase activity.DJurkat T cells were transfected with an USP44 promoter‐driven firefly luciferase reporter by electroporation. Cells were stimulated with PMA and ionomycin in the presence or absence of TGF‐β (2 ng/ml) for 8 h before being harvested for measurement of luciferase activity, which was normalized to Renilla luciferase activity.EChIP analysis of SMAD occupancy of the *Usp44* promoter. Naïve T cells were isolated from C57BL/6 mice and polarized to iTreg before harvest and lysis. SMAD2 and SMAD3 factors were immunoprecipitated, and PCR was used to detect binding to the *Usp44* promoter sequence.FFACS isolated naïve T cells were exposed to the indicated stimuli during *ex vivo* culture and were harvested at different time points for mRNA isolation and measurement of USP44 expression by qRT–PCR analysis.GNaïve CD4^+^ T cells were isolated from wild‐type mice (Smad2^+/+^Smad3^+/+^) as well as mice lacking SMAD2 (Smad2^−/−^Smad3^+/+^), SMAD3 (Smad2^+/+^Smad3^−/−^), or both SMAD2 and SMAD3 (Smad2/3^−/−^), all on a C57BL/6 background (*n* = 3/group/experiment). Cells were activated under *in vitro* Treg‐inducing conditions (anti‐CD3/CD28, 1 and 4 μg/ml, respectively) in the presence of IL‐2 (100 U/ml) and TGF‐β (5 ng/ml) for 4 days. Then, RNA was extracted, and cDNA was prepared in order to assess USP44 expression levels in these cells by qRT–PCR.Data information: Panels (C–G) depict mean results from three independent experiments (biological replicates) ± SEM. *P < 0.05, **P < 0.02, ***P < 0.002; Student's *t*‐test.

Having observed preferential USP44 expression by Tregs and the robust up‐regulation of this DUB during iTregs generation (Fig 1A–C), we hypothesized that USP44 plays an important role in promoting FOXP3 expression in these cells. A critical driver of FOXP3 up‐regulation in naïve CD4^+^ T cells is the cytokine TGF‐β. Signaling downstream of the TGF‐β receptor induces the phosphorylation and activation of SMAD2/3 factors that activate expression of Treg‐associated genes, including FOXP3 (Zheng *et al*, 2010; Schlenner *et al*, 2012). This cytokine also plays an important role in the maintenance of established Treg suppressive function (Marie *et al*, 2005; Tran, 2012), as do other triggers of SMAD activity (Ni *et al*, 2018). Suspecting that USP44 may be up‐regulated in T cells by TGF‐β, we examined the promoter of the mouse/human USP44 gene using R‐vista analysis software. Indeed, one conserved SMAD binding site was found in the proximal region of both mouse and human *Usp44* promoter (Fig EV2A and B).

Based on this, we cloned the +17‐(‐314) region of the mouse *Usp44* promoter, which includes a SMAD binding site, in order to generate a *Usp44* promoter‐driven luciferase reporter construct. Introduction of this reporter into the Jurkat T cell line allowed us to confirm that TGF‐β can bring about USP44 up‐regulation. Activation with PMA and ionomycin was able to induce reporter activity to some degree in these cells. However, treatment with 2 ng/ml TGF‐β resulted in a significant increase in luciferase signal at a time point corresponding to peak SMAD2/3 activation. Interestingly, ablating TGF‐β signaling in this system with an antibody neutralizing the cytokine reduced USP44 expression in cells stimulated with PMA/Iono without exogenous TGF‐β (Fig EV2C), suggesting that induced USP44 activity may be augmented by trace amounts of TGF‐β in the serum‐complemented culture media (RPMI) used in these experiments. It is also possible that activation can render cells more receptive to TGF‐β signaling (i.e., through the up‐regulation of surface receptors). It has been reported that mitogen‐activated protein (MAP) kinase cascades triggered by T cell receptor signaling can lead to phosphorylated Smad2 (Mamura, 2000 #204). Importantly, when the SMAD binding site in the USP44 promoter of our construct was mutated, reporter activity was severely repressed, even in the presence of stimulation and TGF‐β (Fig EV2D). Corroborating the findings of these assays, a ChIP assay revealed SMAD occupancy at the promoter of the *Usp44* gene of murine iTregs (Fig EV2E), further implicating TGF‐β as driver of USP44 expression.

We also scrutinized the several stimuli that contribute to Treg‐inducing conditions for the ability to drive USP44 induction. Specifically, we assessed the impact of TCR stimulation, costimulation, IL‐2, and TGF‐β signaling separately on USP44 expression in naïve CD4^+^ T cells. While activation alone induced a modest up‐regulation of USP44 mRNA, addition of IL‐2 had little‐to‐no effect. We found that exposure to TGF‐β was chiefly responsible for inducing robust levels of USP44 in developing iTregs (Fig EV2F). These findings strongly suggest that USP44 expression in Tregs is largely driven by TGF‐β/SMAD signaling. In line with such a mechanism for USP44 induction in Tregs, and the findings obtained in our reporter system, T cells from mice genetically deficient in SMAD2 and SMAD3 fail to up‐regulate USP44 under iTreg skewing conditions, and SMAD deficiency similarly abrogated USP44 up‐regulation by iTregs (Fig EV2G).

We also explored the impact of pro‐inflammatory cues on USP44 levels. Here, isolated iTregs were activated in the presence of the toll‐like receptor agonist LPS or the inflammation‐inducing cytokines IL‐1β, IL‐6, and TNFα—stimuli previously found to undermine FOXP3 protein levels (Chen *et al*, 2013; van Loosdregt *et al*, 2013; Gao *et al*, 2015). Interestingly, exposure to either inflammatory cue markedly reduced USP44 protein levels, commensurate with FOXP3 down‐regulation (Fig 1D). These results further align USP44 expression with protein‐level regulation of FOXP3 in Tregs.

### USP44 interacts with FOXP3

**Figure 2:**
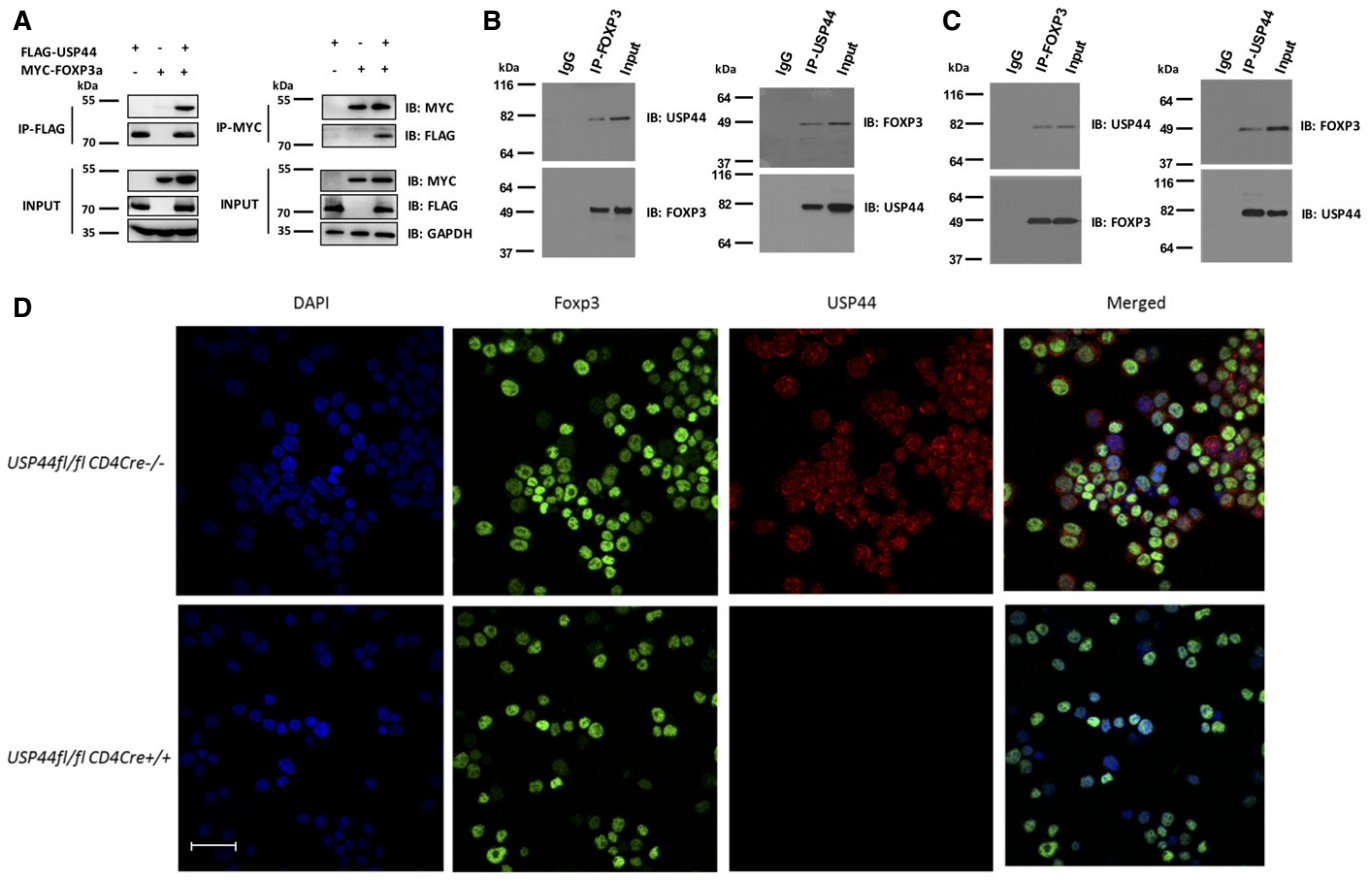
USP44 interacts with FOXP3 HEK293T cells were transfected with MYC‐FOXP3 and FLAG‐USP44 encoding expression constructs using polyethylenimine. 48 h post‐transfection, cells were harvested and lysed, and anti‐FLAG or anti‐MYC antibody‐coated beads were used to immunoprecipitate the given labeled protein along with its binding partner. Co‐immunoprecipitated proteins were subjected to SDS–PAGE followed by immunoblot analysis. Antibodies recognizing FLAG or MYC tags were used to probe for USP44 and FOXP3, respectively.Endogenous co‐IP of USP44 and FOXP3 in murine iTregs. iTregs were generated as in Fig 1 from naïve CD4^+^ T cells FACS isolated from pooled suspensions of the lymph node and spleen cells of wild‐type C57BL/6 mice (*n* = 2–3/experiment). iTregs were lysed and key proteins were immunoprecipitated using either anti‐USP44 (right panel) or anti‐FOXP3 (left panel) antibody. Proteins pulled down in this experiment were then resolved and analyzed by immunoblot using anti‐FOXP3 or anti‐USP44 antibodies.Endogenous co‐IP of USP44 and FOXP3 in murine nTregs. nTregs (CD4^+^ CD25^high^) isolated by FACS were activated by anti‐CD3 and anti‐CD28 (1 and 4 μg/ml, respectively) overnight in the presence of IL‐2 (100 U/ml). The cells were lysed and proteins were immunoprecipitated using either anti‐Foxp3 (left panel) or anti‐Usp44 (right panel). Proteins pulled down in this experiment were then resolved and identified with the indicated antibodies.Naïve murine CD4^+^ T cells were isolated by FACS from lymph node and spleen cell suspension of USP44^fl/fl^ CD4Cre^+^ mice and that of their wild‐type littermates (USP44^fl/fl^ CD4Cre‐ mice; *n* = 2–3/group/experiment). iTreg cells were generated from these mice as described for Fig 1 before incubation on a microscope slide pre‐coated with poly‐L‐lysine for 1 h. Adhered cells were then fixed by PFA for 0.5 followed by blocking with 1% BSA for 1 h and then incubated with the specified antibodies. Representative confocal microscopy images (40×) were visualized for endogenous USP44 (red) and FOXP3. DAPI was used to visualize cell nuclei (blue); scale bar 50 μm.Data information: For all panels, the results shown are representative of three biological replicates. Source data are available online for this figure.

**Figure EV3:**
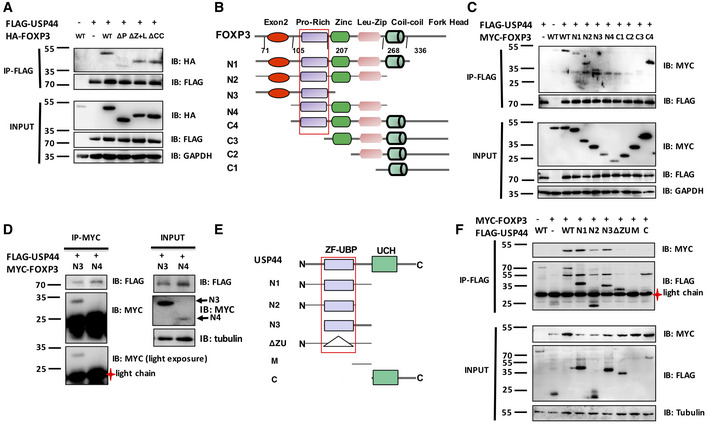
The Pro‐rich domain of FOXP3 is required for the association between USP44 and FOXP3 and the stabilization of FOXP3 ADifferent FOXP3 deletion constructs were generated as shown and were co‐transfected with or without FLAG‐USP44 into HEK293T cells. The cell lysate was immunoprecipitated by anti‐FLAG antibody, and FOXP3 levels were detected by Western blotting.B, CDifferent truncated FOXP3 constructs were generated as shown and were co‐transfected with or without FLAG‐USP44 into HEK293T cells. The cell lysate was immunoprecipitated by anti‐FLAG antibody, and FOXP3 levels were detected by immunoblotting for the MYC tag.DHEK293T cells were transfected with constructs encoding MYC‐FOXP3N3 or N4 truncations and FLAG‐USP44. These cells were lysed and pertinent factors were immunoprecipitated using anti‐MYC. Pulled‐down proteins were analyzed by immunoblot using anti‐FLAG and anti‐MYC antibodies.E, FDifferent truncated USP44 constructs were generated as shown and were transfected with or without a MYC‐FOXP3 encoding plasmid into HEK293T cells. FOXP3 protein was immunoprecipitated from cell lysate by anti‐MYC antibody, and USP44 levels were detected by immunoblotting. Shown are representative findings from at least three experiments. Source data are available online for this figure.

Prior studies implicated USP44 as a component of the FOXP3 complex. Here, the physical association between USP44 and FOXP3 was confirmed by reciprocal co‐immunoprecipitation. To this end, HEK293T cells were transfected with constructs encoding MYC‐labeled FOXP3 and FLAG‐tagged USP44. Forty‐eight hours post‐transfection, cells were harvested and lysed, and either FOXP3 or USP44 was immunoprecipitated from the cell lysates along with their interacting partners, as visualized by immunoblotting (Fig 2A). Mutagenesis mapping further characterized the interaction. Co‐expression of HA‐tagged USP44 with various Flag‐tagged FOXP3 mutants in HEK 293T cells revealed that this DUB could pull down wild‐type FOXP3 as well as FOXP3 deletion mutants lacking the zinc finger and the leucine zipper domain (ΔZ+L) or the coiled‐coil domain (ΔCC) but not one lacking the proline‐rich region (ΔP) (Fig EV3A).

Further co‐immunoprecipitation experiments tested the ability of several truncated FOXP3 mutants (Fig EV3B) to interact with USP44. Only variants containing FOXP3's proline‐rich domain could pull down USP44 (Fig EV3C and D) confirming the necessity of this domain for USP44 interaction. In a similar approach, C‐ and N‐terminal truncation mutants of USP44 were used to map the region of the DUB important for the interaction (Fig EV3E). Deletion of USP44's ZF‐UBP domain, but not the C‐terminal UCH region, was found to be critical for interaction with FOXP3. An insertion mutation of ZF‐UBP also rendered USP44 incapable of pulling down FOXP3 (Fig EV3F) supporting the conclusion that the USP44 associates with FOXP3 through ZF‐proline‐rich domain interactions.

**Figure EV4:**
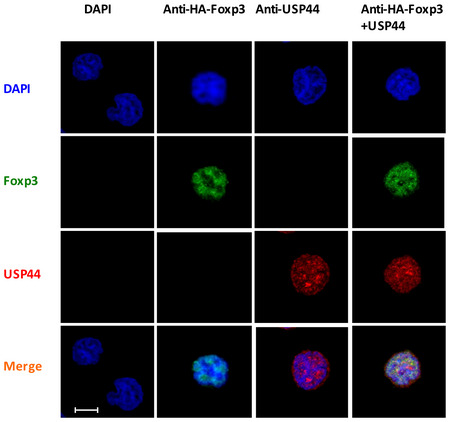
Physical interaction between USP44 and FOXP3a was visualized by immunofluorescence microscopy Jurkat‐HA‐FOXP3 cells were adhered to slides coated with poly‐L‐lysine (1:4) for 1 h and then fixed by PFA for 0.5 h. Samples were blocked for 1 h and then incubated with the indicated specific antibodies. Representative confocal microscopy images were visualized for endogenous USP44 (red) and FOXP3. DAPI was used to visualize the nuclei (blue). Shown are the findings representative of three biological replicates; scale bar 5 μm.

Endogenous co‐IP experiments further supported the notion that FOXP3 and USP44 interact. Specifically, immunoprecipitation of FOXP3 from the lysate of murine iTregs or nTregs readily pulled down USP44, and this DUB was similarly able to coimmunoprecipitate FOXP3 in reciprocal experiments (Fig 2B and C). Colocalization of these molecules was suggested by immunostaining and fluorescent microscopy in FOXP3‐expressing Jurkat T cells (Fig EV4). Confocal microscopy of primary murine Tregs confirmed the colocalization of FOXP3 and USP44 in wild‐type‐derived cells but not in cells isolated from mice lacking USP44 expression (Usp44^fl/fl^Foxp3Cre^+^; Fig 2D). Taken together, these data suggest that USP44 interacts with FOXP3 in Tregs.

### USP44 promotes FOXP3 stabilization through deubiquitination

**Figure 3:**
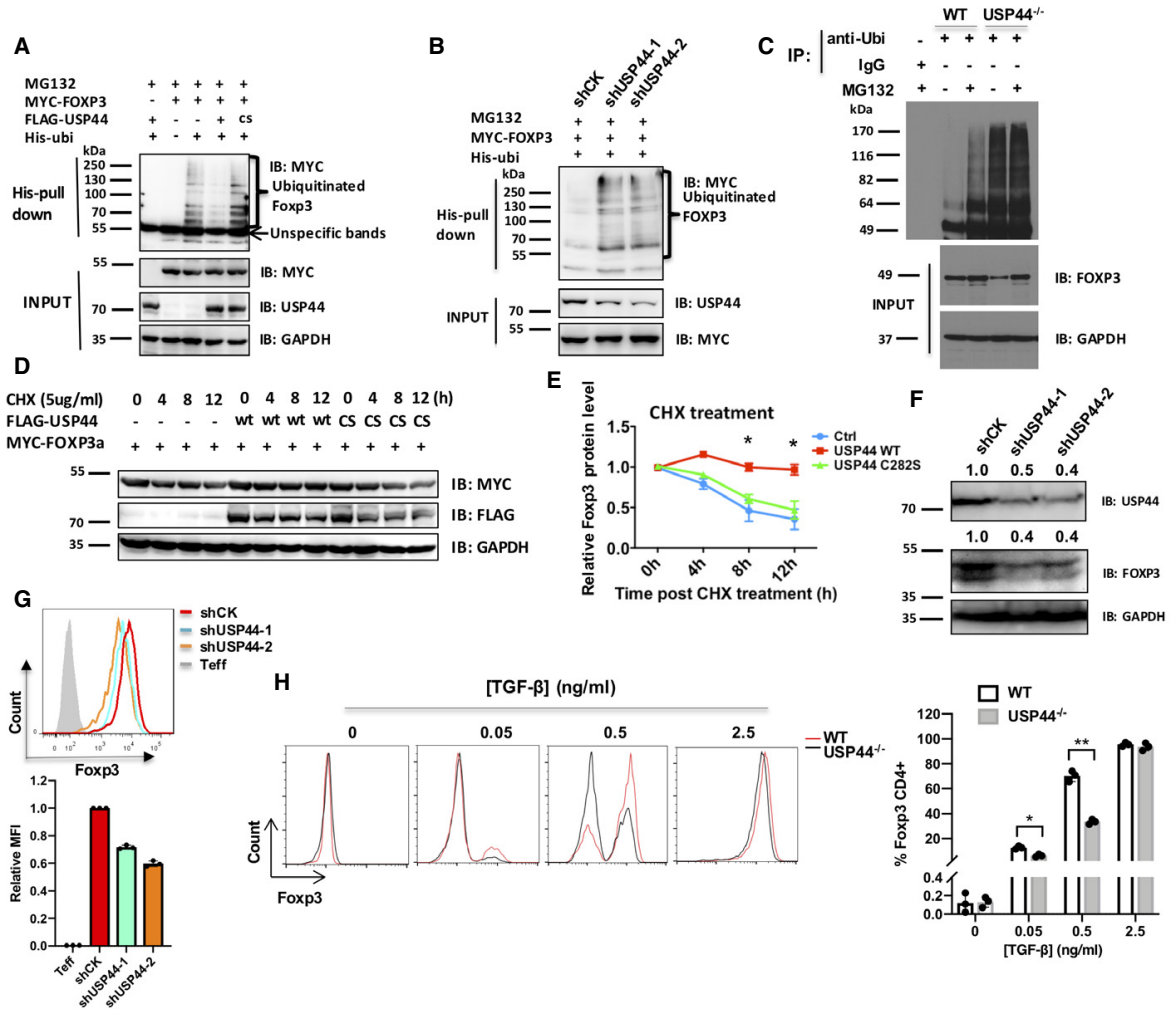
USP44 deubiquitinates and stabilizes FOXP3 AHEK293T cells were transfected with expression constructs encoding MYC‐FOXP3, His‐Ubiquitin, and either a wild‐type or catalytically inactive (C282S, “cs”) version of USP44 tagged with FLAG. Transfected cells were treated with 20 μM MG132 for 4 h and subsequently harvested for cell lysis. Pull‐down of His‐labeled proteins using Ni‐NTA beads allowed recovery of ubiquitinated FOXP3 species that were then visualized by immunoblot probing with antibodies specific for MYC.BEffect of USP44 knockdown on FOXP3 ubiquitination. Two distinct shRNA lentiviral constructs each containing a G418 resistance cassette were delivered into HEK293T cells. After selected for 7 days, cells were transfected with MYC‐FOXP3 and His‐Ubiquitin, and then treated with 20 μM MG132 for 4 h before harvest and lysis. Ubiquitinated FOXP3 proteins were visualized as in A.CLevels of polyubiquitinated FOXP3 in the presence or absence of USP44. As in Fig 2B, murine iTregs were generated from naïve CD4^+^ precursors isolated from wild type and mice globally deficient in USP44^−/−^ mice (*n* = 3/group/experiment). Ubiquitinated proteins were extracted from iTreg lysates with anti‐ubiquitin antibodies (anti‐Ubi) prior to resolution by SDS–PAGE and immunoblot analysis, probing for FOXP3.D, EHEK293T cells were transfected with plasmids encoding MYC‐FOXP3 and FLAG‐USP44 (either wild‐type USP44, “wt” or the C282S mutant). These cells were subsequently treated with cycloheximide (CHX; 5 μg/ml) for the indicated time points before harvested and cell lysis. FOXP3 levels and the relative turnover rate of this factor were determined by immunoblotting analysis with anti‐MYC antibodies.F, GUSP44 knockdown in human Tregs. Naïve CD4^+^ T cells (CD4^+^CD25^−^CD45RA^+^) were isolated from the peripheral blood of healthy donors by FACS, and iTreg were generated after 7 days of *in vitro* skewing conditions. As in Fig 3B, shRNA constructs targeting USP44 were delivered by lentivirus. Here, human iTregs received either shCK (control), or one of two different shUSP44 constructs. Endogenous FOXP3 and USP44 protein levels were visualized by immunoblot (F), and FOXP3 expression was assessed by intracellular staining followed by flow cytometry (G).HMurine naïve CD4 T cells (CD62L^+^/CD25^−^/CD4^+^) from WT and USP44^−/−^ mice (*n* = 2–3/group/experiment) were FACS purified and activated *in vitro* for 4 days by CD3/CD28 cross‐linking antibodies (1 μg and 4 μg/ml, respectively) in the presence of 100 U/ml IL‐2 and the indicated dose of TGF‐β. Down‐regulation of FOXP3 was observed in USP44^−/−^ mice by intracellular immunostaining and flow cytometry.Data information: Panels (E), (G), and (H) depict the mean results from three biological replicates ± SEM. *P < 0.05, **P < 0.02; Student's *t*‐test. Source data are available online for this figure.

**Figure EV5:**
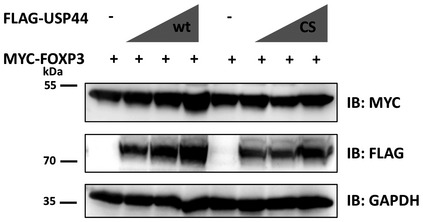
USP44 stabilizes FOXP3 in a dose‐dependent manner HEK293T cells were transfected with an expression construct encoding MYC‐FOXP3 (0.5 μg), and varying levels (0.5, 1, 1.5 μg) of vectors encoding FLAG‐tagged wild‐type USP44 (wt) or the mutant (Kralovics *et al*, 2005), after 48 h transfected cells were harvested and lysed in RIPA buffer. Immunoblots were analyzed using anti‐FLAG or anti‐MYC antibodies. Shown is a representative result of three independent experiments (biological replicates). Source data are available online for this figure.

Suspecting that USP44 targets FOXP3 for deubiquitination, we again utilized a co‐IP approach to test this notion. As expected, simultaneous co‐transfection of HEK 293T cells with constructs encoding Myc‐tagged FOXP3 and His‐marked ubiquitin resulted in distinctly ubiquitinated species of FOXP3 observable after precipitation by anti‐His (anti‐ubiquitin) beads and detection by immunoblotting for the MYC‐tagged FOXP3 protein (Fig 3A). Additional introduction of a USP44 expression vector interfered with the ubiquitination of FOXP3, suggesting that this FOXP3‐associating DUB indeed targets FOXP3. Furthermore, expression of a catalytically inactive mutant USP44 (“CS”) failed to prevent the modification of FOXP3 in this manner (Figs 3A and EV5). Similarly, shRNA‐mediated knockdown of USP44 in these transfectants also countermanded the reduction of ubiquitin‐modified FOXP3 as seen upon USP44 over‐expression or shRNA vector control treatment (Fig 3B). In these experiments, proteasome‐mediated FOXP3 loss was prevented by MG132 treatment, which permitted the recovery of polyubiquitinated FOXP3.

We further demonstrated the importance of USP44 in counteracting the process of FOXP3 polyubiquitination by examining the relative presence of this post‐translational modification in Tregs lacking USP44 expression. To this end, iTregs derived from the naïve CD4^+^ T cells of wild‐type mice or those globally deficient in USP44 expression (USP44^−/−^ mice) were analyzed for the presence of FOXP3 among ubiquitinated proteins in each sample by immunoblotting. As expected, normal Tregs contain some ubiquitinated FOXP3 species, the levels of which are enhanced by halting protein degradation with the proteasome inhibitor MG132 that stops FOXP3 protein turnover. In contrast, USP44 knockout Tregs displayed robust FOXP3 polyubiquitination at baseline and even more so upon MG132 treatment (Fig 3C). These results support a role for USP44 in the deubiquitination of FOXP3 and the stabilization of this important transcription factor's expression.

As FOXP3 is subject to ubiquitin‐mediated degradation in Tregs, and FOXP3 deubiquitination has been reported to stabilize the transcription factor (van Loosdregt *et al*, 2013), we examined the impact of forced USP44 expression on FOXP3 half‐life. Cycloheximide (CHX) treatment of HEK 293T cells expressing MYC‐labeled FOXP3 revealed decreasing FOXP3 levels by 8 h post‐treatment. Co‐expression of a wild‐type, but not a mutant, USP44‐encoding construct largely stabilized the FOXP3 protein pool in this cell line (Fig 3D and E).

In contrast, shRNA‐mediated knockdown of USP44 enhanced the disappearance of FOXP3 protein in Jurkat T cells (Appendix Fig S1A and B). This FOXP3 down‐modulation was associated with the dysregulation of some genes normally repressed by FOXP3 (Appendix Fig S1C). Importantly, introducing USP44‐silencing shRNA constructs into human primary iTregs by lentiviral transduction also reduced FOXP3 protein levels, while empty vector controls did not. This was evidenced by immunoblotting and intracellular immunostaining, respectively (Fig 3F and G). As with cell lines, shRNA knockdown of USP44 in human iTregs also derailed typical Treg gene expression patterns for *IL2*,* CTLA4*,* GITR*, and *CD25* (Appendix Fig S1D).

In line with a role for USP44 in stabilizing FOXP3 expression, we also found that T cells from mice lacking the deubiquitinase (USP44^−/−^) were less able to up‐regulate FOXP3 *in vitro* than their USP44‐competent (WT) littermates. During the early stages of iTreg differentiation, USP44^−/−^ derived T cells displayed a lower percentage of FOXP3^+^ cells (Appendix Fig S1E). Under extended iTreg skewing culture conditions, the impaired FOXP3 protein expression stemming from USP44 deficiency was most apparent when low concentrations of TGF‐β (0.5 ng/ml) were used. Interestingly, little difference in iTreg generation was seen between groups after 4 days of skewing in an abundance of the cytokine (2.5 ng/ml) (Fig 3H). Meanwhile, flow cytometry analysis of lymph node, spleen, and thymic cell suspensions revealed that levels of FOXP3^+^ Tregs were not markedly different between the lymphoid tissues of USP44^−/−^ mice and WT controls. The general CD4^+^ and CD8^+^ T cell compartments, as well as the macrophage, dendritic cell, and B cell populations of both strains, were also comparable. Mice with Treg‐specific USP44 deficiency (Usp44^fl/fl^Foxp3Cre^+^ mice) were also found to be as capable as their wild‐type littermates in maintaining these leukocyte populations and immune homeostasis (Appendix Fig S2A). Moreover, surface markers of T cell naivety and activation (i.e., CD62L and CD44, respectively), as well as pro‐inflammatory cytokine production, were all found to be similar between Treg‐specific USP44‐deficient mice and their wild type counterparts (Appendix Fig S2B). Collectively, these results suggest important roles for USP44 in the induction of iTregs under suboptimal conditions and the maintenance of the FOXP3 protein pool in developing as well as established Tregs. Yet they also seem to suggest a less prominent or perhaps a redundant role in the thymic initiation of FOXP3 expression and immune homeostasis at baseline.

### USP44 targets K48‐linked polyubiquitinated FOXP3

We then set out to determine whether or not USP44 stabilizes FOXP3 levels specifically through removal of K48‐type polyubiquitination chains—modifications known to drive proteasomal degradation of target proteins (Komander & Rape, 2012). To this end, we characterized the deubiquitination of FOXP3 by USP44 *in vitro* in the presence of wild‐type ubiquitin molecules and mutants lacking all but specific lysine residues. This approach allowed us to identify the ubiquitin linkage type targeted by USP44. Here, HEK 293T cells carrying combinations of Flag‐USP44, MYC‐FOXP3, and either wild‐type or lysine‐mutant ubiquitin constructs were treated with MG132 (to stabilize levels of ubiquitinated proteins) prior to ubiquitin (His) pull‐down and immunoblot assessment of FOXP3 species. In these experiments, ubiquitination of FOXP3 was decreased by the co‐expression of USP44 in the presence of wild‐type ubiquitin. However, a non‐lysine ubiquitin mutant (K0, wherein all lysine residues were mutated) in the triple co‐transfection scheme prevented deubiquitination. Similarly, four of the seven possible single lysine residue variants (K6, K29, K33, and K63) did not allow FOXP3 deubiquitination by USP44 co‐expression compared to wild‐type and other ubiquitin mutants. This could be explained by biochemical (e.g., conformational) changes resulting from the altered lysine content of these variants. Interestingly, for two of the single lysine residue variants (K11 and K23) ubiquitin on FOXP3 could still be removed by USP44. Notably, there was a much more pronounced reduction of K48‐linked ubiquitination of FOXP3 (almost a complete removal) (Appendix Fig S3). This suggests that USP44 mainly targets K48‐linked ubiquitin chains on FOXP3 to sustain levels of the transcription factor.

### USP44 co‐operates with USP7 to deubiquitinate FOXP3 and stabilize expression

**Figure 4:**
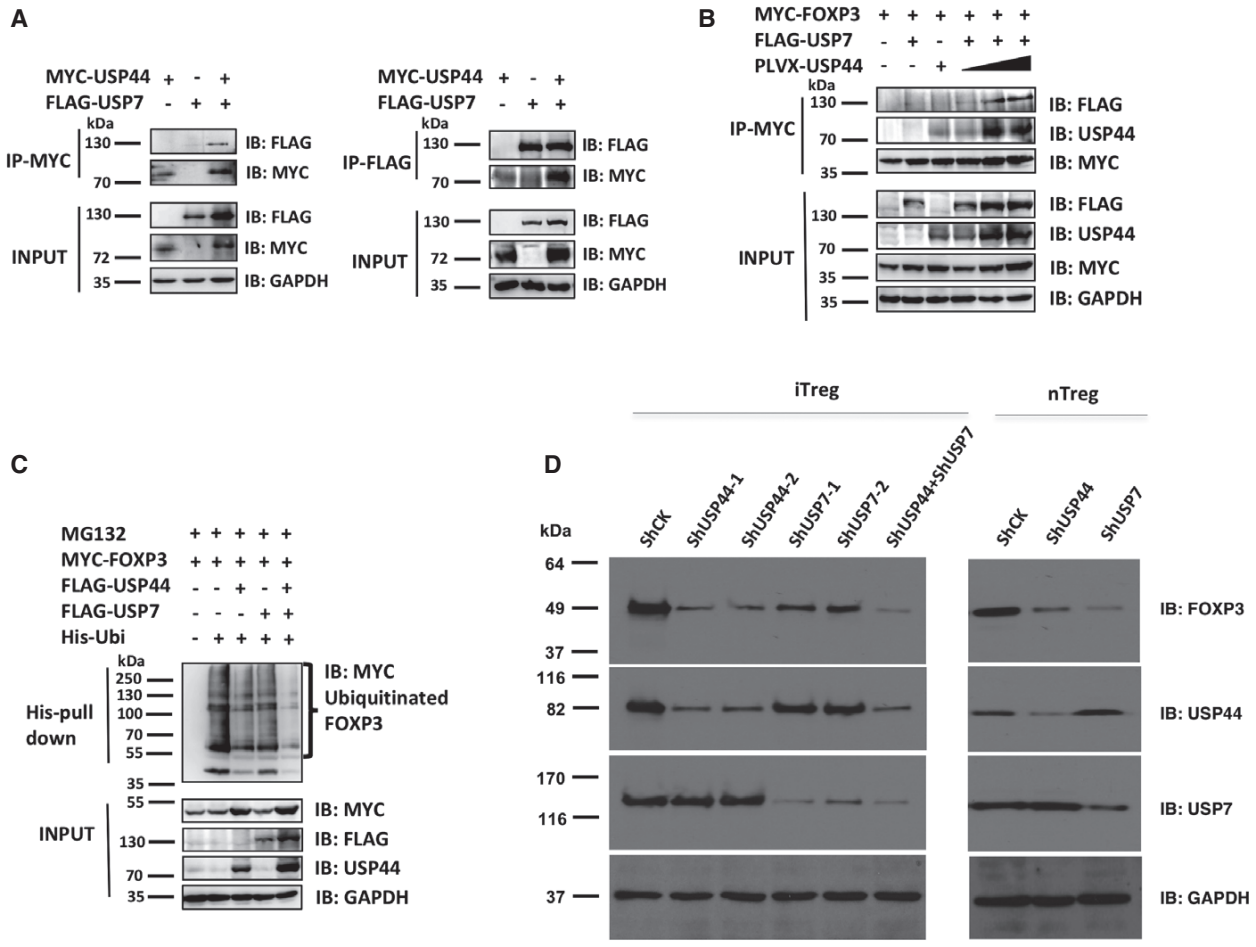
USP44 co‐operates with USP7 to deubiquitinate FOXP3 and stabilize its expression Reciprocal co‐IP of USP44 and USP7. HEK293T cells were transfected with constructs encoding MYC‐USP44 and FLAG‐USP7. These cells were lysed and pertinent factors were immunoprecipitated using either anti‐FLAG or anti‐MYC. Pulled‐down proteins were observed by immunoblot analysis using either anti‐MYC or anti‐FLAG antibodies.Effect of USP44 on USP7 levels. HEK293T cells were transfected with MYC‐FOXP3. Cells also received combinations of FLAG‐USP7 and varying doses of pLVX‐USP44 encoding plasmids. FOXP3 was immunoprecipitated using anti‐MYC, and immunoblots were analyzed using anti‐FLAG, anti‐USP44, or anti‐MYC antibodies.Effect of USP44 and USP7 co‐expression on FOXP3 ubiquitination. HEK293T cells were transfected with plasmids encoding MYC‐FOXP3, either FLAG‐USP44 or FLAG‐USP7 or both, His‐ubiquitin. Cells were treated with 20 μM MG132 for 4 h before being harvested, lysed, and incubated on Ni‐NTA beads to pull down ubiquitinated FOXP3, which was visualized by immunoblotting using antibodies specific for MYC.(left panel) iTregs were polarized from human naïve CD4^+^ T cells over 7 days (*n* = 3 donors/experiment) receiving control (shCK), or shRNA knockdown constructs targeting USP44 (shUSP44‐1, ‐2), or USP7 (shUSP7‐1, ‐2), or both shRNA constructs. Freshly isolated nTregs were treated with either control or shRNA constructs. After transduction with lentivirus for 48 h, cells were harvested and run SDS–PAGE gels. Protein levels were visualized by immunoblotting using antibodies specific for FOXP3, USP44, USP7, and GAPDH. Shown are representative findings from three independent experiments (biological replicates). Source data are available online for this figure.

Previously, the DUB USP7 was shown to stabilize the FOXP3 protein pool and the suppressive function of Tregs (van Loosdregt *et al*, 2013). In the present study, we found that another DUB is capable of deubiquitinating FOXP3 to bolster expression of this important transcriptional regulator. We therefore investigated whether or not USP44 co‐operates with USP7 to deubiquitinate and preserve FOXP3. Co‐IP experiments revealed that USP44 and USP7 associated with each other in a complex (Fig 4A). Furthermore, increasing USP44 levels appeared to augment FOXP3 protein levels (Fig 4B). Given that FOXP3 is a shared target for USP44 and USP7, we hypothesized that these DUBs cooperate to stabilize FOXP3. As expected, USP44 or USP7 expression alone reduced ubiquitination of FOXP3, while co‐over‐expression of both USP44 and USP7 almost completely eliminated FOXP3 polyubiquitination, suggesting a synergistic role of these two DUBs (Fig 4C).

The cooperation between USP44 and USP7 was also seen in human iTregs. As previously mentioned, knocking down USP44 expression in iTregs reduces the level of FOXP3 in iTregs. Silencing USP7 expression achieved a similar, but less dramatic effect. Combined USP7/USP44 knockdown, however, even further enhanced FOXP3 degradation (Fig 4D, left panel). Interestingly, in isolated nTregs, USP7 silencing had a greater effect than USP44 silencing (Fig 4D, right panel). These results suggest that, while partially redundant in their ability to preserve the FOXP3 protein pool, these two DUBs are differentially important across Treg subsets. USP7, as previously shown, appears required for optimal stability of established nTregs, while USP44 may be singularly important to sustain FOXP3 in iTregs. Taken together, these results suggest that a degree of cooperation exists between DUBs capable of stabilizing FOXP3 against ubiquitin‐mediated degradation.

### USP44 modulates Treg cell function *in vitro*

**Figure 5:**
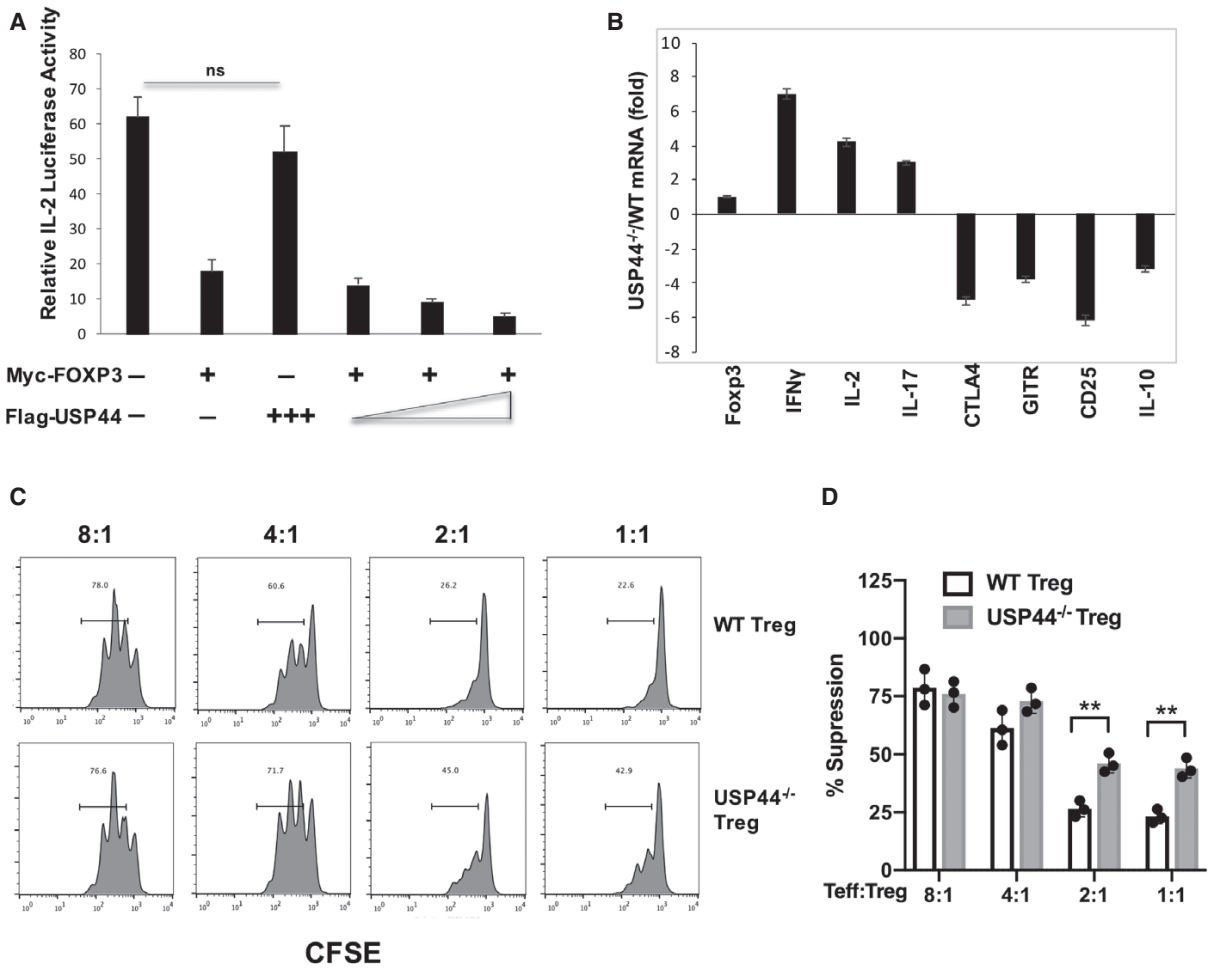
Effects of USP44 on FOXP3‐mediated gene regulation and Treg function *in vitro* AJurkat T cells were transfected with the indicated combinations and relative doses of expression constructs encoding MYC‐FOXP3, FLAG‐USP44 along with an IL‐2 promoter‐driven firefly luciferase reporter. After 8 h of stimulation with PMA/ionomycin, cells were lysed and luciferase activity, which was observed and normalized to Renilla luciferase activity.BTregs (CD4^+^/CD25^High^) were recovered from the lymph nodes and spleens of wild‐type C57BL/6 (WT) and USP44^−/−^ mice by FACS purification. Cells were activated with anti‐CD3/CD28 agonist antibodies overnight, and RNA was isolated, and cDNA was generated from these samples for the measurement of several signature Treg‐associated transcripts by qRT–PCR. The relative expression level of these by WT and KO‐derived Tregs is shown.C, DAnalysis of the *in vitro* suppressive function of Tregs isolated from WT mice or age‐ and sex‐matched USP44^−/−^ mice (*n* = 3/group/experiment). Tregs were mixed with CFSE‐labeled responder naïve T cells from CD45.1^+^ C57BL/6 mice (*n* = 3/experiment) for 3 days. CFSE dilution in CD45.1^+^ T cells was observed by flow cytometry, and the percent suppression of responder cell proliferation by the co‐cultured Tregs was determined.Data information: Data in panels (A), (B), and (D) depict mean values measured across three biological replicates ± SEM, while (C) is representative of at least three independent experiments (biological replicates). ns, not significant, **P < 0.02, by Student's *t*‐test.

Given the importance of USP44 for stabilizing FOXP3, it stands to reason that this DUB is needed for proper Treg function. Using a luciferase‐based reporter assay for IL‐2 expression, we found that the expected repression of the *IL2* gene by FOXP3 could be augmented by over‐expression of USP44. This enhanced suppression of *IL2* transcription was seen alongside levels of FOXP3 protein that corresponded dose‐wise to the degree of USP44 over‐expression in Jurkat T cells (Fig 5A). Assessing the gene expression pattern changes triggered by the knockout of USP44 in Tregs (from USP44^−/−^ mice) revealed disruption in the typical patterns of FOXP3‐governed, Treg‐associated genes. Specifically, transcripts encoding the effector cytokines IL2, IL‐17, and IFN‐γ were elevated, while those for the Treg‐associated factors CTLA‐4, CD25, GITR, and IL‐10 were depressed in Usp44^−/−^ Tregs compared to WT Tregs (Fig 5B). Such a destabilization in Treg gene expression was likely a direct result of undermining USP44‐facilited stabilization of the FOXP3 protein pool, as USP44 deficiency had little‐to‐no effect on Foxp3 mRNA levels (Fig 5B).

The above results predict that in the absence of USP44, the molecular machinery necessary for the suppressive function of Tregs should be lacking. To test the effect of USP44 deficiency on Treg function, an *in vitro* suppression assay was performed using Tregs from WT and Usp44^−/−^ mice. We found that USP44^−/−^ Tregs display less suppressive activity than their wild‐type‐derived counterparts (Fig 5C and D). These findings clearly suggest a supportive role for USP44 in supporting Treg suppressive function.

### USP44‐deficient Tregs are less suppressive than wild‐type Tregs *in vivo*

**Figure 6:**
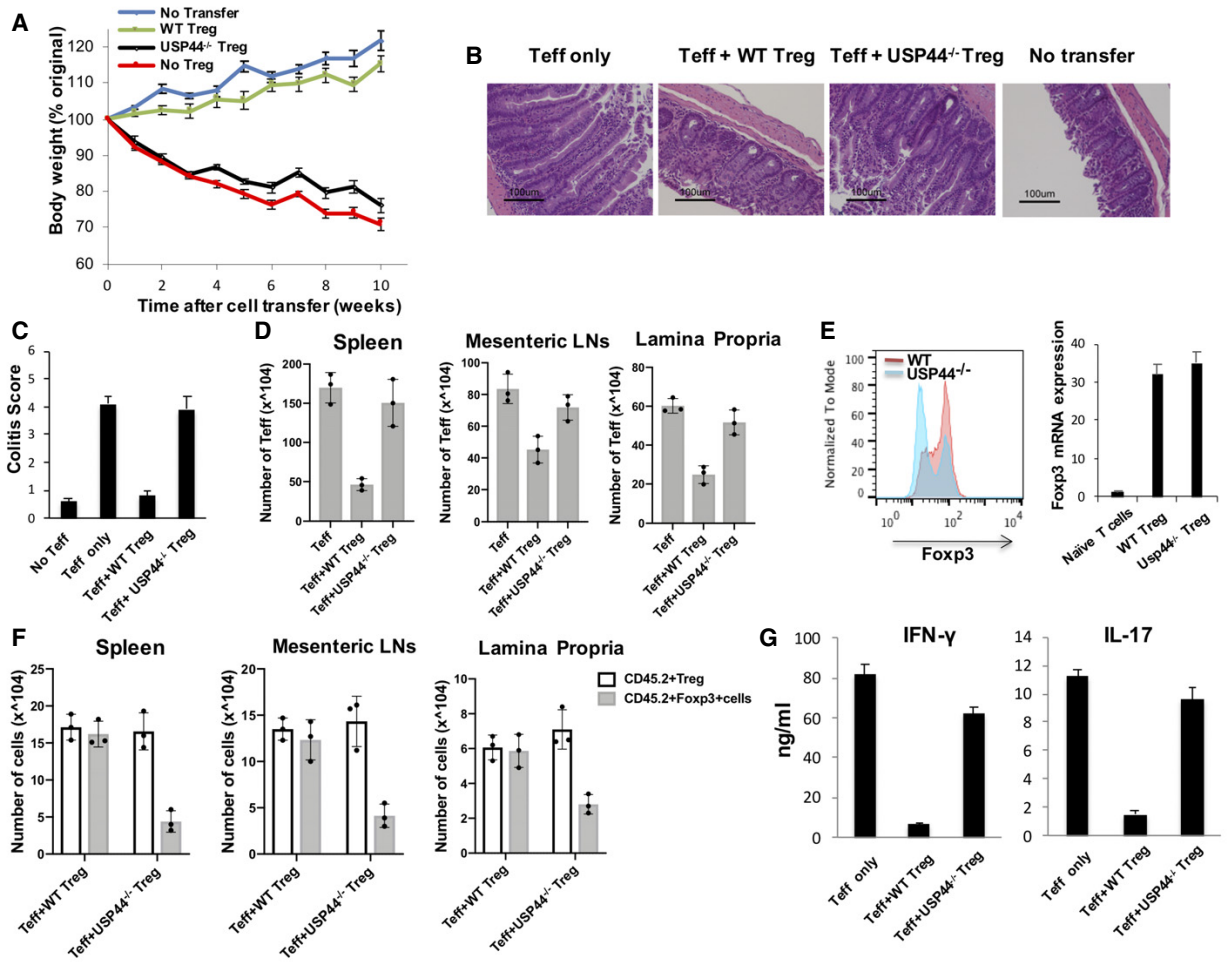
USP44 modulates Treg cell activity *in vivo* Colitis was induced by intravenous coinjection of CD4^+^ CD25‐CD62L^high^ T cells and congenically distinct (CD45.2) CD4^+^ CD25^high^ Treg cells into Rag2^−/−^ mice (*n* = 8/group; 1 × 10^6^ and 2 × 10^5^ cells per Rag2^−/−^ recipient, respectively). Changes in body weight over time were monitored and are expressed as a percentage of the original weight.Representative photomicrographs of the distal colon of Rag2^−/−^ mice after T cell transfer. 10 weeks after transfer, mice were euthanized, and colons were harvested, fixed in 10% buffered formalin, and processed for standard H&E staining prior to histological analysis. (i)‐ (iv) present bright‐field micrographs (100×).H&E slides were scored in a blinded fashion, and colon pathology was scored as described in the Materials and Methods section. Shown are mean scores for each treatment group.Spleen, mesenteric, and lamina propria lymph node cells were isolated from the mice 10 weeks after adoptive T cell transfer, and the number of Teff cells was determined by flow cytometry.Expression of FOXP3 protein and mRNA by adoptively transferred Tregs. Mesenteric lymph nodes were excised from the recipients of WT and USP44^−/−^ Tregs in A. FOXP3 protein was detected by intracellular immunostaining and flow cytometry (left). Shown are events within the gate for transferred Tregs (CD4^+^/CD45.1^+^). Transferred Tregs were also recovered from mesenteric lymph node cell suspensions by staining for the congenic marker and CD4 followed by FACS, and *Foxp3* mRNA was measured in these recovered Tregs by RT–PCR (right).Cell numbers of injected (CD45.2^+^) Treg cells and FOXP3^+^ cells were determined by flow cytometry.CD4^+^ T cells were recovered from suspensions of lamina propria‐infiltrating leukocytes. These lymphocytes were stimulated *ex vivo* by PMA/Ionomycin cocktail in the presence of brefeldin A, and IFN‐γ and IL‐17 production by these lymphocytes was analyzed by ELISA.Data information: Panel (A) shows the mean weight changes for each group in a representative experiment. Panel (B) depicts representative micrographs from all colons processed (*n* = 8/group/experiment), and all others depict the mean results of at least three experiments ± SD (panel (C)) or ± SEM (all others).

In order to determine the importance of USP44 for *in vivo* Treg function, we utilized a T cell‐induced, mouse model of colitis. Briefly, naïve (CD62L^high^/CD25‐) CD4^+^ T cells isolated from wild‐type donors by FACS and were injected intraperitoneally into lymphopenic Rag2^−/−^ mice along with congenically distinct Treg cells isolated from wild‐type or USP44^−/−^ mice. Changes in the body weight of mice receiving these cells, as well as recipients of naïve CD4^+^ only and untreated mice, were assessed weekly. Adoptive transfer of wild‐type Tregs, as expected, prevented the development of severe, progressive wasting caused by naïve CD4^+^ expansion in Rag2^−/−^ mice. In contrast, USP44‐deficient Tregs were less effective at rescuing recipient mice from colitis (Fig 6A). Pathology scoring of H&E‐stained colon tissue sections harvested 10 weeks post‐transfer also suggested that USP44^−/−^ Tregs do not control colon inflammation as well as their wild‐type counterparts (Fig 6B and C). Additionally, the spleen‐, lymph node‐, and lamina propria‐infiltrating leukocytes of mice receiving USP44^−/−^ derived Tregs were found to contain heightened numbers of colitogenic effector cells compared to the tissues of wild‐type Treg recipients, closely resembling the mice not receiving any Tregs at all (Fig 6D).

In light of USP44's role in the deubiquitination and stabilization of FOXP3 protein revealed by our *in vitro* experiments, we assessed levels of the transcription factor in the original transferred Tregs. While most wild‐type Tregs recovered from the gut‐draining lymph node of recipient mice retained high expression of FOXP3, more than half of USP44‐deficient Tregs lost expression of FOXP3 (Fig 6E, left). Notably, despite the low levels of FOXP3 protein signal detected in the recovered USP44^−/−^ Tregs, the *Foxp3* transcript levels seen in these cells were comparable to those observed in transferred wild‐type Tregs (Fig 6E, right) supporting a role for USP44‐mediated support of FOXP3 expression at the protein level. Furthermore, the number of FOXP3‐expressing cells in the originally transferred Treg compartment (Thy1.2^+^) was dramatically reduced in recipients of USP44^−/−^ derived Tregs. In contrast, wild‐type Tregs largely retained FOXP3 across multiple tissues (Fig 6F). These findings illustrate the widespread instability of FOXP3 expression in the face of inflammation when USP44 is lacking. Supporting this, pro‐inflammatory cytokine production by T effector cells was enhanced in the group where Tregs lacked USP44 (Fig 6G).

Corroborating results were obtained in a widely used chemically induced colitis model. Here, USP44^fl/fl^Foxp3‐yfp‐Cre^+^ mice and wild‐type control (Foxp3‐yfp‐Cre^+^) mice received DSS in their drinking water (2.5%) for 7 days followed by normal water for an additional 2 days. As expected, DSS triggered pronounced weight loss in both groups, which were similar in body weight at the start of the experiment (Appendix Fig S4A). However, mice with USP44‐deficient Tregs were prone to more exacerbated disease as evidenced by the significantly more severe weight loss and gut histopathology seen in this group relative to wild‐type controls (Appendix Fig S4B–D) indicative of poor immune control. Flow cytometric analysis revealed that conditional knockout mice also harbored fewer FOXP3^+^ Tregs among their colon lamina propria‐infiltrating CD4^+^ T cells (Appendix Fig S4E). *Foxp3* mRNA levels were also measured in Tregs recovered from the colon‐infiltrating leukocytes of these mice by FACS (CD4^+^/yfp^+^). In line with a protein‐specific role for USP44 in the stabilization of FOXP3 and Treg function, we found similarly abundant transcript levels in Tregs from all groups, mirroring the high levels seen in purified nTregs (a positive control), but not naïve CD4^+^ T cells (a negative control) despite the distinct FOXP3 protein levels and disparate ability to restrain inflammation of these cells (Appendix Fig S4F). Flow cytometry also revealed the frequencies of pro‐inflammatory cytokine (IFNγ, IL‐17) producers were elevated in the colons of Usp44^fl/fl^Foxp3Cre^+^ mice relative to wild‐type controls in this model (Appendix Fig S4G). Collectively, these *in vivo* results support a major role for USP44 in the preservation of FOXP3 expression and Treg function *in vivo*.

### Treg‐specific USP44 deficiency results in stunted tumor growth and enhanced anti‐tumor immunity

**Figure 7:**
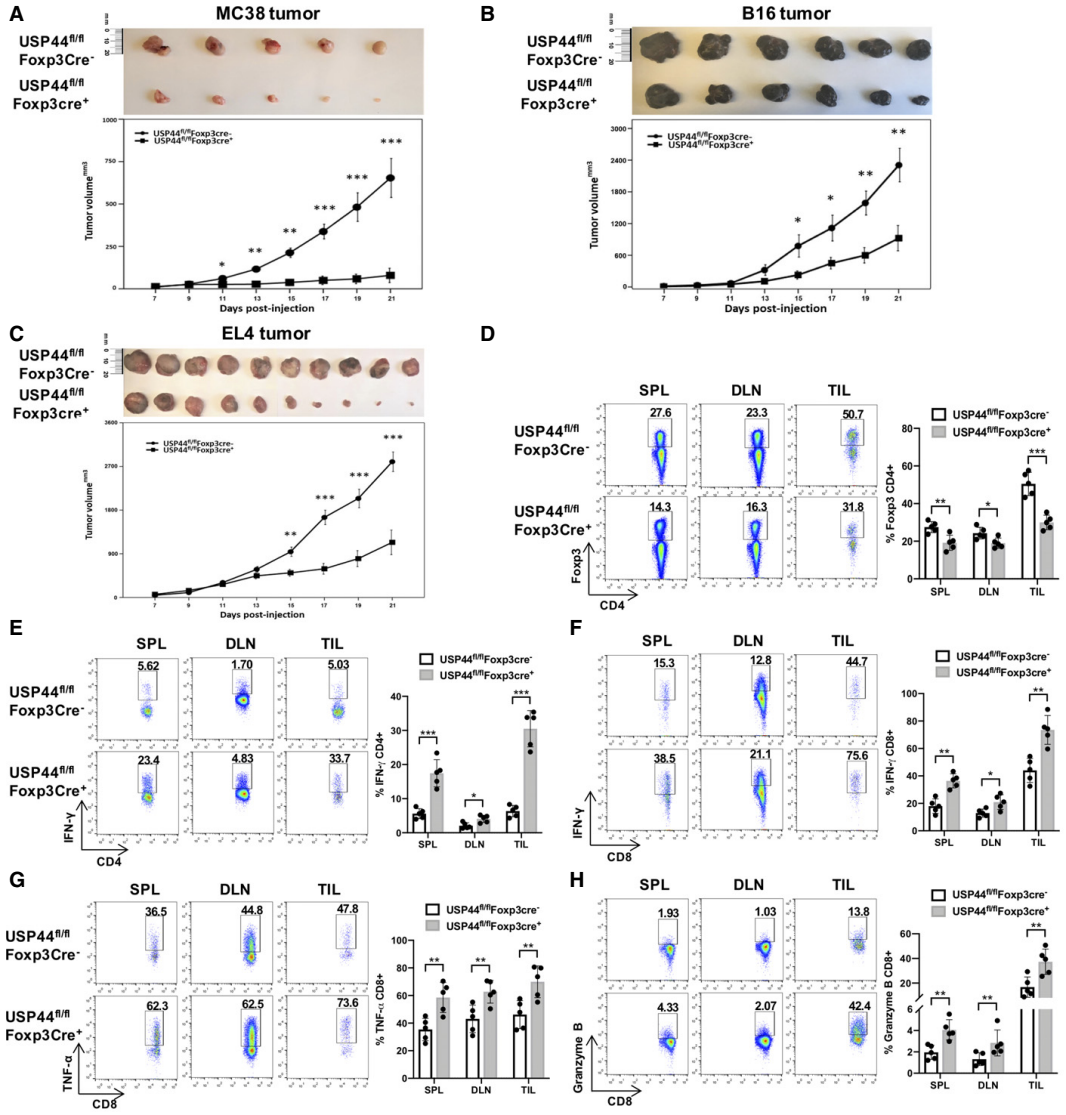
Treg‐specific USP44 deficiency stunts tumor growth and enhances anti‐tumor immunity A–CMC38 colon carcinoma, B16F10 melanoma, and EL4 thymoma cell lines were s.c. injected into the shaved flanks of Usp44^fl/fl^Foxp3Cre^+^ mice and their wild‐type (Usp44^fl/fl^Foxp3Cre‐) littermates (1 × 10^5^ cells/mouse; *n* = 5–9 mice/group). Tumor dimensions were measured every 2 days, and changes in the mean tumor volume over time were observed (lower panels). Upper panels depict representative photographs of excised tumors.E21 days post‐implantation, proportions of FOXP3^+^ Tregs in the CD4^+^ compartment of all MC38 tumor‐bearing mice in each experiment were found by flow cytometry. Suspensions of cells obtained from spleen, tumor‐draining lymph nodes, and tumor‐infiltrating leukocytes (TILs) were stained for surface CD4 and intracellular FOXP3 prior to analysis by flow cytometry.E, FFrequencies of CD4^+^ and CD8^+^ cells producing the pro‐inflammatory cytokine IFNγ were found among the tissues of tumor‐bearing mice in the MC38 model by intracellular cytokine staining (Kralovics *et al*, *2005*) and flow cytometry after *ex vivo* re‐stimulation of recovered leukocytes by PMA and ionomycin in the presence of brefeldin A.G, HThe capacity of CD8^+^ cells for effector function in MC38 tumors was further assessed by measuring (G) production of TNFα by ICS and (H) intracellular straining of the cytotoxic mediator granzyme B followed by flow cytometry.Data information: Lower panels of (A‐C) depict mean tumor volumes over time ( ± SEM), and the flow cytometry plots depicted in panels (D‐H) are representative of all mice analyzed over 5 trials. Data shown are representative findings from 3 biological replicates. *P < 0.05, **P < 0.02, ***P < 0.002; Student's *t*‐test.

We next set out to test the notion that USP44 expression by Tregs contributes to their pathological suppression of anti‐tumor immunity in cancer models. Although it has been reported that mice lacking Usp44 were prone to the development of spontaneous tumors due to the effect to mitotic checkpoint and chromosome lagging (Zhang *et al*, 2012; Mosbech *et al*, 2013), we observed that tumor growth was significantly slower in the Usp44‐null mice compared to their littermate, which suggests a complex role for USP44 biology in tumor‐bearing mice. To further dissect the role of USP44 in Tregs, we generated mice lacking Treg‐specific USP44 expression. Indeed, subcutaneous (s.c.) challenge of Usp44fl/flFoxp3Cre^+^ mice with the implantable MC38 colon carcinoma cell line resulted in substantially delayed tumor growth compared to that seen in wild‐type control mice (Fig 7A). A lack of Treg‐specific USP44 expression also significantly stunted progression of s.c. B16F10 melanomas and EL4 thymomas (Fig 7B and C)—results both supporting a pro‐tumor role for this DUB and compatible with a significant contribution to pathological tolerance in the cancer setting.

In line with this notion, analysis of FOXP3^+^ CD4^+^ T cell frequencies across the tissues of MC38 tumor‐bearing wild‐type and Usp44^fl/fl^Foxp3Cre^+^ mice revealed marked reductions in the relative size of the Treg pool when USP44 expression was lacking in these cells (Fig 7D). This lower Treg presence in Treg‐specific USP44knockout mice was accompanied by enhanced levels of pro‐inflammatory cytokine production within the CD4^+^ and CD8^+^ T cell compartments of the spleen, tumor‐draining lymph nodes, and tumors. Specifically, higher proportions and numbers of IFN‐gamma‐producing CD4^+^ and CD8^+^ T cells were observed in Usp44^fl/fl^Foxp3Cre^+^ mice relative to wild‐type controls (Fig 7E and F). CD8^+^ T cells from tumor‐bearing mice lacking USP44 also were found to express markedly higher levels of TNFα and significantly higher levels of the effector molecule granzyme B than their wild‐type counterparts (Fig 7G and H). These results suggest that Tregs devoid of USP44‐mediated FOXP3 stabilization are ineffective suppressors of anti‐tumor immune responses. They also implicate the action of the DUB in the cancer setting may be a significant contributor to the dampening of effective natural or therapeutically induced anti‐tumor immunity. When considered in concert with our findings from mouse inflammatory disease (colitis) models, these results illustrate the important role played by USP44 in promoting Treg‐mediated immune suppression *in vivo*.

## Discussion

In Tregs, stable expression of the transcription factor FOXP3 anchors their characteristic suppressive phenotype. While mechanisms for the control of transcription at the *Foxp3* gene have been and are being studied extensively, recently identified post‐transcriptional and post‐translational events also influence the expression of FOXP3 and the functional stability of Tregs.

Here, we identify a novel stabilizer of the FOXP3 protein pool in USP44. USP44 has been recognized as a critical regulator of the spindle checkpoint, which delays cell entry into anaphase by deubiquinating Cdc20 (Stegmeier *et al*, 2007). Previous studies showed that USP44 localizes both to the cytoplasm and the nucleus of different cell lines with different targets that play different roles. USP44 has deubiquitinating enzyme activity involved in centrosome positioning, and it is known to counteract the DNA double‐strand break response (Zhang *et al*, 2012; Mosbech *et al*, 2013). It was also shown that it has intranuclear localization and enhances the malignancy of glioma via an association with securin (Zou *et al*, 2017). USP44 was also found to be a negative regulator of H2B ubiquitylation (Fuchs *et al*, 2012; Lan *et al*, 2016) by mainly associating with chromatin and forming a complex with N‐COR subunits in the nucleus. Recently, a portion of cytoplasmic USP44 was reported to migrate to membranes after viral infection to associate with MITA (Zhang *et al*, 2020). To our knowledge, a role for USP44 in Tregs and the regulation of immune responses has previously not been reported.

In this study, USP44 was found to associate with the FOXP3 protein complex in the nucleus and to target K48‐linked polyubiquitin modifications for removal from this critical Treg transcription factor with consequences for both the induction of new FOXP3^+^ Tregs and the function of established Tregs *in vitro* and *in vivo*. Importantly, the functional defects seen in Tregs upon USP44 deletion were linked to the increased instability of FOXP3. Previously, the E3 ligase STUB1 was found to reduce the FOXP3 pool through the addition of degradation‐instigating K48‐type polyubiquitin chains (Chen *et al*, 2013), while a specific deubiquitinating enzyme, USP7, was found to stabilize FOXP3 expression and function by counteracting this process (van Loosdregt *et al*, 2013). Our current findings suggest that USP44, another DUB without an appreciated immunoregulatory role, can act as a distinct force to support FOXP3 expression and both Treg phenotype and function. Expression of this DUB is activated by TGF‐β/SMAD signaling, promoting its interaction with and removal of K48‐linked polyubiquitin modifications from FOXP3, thereby preventing the proteasomal degradation of the transcription factor.

The ability of USP44 to promote FOXP3 expression was evident in our assessment of iTreg induction under limiting TGF‐beta levels. In these experiments, USP44 deficiency resulted in a sizable population of FOXP3‐negative cells, an observation that, at first glance, could be viewed as more in line with a prevention of FOXP3 up‐regulation than the absence of a stabilizer of the FOXP3 protein pool. While we cannot decisively rule out that our findings in part stem from a prevention of up‐regulation of FOXP3 through some uncharacterized USP44‐mediated event, a lack of any detectable FOXP3 in this assay is not incompatible with a major role for USP44 in the stabilization of the FOXP3 protein pool. For instance, it is possible that the FOXP3‐negative T cells in USP44^−/−^ iTreg cultures represent cells that have initiated a level of FOXP3 expression that is quickly lost during the early stages of iTreg differentiation without the stabilizing influence of USP44. This could result in an “all‐or‐nothing scenario” like the one we observe. Indeed, since FOXP3 has been suggested as being able to perpetuate a positive feedback on transcriptional activity at the *FOXP3* locus (Zheng *et al*, 2010), it is possible that induction of an unstable FOXP3 pool in the absence of USP44, under some conditions, could result in a failure to maintain both, transcript and protein levels. Future studies using reporter mice that will enable the tracking of FOXP3 transcriptional fate may shed some light on this topic. However, research tools useful for the proper tracking of the potentially dynamic regulation of FOXP3 protein levels in individual developing iTregs are presently not available.

In the present study, we also found that USP44 appears to cooperate with USP7 in the stabilization of the FOXP3 protein pool (summarized in Appendix Fig S5). While this cooperation appeared prominent in iTregs, it was, however, less critical in nTregs, which seemed more dependent upon USP7 activity. Since recent studies show that USP7 can affect Treg function by regulating the stability of TIP60, a key HAT in Tregs, rather than by deubiquitinating FOXP3 directly (Wang *et al*, 2016, 2017), we also explored the possibility that USP44 and USP7 might cooperate in TIP60 regulation. Indeed, we found that USP44 can promote the stability of TIP60 in a dose‐dependent manner. This finding suggests that in addition to stabilizing FOXP3 protein levels, USP44 may have an additional pro‐Treg role. The relative importance of these roles remains uncertain; however, an ongoing investigation is aimed at achieving a better understanding of the USP44/USP7/TIP60 interactions. Future studies will also explore in‐depth the potential cell‐type‐specific roles for these DUBs as well as their interplay with FOXP3‐targeting E3 ligases, and the therapeutic potential of modulating the activity of USP44 and other factors involved in the post‐translational regulation of FOXP3.

Particularly relevant to the development of new and potent anti‐cancer immunotherapies are strategies aimed at undermining tolerance and unleashing the anti‐tumor immune response. The poor tumor growth and robust anti‐tumor immune mobilization seen in Usp44^fl/fl^ Foxp3Cre^+^ mice illustrate how FOXP3‐stabilizing DUBs like USP44 may be tempting molecular targets for such future therapy.

Our findings suggestive of a pro‐tumor role for Treg‐specific USP44 are particularly interesting in light of past studies of USP44 in the cancer setting. The literature presents a generally complicated picture of this enzyme's role in cancer. For example, Zhang *et al* found that global knockout of this enzyme has been reported to facilitate tumorigenesis by allowing compromised centrosome separation and chromosome segregation errors during cell division. As such, USP44‐deficient mice are more susceptible to the spontaneous development of tumors (particularly lung adenomas) than wild‐type mice when examined at 15 months of age—a finding in line with an observed link between low USP44 expression levels and poor outcomes in lung cancer (Zhang *et al*, 2012). Another recent study found that USP44 deubiquitinates and stabilizes the expression an activity of the histone methyltransferase EZH2 in prostate tumor cells as well as oncogenic mutants of this epigenetic factor with pro‐tumor results. In contrast, knockdown of USP44 in prostate cancer cells reduced tumorigenesis (Park *et al*, 2019). USP44 is also over‐expressed by subsets of T cell leukemias where it is thought to induce chromosomal instability (Zhang *et al*, 2011). Additionally, USP44 was shown to be important for breast cancer cell line growth *in vitro* (Lan *et al*, 2016), and in other studies, USP44 over‐expression was reported in high grade gliomas and linked to poor outcomes (Zou *et al*, 2017). Still other studies suggest that USP44 is widely down‐regulated in other human cancer types (e.g., colorectal cancer) (Sloane *et al*, 2014), and a role for USP44 as a tumor suppressor has also been described in pancreatic cancer (Yang *et al*, 2019).

Notably, the potential role of USP44 as a regulator of the anti‐tumor immune response was not explored in any of these studies, and, indeed, the ability of this and other DUBs to control FOXP3 expression and Treg function in cancer has yet to be explored in detail. Our present findings suggest that targeting factors such as USP44 and USP7 that promote FOXP3 expression at the protein level is a strategy that offers exciting possibilities for the fine‐tuning of immune responses in cancer. A major advantage of such an approach lies in the significant disruption of Treg function that can be achieved largely without effect on the transcription of *Foxp3* message. It is conceivable that with the transcriptional “blueprints” of the Treg phenotype intact, re‐establishment of Treg function and immune moderation is possible upon conclusion of therapy after successful correction of dysregulated immune responses—a scenario unlike that resulting from wholesale Treg depletion. This and the growing body of reports describing important modes of post‐translational regulation of both FOXP3 and Treg function provide considerable motivation for the necessary continued exploration of these pathways and their modulation.

## Materials and Methods

### Animals

All animal experiments were performed in specific‐pathogen‐free, Helicobacter‐free facilities in the Johns Hopkins Animal Resource Center following national, state and institutional guidelines. Global USP44‐null mice and Usp44fl/fl mice on a C57BL/6 background were previously described (Zhang *et al*, 2012). Age‐ and sex‐matched wild‐type C57BL/6 mice maintained in the same facility were used as controls for these mice. The Usp44fl/fl mouse was generated by inserting Loxp sites flanking exon 1. This strain was further back crossed to C57BL/6 mouse for 8 generation to obtain the Usp44fl/fl in pure C57BL/6 background, which was confirmed by chromosome typing. Transgenic mice with loxP sites flanking the Usp44 gene (Usp44 “Floxed” mice) were crossed to cell‐type‐specific Cre recombinase‐expressing lines in order to generate cell lineage specific knockout mice (Usp44^fl/fl^Foxp3Cre^+^). Unless otherwise indicated, co‐housed, Usp44^fl/fl^Foxp3Cre‐ littermates were used as “wild‐type” controls in studies utilizing conditional USP44 knockout mice. The purity of each strain's genetic background was not experimentally verified. Additional strains including Rag2^−/−^ mice and Foxp3‐ires‐GFP reporter mice, both on a C57BL/6 background, were initially purchased from the Jackson Laboratory, and breeding colonies were maintained in house. All animal protocols were approved by the Johns Hopkins Animal Care and Use Committee.

### Plasmids and antibodies

Human FOXP3a and USP44 were cloned into the pIP‐HA2/MYC2/FLAG2 vector as described previously. The pLVX‐IRES‐EGFP vector was a gift from Zhong Huang (Institut Pasteur of Shanghai, Chinese Academy of Sciences). The pLKO.1 vector for shRNA expression was purchased from Open Biosystems and modified for custom‐made shRNA insertion. Antibody recognizing USP44 was obtained from Abmart (China). Anti‐USP7 antibodies were obtained from Cell Signaling. Fluorochrome‐labeled antibodies for flow cytometry against murine and human T cell surface marker and intracellular FOXP3 were purchased from eBioscience (USA), as was a specialized buffer kit for FOXP3 staining, which was used according to the manufacturer's instructions. Anti‐HA and anti‐MYC antibodies as well as protein A/G‐agarose beads were purchased from Santa Cruz (USA), and anti‐FLAG antibodies were obtained from Sigma (USA) and anti‐GAPDH antibodies (1C4, Sungene Biotech). MG132 was purchased from Merck (USA). The primers for USP44‐C282S were generated by Sunny Biotechnology Co. Ltd. (Shanghai) as follows:

USP44‐C282S‐F: 3′‐GAGAAATTTGGGAAATACTAGCTATATGAATTCTGTTC‐5′
USP44‐C282S‐R: 3′‐GAACAGAATTCATATAGCTAGTATTTCCCAAATTTCTC‐5′

TRIzol reagent (Invitrogen) and cDNA reverse transcription kits (TaKaRa) were used as per the manufacturer's instructions. LPS‐EK was purchased from *In vivo* Gene (USA).

### Cell culture and transfection

HEK 293T cells were cultured in Dulbecco modified Eagle medium (DMEM) containing 10% fetal bovine serum (FBS) and transfected using polyethylenimine (PEI) (Polysciences) according to the manufacturer's instructions. Human‐derived Jurkat T cells were maintained in RPMI 1640 medium containing 10% FBS. Transfection of Jurkat cells with plasmid DNA was performed by electroporation on a Gene Pulser X cell apparatus (Bio‐Rad Laboratories, USA). Jurkat T cells were activated using PMA/ionomycin or soluble antibodies against CD3 (1 μg/ml, Hit3a, BioLegend, USA) and CD28 (1 μg/ml, CD28.2, BioLegend, USA). Cell lines were obtained from the laboratory of Drew Pardoll (Johns Hopkins) and were not verified experimentally.

### Immunoprecipitation and immunoblotting

For immunoprecipitation experiments, the indicated transfected cell lines and primary cells were harvested from culture and washed with ice‐cold 1× PBS and lysed in RIPA buffer (50 mM Tris–HCl, pH 7.5; NaCl, 135 mM; 1% NP‐40; 0.5% NaDOC; 21 mM EDTA,10% glycerol) containing protease inhibitor (1:100, P8340, Sigma‐Aldrich), PMSF (1 mM), and NaF (1 mM) on ice for 30 min. Proteins of interest were immunoprecipitated from cell lysate supernatants with the appropriate antibodies for 1 h with rotation at 4°C followed by addition of protein A/G‐agarose beads at 4°C for an additional 1 h. After washing samples 4 times with RIPA (without NP‐40), an appropriate volume of 2× Laemmli loading buffer was added to the immunoprecipitates, which were then boiled at 100°C for 10 min prior to SDS–PAGE resolution and immunoblot analysis using the indicated antibodies.

### Confocal microscopy

Jurkat T cells stably expressing an expression construct encoding hemagglutinin‐tagged FOXP3 (Jurkat‐HA‐FOXP3a cells) (Li *et al*, 2007a) and human Treg cells isolated from healthy donor peripheral blood mononuclear cells (PBMC) were adhered to poly‐L‐lysine‐coated coverslips for 1 h, then fixed in 4% formaldehyde for 0.5 h at room temperature, blocked with 1% BSA and permeabilized with 0.5% Triton X‐100, and incubated with antibodies to USP44 and FOXP3 for 1 h at room temperature. Cells were washed three times with 1× PBS and incubated for 60 min with donkey anti‐mouse‐Dylight 488 and goat anti‐Rabbit‐Dylight 555 in 1 × permeabilization buffer containing 10% normal human serum. Cell nuclei were stained by DAPI dye (1:5,000). And slides were imaged with a laser confocal microscope (LEICA SP5).

### Luciferase‐based reporter assays of gene expression

A luciferase‐based reporter construct for activity at the human *Il2* locus (pGL3‐IL‐2‐luc) was used as previously described (Li *et al*, 2007a) to assay FOXP3‐mediated suppression of effect T cell gene expression. Also, the −341 bp region of the mouse *Usp44* promoter that contained a single SMAD binding site was cloned into the pGL4‐basic vector to generate the pGL4‐USP44‐luc reporter construct used to assess *Usp44* regulation. HEK 293T cells or Jurkat T cells were co‐transfected with the reporter plasmid and a Renilla luciferase encoding plasmid as a control, with and without the indicted FLAG‐SMAD‐encoding constructs by PEI or electroporation. Jurkat T cells were stimulated with PMA and ionomycin for 4 h before been harvesting for the measurement of luciferase activity using a Glo‐Max (Promega) and a dual‐luciferase reporter kit (Promega, USA) as in prior studies (Li *et al*, 2016).

### Quantitative real‐time RT–PCR

Total RNA, inclusive of the small RNA fraction, was extracted from cultured cells with TRIzol reagent (Invitrogen, USA). cDNA was synthesized using a reverse transcriptase kit (TaKaRa, Japan), followed by quantitative real‐time PCR analysis (SYBR green; TaKaRa, Japan). Primers for measuring IL‐2, CTLA‐4, CD25, and GITR transcript levels were previously described (Chen *et al*, 2013). All other primers used are as follows:

USP44‐F: 5′‐TGCCACCTACCTCAGGTTCT‐3′
USP44‐R: 5′‐CTGGTCTGAGGGATTTCAGG‐3′
FOXP3‐F: 5′‐TGCAAAAGGCTTCAGAGACA‐3′
FOXP3‐R: 5′‐CTCTGTTGGGGTGAAAGGAG‐3′
USP7‐F: 5′‐TGTCCGGGACCTGTTAGAAG‐3′
USP7‐R: 5′‐GGCTCGTTGCAGGAGATAAA‐3′
GAPDH‐F: 3′‐GAGTCAACGGATTTGGTCGT‐5′
GAPDH‐R: 3′‐GACAAGCTTCCCGTTCTCAG‐5′

### Generation of ubiquitin mutants and pull‐down assay

To assess the type of ubiquitination targeted by USP44 on the FOXP3 molecule, we generated a collection of ubiquitin lysine mutants. To this end, the 6xHis‐ubiquitin cassette was cloned into the pIP parent vector, and ubiquitin mutants were constructed using a site‐directed mutagenesis kit (Toyobo, Japan). HEK293 cells were transfected with a construct encoding MYC‐labeled FOXP3, pLVX‐USP44, and a construct encoding either a wild‐type or specific mutant ubiquitin. Transfectants were treated with 20 μM MG132 for 4 h before harvest and lysis, and a ubiquitin pull‐down assay was used to recover ubiquitinated proteins as described previously (van Loosdregt *et al*, 2013). In short, cell lysates were incubated with Ni‐NTA agarose beads (Qiagen), washed, and the presence of FOXP3 among the ubiquitinated protein fraction was visualized by immunoblot analysis after SDS–PAGE resolution.

### T cell differentiation and isolation of T cell subsets

Freshly isolated murine Tregs (CD4^+^ CD25^High^, “nTregs”) were isolated from suspensions of lymph node and spleen cells from wild‐type C57BL/6 mice or the indicated transgenic mice and their wild‐type controls (*n* = 5 group/experiment) by FACS. Purified populations of CD4^+^ CD62L^high^CD25^−^ naïve T cells were similarly obtained by FACS. For *in vitro* activation, 1 × 10^6^ cells were cultured in 24‐well plates containing with soluble anti‐CD3 and anti‐CD28 antibodies (2 and 4 μg/ml, respectively; BioLegend). For polarizing naïve CD4^+^ T cells into different T cell subsets, the following cytokines/antibodies were used as in prior studies (Chen *et al*, 2013; Ni *et al*, 2018): for Th1 differentiation: IL‐12 (10 ng/ml), anti‐IL‐4 (10 μg/ml); Th2: IL‐4 (10 ng/ml), anti‐IFNγ (10 μg/ml), and anti‐IL‐12 (10 μg/ml); Th17: IL‐6 (10 ng/ml), TGFβ1 (1.25 ng/ml), IL‐23 (10 ng/ml), IL‐1β (10 ng/ml), anti‐IFNγ (10 μg/ml), and anti‐IL‐4 (10 μg/ml); and induced “iTreg”: TGFβ1 (5 ng/ml, or as indicated) and IL‐2 (100 IU/ml). Naïve CD4^+^ T cells were typically culture under these conditions for 4 days, unless otherwise indicated. Human nTregs (CD4^+^ CD25^high^CD127^low^) and naïve CD4^+^ T cells (CD4^+^CD25^−^CD45RA^+^) were also FACS purified from the buffy coats of healthy PBMC donors (Shanghai Blood Center) as described previously (Gao *et al*, 2012) and in accordance with guidelines for human sample research. Human blood donors for experiments were anonymous. Primary human cells for all experiments were sources from healthy donors or freshly drawn whole blood under a protocol approved by the Shanghai Jiao Tong University Review Board. The informed consent for all human experiments was approved by the Institutional Review Boards of Shanghai Jiao Tong University and Institut Pasteur of Shanghai.

### Chromatin immunoprecipitation assay

ChIP assays were performed using the MAGnify ChIP system (Invitrogen) according to the manufacturer's guidance. Briefly, CD4^+^ iTreg cells were generated as described above and FACS prior to overnight activation with αCD3/αCD28‐conjugated beads. iTregs were then fixed with 2% formaldehyde and sonicated. DNA was immunoprecipitated with anti‐Smad2/3 antibodies (Cell Signaling Technology). The immunoprecipitated chromatin was analyzed on a Roche LightCycler 480 by SYBR Green using the following primers for the murine *Usp44* promoter:

Forward: 5′‐GCACTACATTATGGAATGTG‐3′
Reverse: 5′‐CTAAGTAGAAACTCGTCCGG‐3′

### Lentiviral constructs and transduction

shRNA lentiviral constructs targeting USP44 and USP7 expression (shUSP44, shUSP7), and a control construct (shCK) were introduced into the pLKO.1 delivery vector. These were transfected into HEK 293T cells via calcium phosphate transfection along with the lentivirus packing vector Delta 8.9 and VSVG envelope glycoprotein. Viral supernatants were harvested after 48 h. Primary T cells were transduced with virus along with anti‐CD3/28 stimuli (1:1 cell to bead ratio). The following shRNA sequences were generated at Shanghai Sunny Biotechnology Co. Ltd., and the shRNA sequences used were as below:

shCK: 5′‐ CAACAAGATGAAGAGCACCAA‐3′
shUSP44‐1: 5′‐ ACTGAGAATGGACATTCTAAA‐3′
shUSP44‐2: 5′‐GAGTATCAAGTTAAAGCAGAA‐3′
shUSP7‐1: 5′‐TTGTGGTTACGTTATCAAATA‐3′
shUSP7‐2: 5′‐TCCTAAGGACCCTGCAAATTA‐3′

### T cell‐induced colitis and *in vivo* Treg analysis

Naïve CD4^+^ T cells were isolated from the pooled lymph nodes and spleens of wild‐type C57BL/6 mice (CD45.1^+^; *n* = 5/experiment) by FACS and resuspended in PBS. 4 × 10^5^ naïve T cells were injected intraperitoneally into lymphopenic Rag2^−/−^ mice (also on a C57BL/6 genetic background). Approximately 1 week post‐injection, 2 × 10^5^ congenically distinct (CD45.2), Treg cells freshly isolated from wild‐type C57BL/6 and USP44^−/−^ mice were injected. Changes in body weight were assessed weekly, and upon conclusion of the experiment, colons were removed and fixed in 10% formalin. Five‐micrometer paraffin‐embedded sections were cut and stained with hematoxylin and eosin (H&E). The pathology of colon tissue was scored in a blinded fashion, on a scale of 0‐5 where a grade of 0 was given when there were no changes observed. Changes associated with other grades were as follows: grade 1, minimal scattered mucosal inflammatory cell infiltrates, with or without minimal epithelial hyperplasia; grade 2, mild scattered to diffuse inflammatory cell infiltrates, sometimes extending into the submucosa and associated with erosions, with mild to moderate epithelial hyperplasia and mild to moderate mucin depletion from goblet cells; grade 3, moderate inflammatory cell infiltrates that were sometimes transmural, with moderate to severe epithelial hyperplasia and mucin depletion; grade 4, marked inflammatory cell infiltrates that were often transmural and associated with crypt abscesses and occasional ulceration, with marked epithelial hyperplasia, mucin depletion; and grade 5, marked transmural inflammation with severe ulceration and loss of intestinal glands. Transferred naïve and Treg cell populations were recovered from the indicated tissues and characterized by immunostaining surface markers (CD4, CD45.1, and CD45.2, respectively) and intracellular cytokines and FOXP3. For cytokine analysis, recovered leukocytes were restimulated as previously described (Chen *et al*, 2013).

### DSS‐induced colitis model

USP44^fl/fl^Foxp3Cre^+^ mice and their wild‐type USP44^wt/wt^Foxp3Cre^+^ littermates (*n* = 10/group) were administered a low dose (2.5%) of DSS in their drinking water over a 7‐day period after which normal drinking was given for an additional 48 h. Cohorts of mice receiving normal drinking water throughout the experiment served as negative controls for disease. Changes in mouse body weights in each group were monitored daily throughout the experiment and 9 days after disease induction, leukocytes were recovered from the colon and lymphoid tissues for flow cytometric characterization. The colons of some mice were harvested, fixed with 10% buffered formalin and processed for sectioning, H&E staining, and pathological scoring by a blinded observed as previously described.

### Murine tumor models

B16F10 melanoma, MC38 colon carcinoma, and EL4 thymoma cell lines were passaged *in vitro* before s.c. implantation into the shaved flanks of Usp44^fl/fl^Foxp3Cre^+^ mice or their wild‐type (WT) Usp44^fl/fl^Foxp3Cre‐ littermates (1 × 10^5^ cells/mouse; *n* = 5–9 mice/group). The development and progression of implanted tumors was monitored by digital caliper measurements of tumor length and width taken every 2–3 days for the duration of the experiment (21 days post‐injection). Changes in tumor volume over time were thus determined for both groups using the formula: v = (L×W^2^)/2. At the conclusion of MC38 tumor model experiments, the leukocytes infiltrating the tumors and relevant lymphoid tissues of each group were recovered and characterized by flow cytometry.

### Statistical analyses

Data are presented as mean ± SD or SEM as indicated. Student's *t*‐test was used to determine statistically significant differences (*P < 0.05, **P < 0.02, ***P < 0.005) using GraphPad Prism software.

## Author contributions

JY and PW are responsible for generation of research findings reported in paper, designing and performance of experiments, and writing the manuscript; JB, QH, and YB assisted with the designing and performance of experiments; JN helped with human T cell differentiation; YG, EY, JT, YL, CX, XH, JR, XW, JM, YZ, JF, WK, YG, ZC, RL, AT, DL, WG, and SZ provided materials and technical supports essential for this project; JB, S‐GZ, JN, PG, XT, and GS offered valuable scientific feedback and helped with conception of research reported in paper; and HL, FP, and BL conceptualized and designed the studies, carried out data analyses, and wrote the manuscript.

## Conflict of interest

The authors declare that they have no conflict of interest.

## Supporting information



Appendix

Expanded View Figures PDF

Source Data for Expanded View and Appendix

Review Process File

Source Data for Figure 1

Source Data for Figure 2

Source Data for Figure 3

Source Data for Figure 4

## Data Availability

All data generated or analyzed during this study are included in this published article and its supplementary information files. The datasets used and/or analyzed during the current study are available from the corresponding author on reasonable request. No primary datasets have been generated or deposited.

## References

[embr202050308-bib-0003] Bennett CL , Brunkow ME , Ramsdell F , O'Briant KC , Zhu Q , Fuleihan RL , Shigeoka AO , Ochs HD , Chance PF (2001a) A rare polyadenylation signal mutation of the FOXP3 gene (AAUAAA–>AAUGAA) leads to the IPEX syndrome. Immunogenetics 53: 435–439 11685453 10.1007/s002510100358

[embr202050308-bib-0004] Bennett CL , Christie J , Ramsdell F , Brunkow ME , Ferguson PJ , Whitesell L , Kelly TE , Saulsbury FT , Chance PF , Ochs HD (2001b) The immune dysregulation, polyendocrinopathy, enteropathy, X‐linked syndrome (IPEX) is caused by mutations of FOXP3. Nat Genet 27: 20–21 11137993 10.1038/83713

[embr202050308-bib-0005] Brunkow ME , Jeffery EW , Hjerrild KA , Paeper B , Clark LB , Yasayko SA , Wilkinson JE , Galas D , Ziegler SF , Ramsdell F (2001) Disruption of a new forkhead/winged‐helix protein, scurfin, results in the fatal lymphoproliferative disorder of the scurfy mouse. Nat Genet 27: 68–73 11138001 10.1038/83784

[embr202050308-bib-0006] Chen Z , Barbi J , Bu S , Yang HY , Li Z , Gao Y , Jinasena D , Fu J , Lin F , Chen C *et al* (2013) The ubiquitin ligase Stub1 negatively modulates regulatory T cell suppressive activity by promoting degradation of the transcription factor Foxp3. Immunity 39: 272–285 23973223 10.1016/j.immuni.2013.08.006 PMC3817295

[embr202050308-bib-0008] Fontenot JD , Gavin MA , Rudensky AY (2003) Foxp3 programs the development and function of CD4^+^ CD25^+^ regulatory T cells. Nat Immunol 4: 330–336 12612578 10.1038/ni904

[embr202050308-bib-0009] Fu W , Ergun A , Lu T , Hill JA , Haxhinasto S , Fassett MS , Gazit R , Adoro S , Glimcher L , Chan S *et al* (2012) A multiply redundant genetic switch “locks in” the transcriptional signature of regulatory T cells. Nat Immunol 13: 972–980 22961053 10.1038/ni.2420 PMC3698954

[embr202050308-bib-0010] Fuchs G , Shema E , Vesterman R , Kotler E , Wolchinsky Z , Wilder S , Golomb L , Pribluda A , Zhang F , Haj‐Yahya M *et al* (2012) RNF20 and USP44 regulate stem cell differentiation by modulating H2B monoubiquitylation. Mol Cell 46: 662–673 22681888 10.1016/j.molcel.2012.05.023 PMC3374598

[embr202050308-bib-0011] Gao Z , Gao Y , Li Z , Chen Z , Lu D , Tsun A , Li B (2012) Synergy between IL‐6 and TGF‐beta signaling promotes FOXP3 degradation. Int J Clin Exp Pathol 5: 626–633 22977658 PMC3438759

[embr202050308-bib-0012] Gao Y , Tang J , Chen W , Li Q , Nie J , Lin F , Wu Q , Chen Z , Gao Z , Fan H *et al* (2015) Inflammation negatively regulates FOXP3 and regulatory T‐cell function via DBC1. Proc Natl Acad Sci USA 112: E3246–E3254 26060310 10.1073/pnas.1421463112 PMC4485127

[embr202050308-bib-0013] Hori S (2012) The Foxp3 interactome: a network perspective of T(reg) cells. Nat Immunol 13: 943–945 22990900 10.1038/ni.2424

[embr202050308-bib-0014] Jin J , Li X , Gygi SP , Harper JW (2007) Dual E1 activation systems for ubiquitin differentially regulate E2 enzyme charging. Nature 447: 1135–1138 17597759 10.1038/nature05902

[embr202050308-bib-0015] Josefowicz SZ , Lu LF , Rudensky AY (2012a) Regulatory T cells: mechanisms of differentiation and function. Annu Rev Immunol 30: 531–564 22224781 10.1146/annurev.immunol.25.022106.141623 PMC6066374

[embr202050308-bib-0016] Josefowicz SZ , Niec RE , Kim HY , Treuting P , Chinen T , Zheng Y , Umetsu DT , Rudensky AY (2012b) Extrathymically generated regulatory T cells control mucosal TH2 inflammation. Nature 482: 395–399 22318520 10.1038/nature10772 PMC3485072

[embr202050308-bib-0017] Komander D , Rape M (2012) The ubiquitin code. Annu Rev Biochem 81: 203–229 22524316 10.1146/annurev-biochem-060310-170328

[embr202050308-bib-0018] Kralovics R , Passamonti F , Buser AS , Teo SS , Tiedt R , Passweg JR , Tichelli A , Cazzola M , Skoda RC (2005) A gain‐of‐function mutation of JAK2 in myeloproliferative disorders. N Engl J Med 352: 1779–1790 15858187 10.1056/NEJMoa051113

[embr202050308-bib-0019] Lan X , Atanassov BS , Li W , Zhang Y , Florens L , Mohan RD , Galardy PJ , Washburn MP , Workman JL , Dent SYR (2016) USP44 is an integral component of N‐CoR that contributes to gene repression by deubiquitinating histone H2B. Cell Rep 17: 2382–2393 27880911 10.1016/j.celrep.2016.10.076 PMC5131803

[embr202050308-bib-0020] Li B , Samanta A , Song X , Iacono KT , Bembas K , Tao R , Basu S , Riley JL , Hancock WW , Shen Y *et al* (2007a) FOXP3 interactions with histone acetyltransferase and class II histone deacetylases are required for repression. Proc Natl Acad Sci USA 104: 4571–4576 17360565 10.1073/pnas.0700298104 PMC1838642

[embr202050308-bib-0021] Li B , Samanta A , Song X , Iacono KT , Brennan P , Chatila TA , Roncador G , Banham AH , Riley JL , Wang Q *et al* (2007b) FOXP3 is a homo‐oligomer and a component of a supramolecular regulatory complex disabled in the human XLAAD/IPEX autoimmune disease. Int Immunol 19: 825–835 17586580 10.1093/intimm/dxm043

[embr202050308-bib-0022] Li Z , Lin F , Zhuo C , Deng G , Chen Z , Yin S , Gao Z , Piccioni M , Tsun A , Cai S *et al* (2014) PIM1 kinase phosphorylates the human transcription factor FOXP3 at serine 422 to negatively regulate its activity under inflammation. J Biol Chem 289: 26872–26881 25096571 10.1074/jbc.M114.586651 PMC4175328

[embr202050308-bib-0023] Li Y , Lu Y , Wang S , Han Z , Zhu F , Ni Y , Liang R , Zhang Y , Leng Q , Wei G *et al* (2016) USP21 prevents the generation of T‐helper‐1‐like Treg cells. Nat Commun 7: 13559–13559 27857073 10.1038/ncomms13559 PMC5120220

[embr202050308-bib-0025] Liu ZM , Wang KP , Ma J , Guo Zheng S (2015) The role of all‐trans retinoic acid in the biology of Foxp3^+^ regulatory T cells. Cell Mol Immunol 12: 553–557 25640656 10.1038/cmi.2014.133 PMC4579645

[embr202050308-bib-0026] van Loosdregt J , Vercoulen Y , Guichelaar T , Gent YY , Beekman JM , van Beekum O , Brenkman AB , Hijnen DJ , Mutis T , Kalkhoven E *et al* (2010) Regulation of Treg functionality by acetylation‐mediated Foxp3 protein stabilization. Blood 115: 965–974 19996091 10.1182/blood-2009-02-207118

[embr202050308-bib-0027] van Loosdregt J , Fleskens V , Fu J , Brenkman AB , Bekker CP , Pals CE , Meerding J , Berkers CR , Barbi J , Grone A *et al* (2013) Stabilization of the transcription factor Foxp3 by the deubiquitinase USP7 increases Treg‐cell‐suppressive capacity. Immunity 39: 259–271 23973222 10.1016/j.immuni.2013.05.018 PMC4133784

[embr202050308-bib-0030] Luo X , Nie J , Wang S , Chen Z , Chen W , Li D , Hu H , Li B (2015) Poly(ADP‐ribosyl)ation of FOXP3 protein mediated by PARP‐1 protein regulates the function of regulatory T cells. J Biol Chem 290: 28675–28682 26429911 10.1074/jbc.M115.661611 PMC4661383

[embr202050308-bib-0031] Marie JC , Letterio JJ , Gavin M , Rudensky AY (2005) TGF‐beta1 maintains suppressor function and Foxp3 expression in CD4^+^ CD25^+^ regulatory T cells. J Exp Med 201: 1061–1067 15809351 10.1084/jem.20042276 PMC2213134

[embr202050308-bib-0032] Miyara M , Sakaguchi S (2007) Natural regulatory T cells: mechanisms of suppression. Trends Mol Med 13: 108–116 17257897 10.1016/j.molmed.2007.01.003

[embr202050308-bib-0033] Morawski PA , Mehra P , Chen C , Bhatti T , Wells AD (2013) Foxp3 protein stability is regulated by cyclin‐dependent kinase 2. J Biol Chem 288: 24494–24502 23853094 10.1074/jbc.M113.467704 PMC3750148

[embr202050308-bib-0034] Mosbech A , Lukas C , Bekker‐Jensen S , Mailand N (2013) The deubiquitylating enzyme USP44 counteracts the DNA double‐strand break response mediated by the RNF8 and RNF168 ubiquitin ligases. J Biol Chem 288: 16579–16587 23615962 10.1074/jbc.M113.459917 PMC3675593

[embr202050308-bib-0035] Ni X , Tao J , Barbi J , Chen Q , Park BV , Li Z , Zhang N , Lebid A , Ramaswamy A , Wei P *et al* (2018) YAP is essential for treg‐mediated suppression of antitumor immunity. Cancer Discov 8: 1026–1043 29907586 10.1158/2159-8290.CD-17-1124 PMC6481611

[embr202050308-bib-0036] Nie H , Zheng Y , Li R , Guo TB , He D , Fang L , Liu X , Xiao L , Chen X , Wan B *et al* (2013) Phosphorylation of FOXP3 controls regulatory T cell function and is inhibited by TNF‐alpha in rheumatoid arthritis. Nat Med 19: 322–328 23396208 10.1038/nm.3085

[embr202050308-bib-0037] Ono M , Yaguchi H , Ohkura N , Kitabayashi I , Nagamura Y , Nomura T , Miyachi Y , Tsukada T , Sakaguchi S (2007) Foxp3 controls regulatory T‐cell function by interacting with AML1/Runx1. Nature 446: 685–689 17377532 10.1038/nature05673

[embr202050308-bib-0038] Pan F , Yu H , Dang EV , Barbi J , Pan X , Grosso JF , Jinasena D , Sharma SM , McCadden EM , Getnet D *et al* (2009) Eos mediates Foxp3‐dependent gene silencing in CD4^+^ regulatory T cells. Science 325: 1142–1146 19696312 10.1126/science.1176077 PMC2859703

[embr202050308-bib-0039] Park JM , Lee JE , Park CM , Kim JH (2019) USP44 promotes the tumorigenesis of prostate cancer cells through EZH2 protein stabilization. Mol Cells 42: 17–27 30622230 10.14348/molcells.2018.0329 PMC6354053

[embr202050308-bib-0042] Sakaguchi S , Yamaguchi T , Nomura T , Ono M (2008) Regulatory T cells and immune tolerance. Cell 133: 775–787 18510923 10.1016/j.cell.2008.05.009

[embr202050308-bib-0043] Samanta A , Li B , Song X , Bembas K , Zhang G , Katsumata M , Saouaf SJ , Wang Q , Hancock WW , Shen Y *et al* (2008) TGF‐beta and IL‐6 signals modulate chromatin binding and promoter occupancy by acetylated FOXP3. Proc Natl Acad Sci USA 105: 14023–14027 18779564 10.1073/pnas.0806726105 PMC2544572

[embr202050308-bib-0044] Sargin B , Choudhary C , Crosetto N , Schmidt MHH , Grundler R , Rensinghoff M , Thiessen C , Tickenbrock L , Schwäble J , Brandts C *et al* (2007) Flt3‐dependent transformation by inactivating c‐Cbl mutations in AML. Blood 110: 1004 17446348 10.1182/blood-2007-01-066076

[embr202050308-bib-0045] Schlenner SM , Weigmann B , Ruan Q , Chen Y , von Boehmer H (2012) Smad3 binding to the foxp3 enhancer is dispensable for the development of regulatory T cells with the exception of the gut. J Exp Med 209: 1529–1535 22908322 10.1084/jem.20112646 PMC3428940

[embr202050308-bib-0046] Schmidt A , Oberle N , Krammer PH (2012) Molecular mechanisms of treg‐mediated T cell suppression. Front Immunol 3: 51 22566933 10.3389/fimmu.2012.00051 PMC3341960

[embr202050308-bib-0047] Sloane MA , Wong JWH , Perera D , Nunez AC , Pimanda JE , Hawkins NJ , Sieber OM , Bourke MJ , Hesson LB , Ward RL (2014) Epigenetic inactivation of the candidate tumor suppressor USP44 is a frequent and early event in colorectal neoplasia. Epigenetics 9: 1092–1100 24837038 10.4161/epi.29222 PMC4164494

[embr202050308-bib-0048] Stegmeier F , Rape M , Draviam VM , Nalepa G , Sowa ME , Ang XL , McDonald ER III , Li MZ , Hannon GJ , Sorger PK *et al* (2007) Anaphase initiation is regulated by antagonistic ubiquitination and deubiquitination activities. Nature 446: 876–881 17443180 10.1038/nature05694

[embr202050308-bib-0049] Szurek E , Cebula A , Wojciech L , Pietrzak M , Rempala G , Kisielow P , Ignatowicz L (2015) Differences in expression level of helios and neuropilin‐1 do not distinguish thymus‐derived from extrathymically‐induced CD4^+^ Foxp3^+^ regulatory T cells. PLoS ONE 10: e0141161 26495986 10.1371/journal.pone.0141161 PMC4619666

[embr202050308-bib-0050] Tao R , de Zoeten EF , Ozkaynak E , Chen C , Wang L , Porrett PM , Li B , Turka LA , Olson EN , Greene MI *et al* (2007) Deacetylase inhibition promotes the generation and function of regulatory T cells. Nat Med 13: 1299–1307 17922010 10.1038/nm1652

[embr202050308-bib-0051] Thornton AM , Korty PE , Tran DQ , Wohlfert EA , Murray PE , Belkaid Y , Shevach EM (2010) Expression of Helios, an Ikaros transcription factor family member, differentiates thymic‐derived from peripherally induced Foxp3^+^ T regulatory cells. J Immunol 184: 3433–3441 20181882 10.4049/jimmunol.0904028 PMC3725574

[embr202050308-bib-0052] Tran DQ (2012) TGF‐beta: the sword, the wand, and the shield of FOXP3(+) regulatory T cells. J Mol Cell Biol 4: 29–37 22158907 10.1093/jmcb/mjr033

[embr202050308-bib-0053] Visconti R , Palazzo L , Della Monica R , Grieco D (2012) Fcp1‐dependent dephosphorylation is required for M‐phase‐promoting factor inactivation at mitosis exit. Nat Commun 3: 894 22692537 10.1038/ncomms1886 PMC3621406

[embr202050308-bib-0054] Wang L , Kumar S , Dahiya S , Wang F , Wu J , Newick K , Han R , Samanta A , Beier UH , Akimova T *et al* (2016) Ubiquitin‐specific protease‐7 inhibition impairs Tip60‐dependent Foxp3 + T‐regulatory cell function and promotes antitumor immunity. EBioMedicine 13: 99–112 27769803 10.1016/j.ebiom.2016.10.018 PMC5264272

[embr202050308-bib-0055] Wang F , Wang L , Wu J , Sokirniy I , Nguyen P , Bregnard T , Weinstock J , Mattern M , Bezsonova I , Hancock WW *et al* (2017) Active site‐targeted covalent irreversible inhibitors of USP7 impair the functions of Foxp3^+^ T‐regulatory cells by promoting ubiquitination of Tip60. PLoS ONE 12: e0189744 29236775 10.1371/journal.pone.0189744 PMC5728538

[embr202050308-bib-0056] Wu Y , Borde M , Heissmeyer V , Feuerer M , Lapan AD , Stroud JC , Bates DL , Guo L , Han A , Ziegler SF *et al* (2006) FOXP3 controls regulatory T cell function through cooperation with NFAT. Cell 126: 375–387 16873067 10.1016/j.cell.2006.05.042

[embr202050308-bib-0057] Yadav M , Louvet C , Davini D , Gardner JM , Martinez‐Llordella M , Bailey‐Bucktrout S , Anthony BA , Sverdrup FM , Head R , Kuster DJ *et al* (2012) Neuropilin‐1 distinguishes natural and inducible regulatory T cells among regulatory T cell subsets *in vivo* . J Exp Med 209: 1713–1722 22966003 10.1084/jem.20120822 PMC3457729

[embr202050308-bib-0058] Yang C , Zhu S , Yang H , Deng S , Fan P , Li M , Jin X (2019) USP44 suppresses pancreatic cancer progression and overcomes gemcitabine resistance by deubiquitinating FBP1. Am J Cancer Res 9: 1722–1733 31497353 PMC6726996

[embr202050308-bib-0059] Zabransky DJ , Nirschl CJ , Durham NM , Park BV , Ceccato CM , Bruno TC , Tam AJ , Getnet D , Drake CG (2012) Phenotypic and functional properties of Helios+ regulatory T cells. PLoS ONE 7: e34547 22479644 10.1371/journal.pone.0034547 PMC3316700

[embr202050308-bib-0060] Zhang Y , van Deursen J , Galardy PJ (2011) Overexpression of ubiquitin specific protease 44 (USP44) Induces chromosomal instability and is frequently observed in human T‐cell leukemia. PLoS ONE 6: e23389 21853124 10.1371/journal.pone.0023389 PMC3154946

[embr202050308-bib-0061] Zhang Y , Foreman O , Wigle DA , Kosari F , Vasmatzis G , Salisbury JL , van Deursen J , Galardy PJ (2012) USP44 regulates centrosome positioning to prevent aneuploidy and suppress tumorigenesis. J Clin Invest 122: 4362–4374 23187126 10.1172/JCI63084 PMC3533537

[embr202050308-bib-0062] Zhang H‐Y , Liao B‐W , Xu Z‐S , Ran Y , Wang D‐P , Yang Y , Luo W‐W , Wang Y‐Y (2020) USP44 positively regulates innate immune response to DNA viruses through deubiquitinating MITA. PLoS Pathog 16: e1008178 31968013 10.1371/journal.ppat.1008178 PMC6975528

[embr202050308-bib-0063] Zheng Y , Chaudhry A , Kas A , deRoos P , Kim JM , Chu TT , Corcoran L , Treuting P , Klein U , Rudensky AY (2009) Regulatory T‐cell suppressor program co‐opts transcription factor IRF4 to control T(H)2 responses. Nature 458: 351–356 19182775 10.1038/nature07674 PMC2864791

[embr202050308-bib-0064] Zheng Y , Josefowicz S , Chaudhry A , Peng XP , Forbush K , Rudensky AY (2010) Role of conserved non‐coding DNA elements in the Foxp3 gene in regulatory T‐cell fate. Nature 463: 808–812 20072126 10.1038/nature08750 PMC2884187

[embr202050308-bib-0066] Zou Y , Qiu G , Jiang L , Cai Z , Sun W , Hu H , Lu C , Jin W , Hu G (2017) Overexpression of ubiquitin specific proteases 44 promotes the malignancy of glioma by stabilizing tumor‐promoter securin. Oncotarget 8: 58231–58246 28938551 10.18632/oncotarget.16447 PMC5601647

