# Supplementary material for: The deubiquitinase USP44 promotes Treg function during inflammation by preventing FOXP3 degradation
Source: EMBO Rep. 2020 Jul 9;21(9):e50308. doi: 10.15252/embr.202050308 (PMC7507386; doi:10.15252/embr.202050308)
Supplement: Supplementary file 1 — Appendix [file EMBR-21-e50308-s001.pdf]

Appendix- SUPPLEMENTARY MATERIALS

Table of Contents

Appendix Figure S1. Knock down of USP44 in the Jurkat-HA-FOXP3 cell line and primary human Tregs destabilizes FOXP3 and Treg gene expression patterns……......Page2

Appendix Figure S2. Analysis of the leukocyte populations of wild type and global USP44 knockout mice………...Page 3

Appendix Figure S3. USP44 downregulates K48-linked polyubiquitination of FOXP3……….Page 4

Appendix Figure S4. Deletion of USP44 in Tregs renders mice susceptible to more severe disease in the DSS-induced colitis model……….Page 5

Appendix Figure S5. Summarized Model of USP44's role in Foxp3 regulation…………………………………………………………………………………………………………...Page 6

Appendix Figure S1

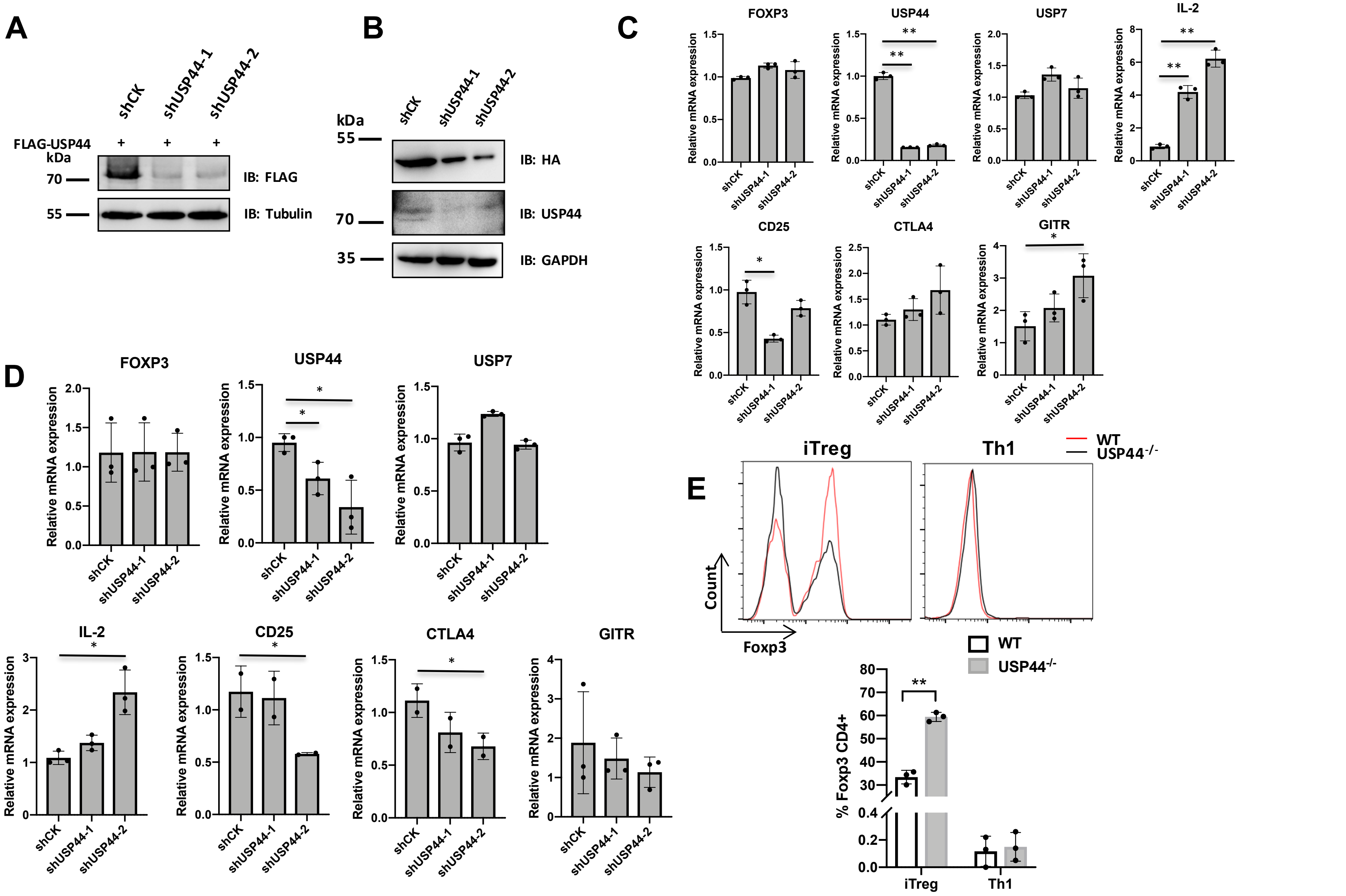

Figure S1. Knock down of USP44 in the Jurkat-HA-FOXP3 cell line and primary human Tregs destabilizes FOXP3 and Treg gene expression patterns. The efficiency of USP44 shRNAs was demonstrated in HEK 293T cells **(A)** and Jurkat-HA-FOXP3 T cells **(B)**. **(A)** HEK 293T cells were transfected with constructs encoding FLAG-USP44 and either pLKO.1-shUSP44-1,2. Levels of labeled USP44 were observed by western blotting using antibodies specific for FLAG. **(B)** USP44 was knocked down in 10^6 Jurkat-HA-FOXP3 cells using shRNA lentivirus containing a puromycin resistance cassette. FOXP3 and USP44 protein level was visualized by western blotting. **(C)** FOXP3, IL-2, CD25, CTLA4, GITR, USP44 and USP7 mRNA levels in puromycin selected cells were analyzed by qRT-PCR. **(D)** Human Tregs were polarized from naïve CD4+ T cells for 7 days, before lentiviral delivery of knock down vectors (shCK control, shUSP44-1, or shUSP44-2 constructs. mRNA expression of FOXP3, USP44, USP7, IL-2, CD25, GITR, and CTLA4 was visualized by qRT-PCR. **(E)** Naïve CD4+T cells from WT and USP44-/- mice were activated for two days under iTreg polarizing conditions (1ug and 4ug/ml anti-CD3/CD28 cross-linking antibodies, 100 U/ml IL-2 and 5ng/ml TGF-β) or Th1-inducing conditions (IL-12 (10ng/ml), anti-IL-4 (10ug/ml). FOXP3 induction was assessed by flow cytometry. Bar graph was displayed. RT-PCR results were normalized relative to the housekeeping gene GAPDH. Shown are the results of 3 biological replicates +/- SEM.

Appendix Figure S2

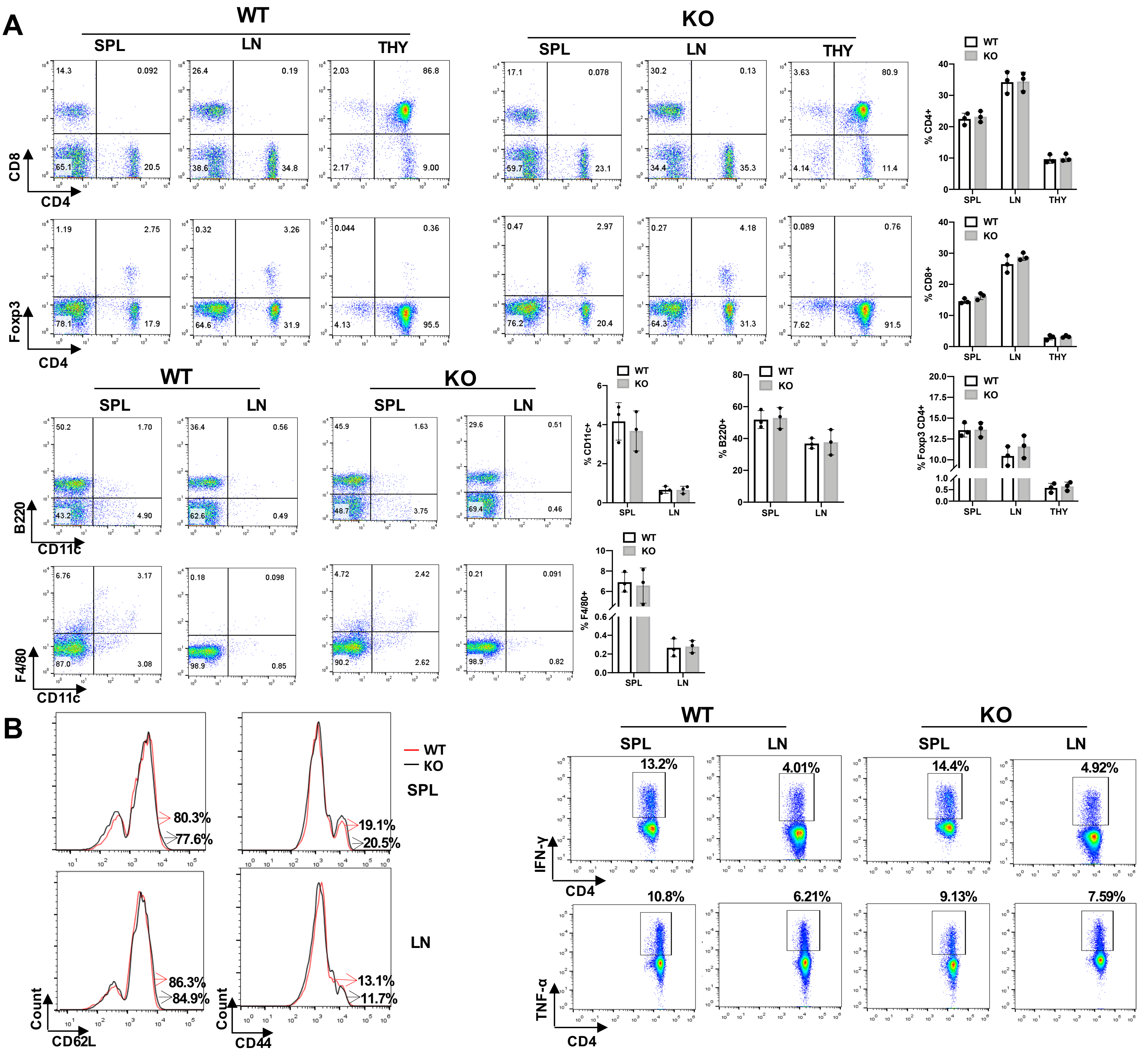

Figure S2. Analysis of the leukocyte populations of wild type and global USP44 knockout mice. (A) The lymph nodes, sple thymus of USP44 knockout mice and their wild type (WT) littermates (n= 5 per experiment; 8 weeks old) were harvested and stained for markers of T cell subsets, macrophages, B cells and Dendritic cells. Baseline frequencies of these cell types including FOXP3+ Treg in each tissue were found by flow cytometry analysis. Bar graph was displayed. Shown are the representative findings of 3 biological replicates. **(B)** Analysis the baseline of activation markers and cytokines expression in CD4+ T cells of wild type and KO mice. The lym notes and spleens of KO and age- and sex- matched wild type (WT) were harvested and stained for the marker of T cell activation (lef panel), and cytokines (TNF-α and IFN-γ) expression (right panel).

Appendix Figure S3

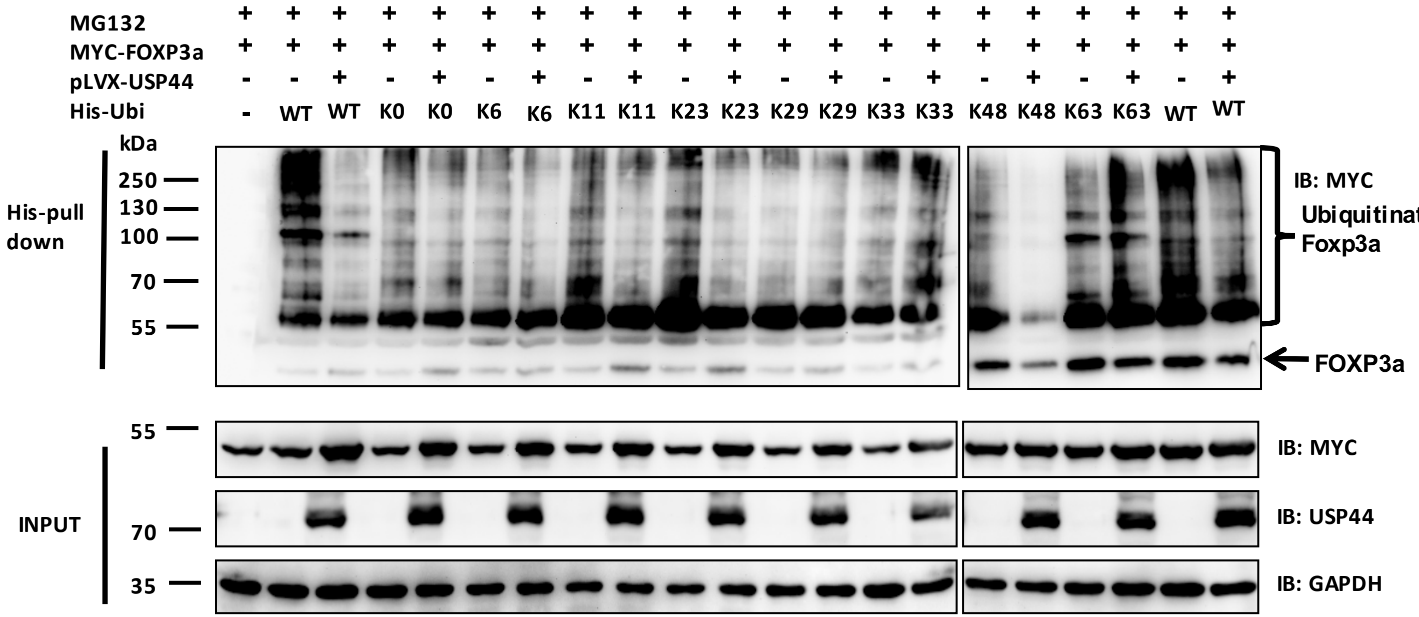

Figure S3. **USP44 downregulates K48-linked polyubiquitination of FOXP3.** HEK293T cells were transfected with plasmids encoding MYC-FOXP3, pLVX-USP44, and either wild type ubiquitin molecules or different His-tagged ubiquitin lysine mutants (each capable of participating in specific polyubiquitination linkages). The wild type status or the lone possible inter-ubiquitin linkage for each ubiquitin construct is indicated. Cells were then treated with 20 μM MG132 for 4 hrs before harvest and lysis. Pull-down of ubiquitinated proteins was accomplished using Ni-NTA beads, and ubiquitinated FOXP3 species were visualized by western blotting using antibodies specific for MYC. Shown are the results from 3 biological replicates.

Appendix Figure S4

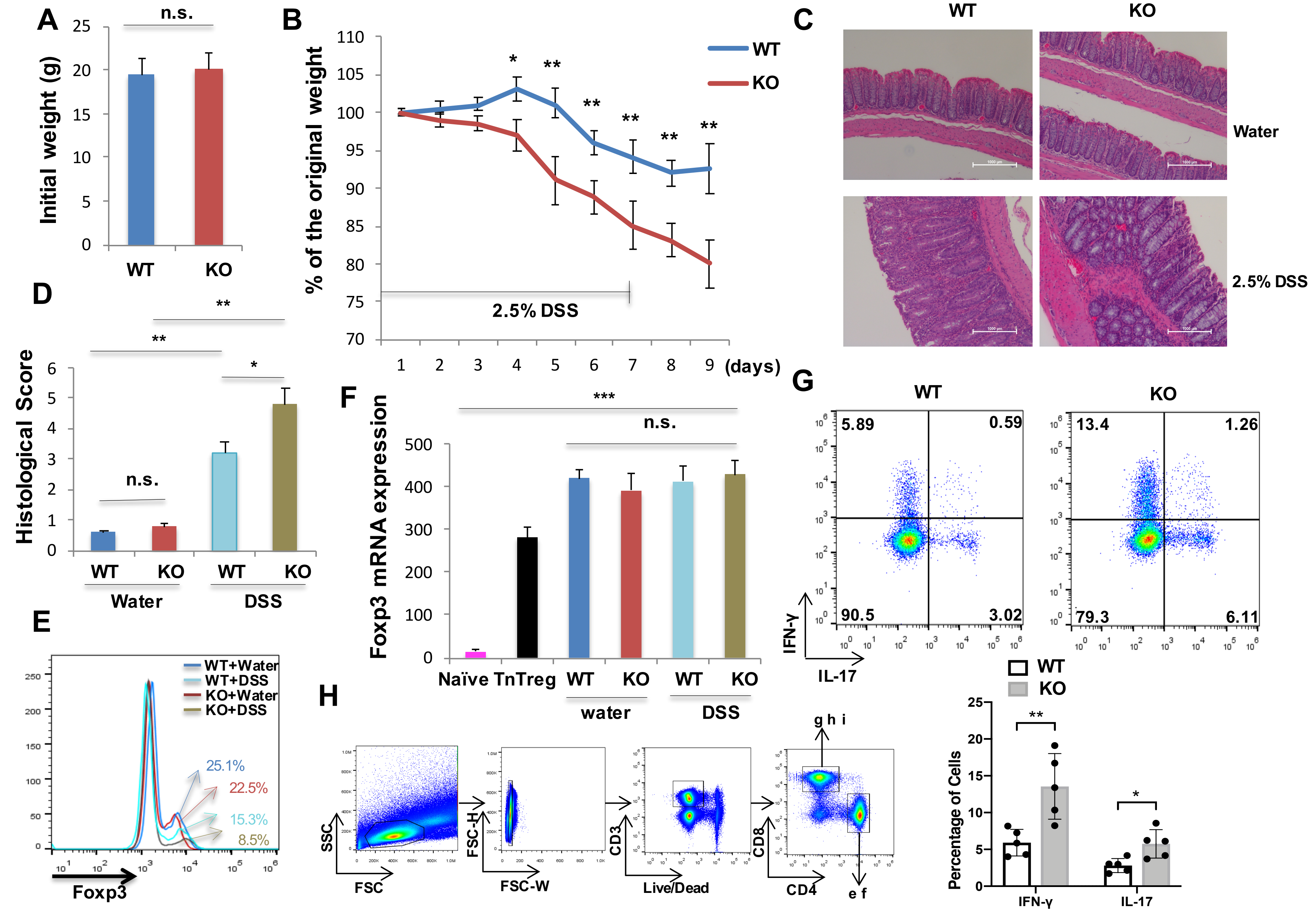

Figure S4. Deletion of USP44 in Tregs renders mice susceptible to more severe disease in the DSS-induced colitis model. (A) Prior to DSS challenge, age- and sex-matched wild type (WT, Foxp3-yfp-Cre+) and Treg-specific USP44 knockout (KO, Usp44$^{fl/fl}$Foxp3-yfp-Cre+) mice were weighted (n= 10/group/experiment). **(B)** Mice were given DSS in drinking water (2.5%) for 7 days followed by normal drinking water for an additional 2 days. Mice were weighted daily and changes relative to initial bodyweight were determined for DSS treated mice and additional control cohorts given only water (a negative control for disease; not shown). **(C, D)** At experiment's end (9 days post challenge), mice were euthanized and colon tissues were excised, fixed with 10% buffered formalin and sectioned prior to H&E staining and histopathological scoring by a blinded observed in order to further assess disease severity. Representative micrographs **(C)** and averaged histology scores **(D)** are shown. **(E)** Proportions of FOXP3+ Tregs were found among colon lamina propria-infiltrating CD4+ cells. Levels of FOXP3 protein were observed by flow cytometry after intracellular staining. **(F)** *Foxp3* transcript levels in Tregs of both groups fed normal water and DSS were found by qRT-PCR. Here (Foxp3)yfp+ Tregs from the colon leukocyte suspensions mentioned above were purified by FACS before RNA isolation, cDNA generation, and qRT-PCR analysis. Freshly isolated pools of murine nTregs (CD4+/CD25+/Foxp3-yfp+) and naïve CD4+ T cells (CD4+/CD25-/CD62L+) were included as positive and negative control groups, respectively. **(G)** The frequencies of proinflammatory cytokine-producing leukocytes in the colons of Usp44$^{fl/fl}$Foxp3-yfp-Cre+ (KO) and Foxp3-yfp-Cre+ (WT) mice were also determined by ICS. Suspensions of leukocytes recovered from the lamina propria of the indicated mice treated with DSS were re-stimulated ex vivo by a 5 hr culture in the presence of PMA, ionomycin, and brefeldin A prior to intracellular staining for IFNγ and IL-17. Bar graph was displayed. **(H)** Representative flow cytometry gating strategy for Figure 7, the cells were gated on CD4+ and CD8+ T cells. Panels **A**, **B**, **D**, and f show the mean values of 3 biological replicates +/-SEM representative of the findings from 3 biological replicates. Panels **C**, **E**, and **G** depict representative micrographs and flow cytometry results. All error bars depict the SEM.

Appendix Figure S5

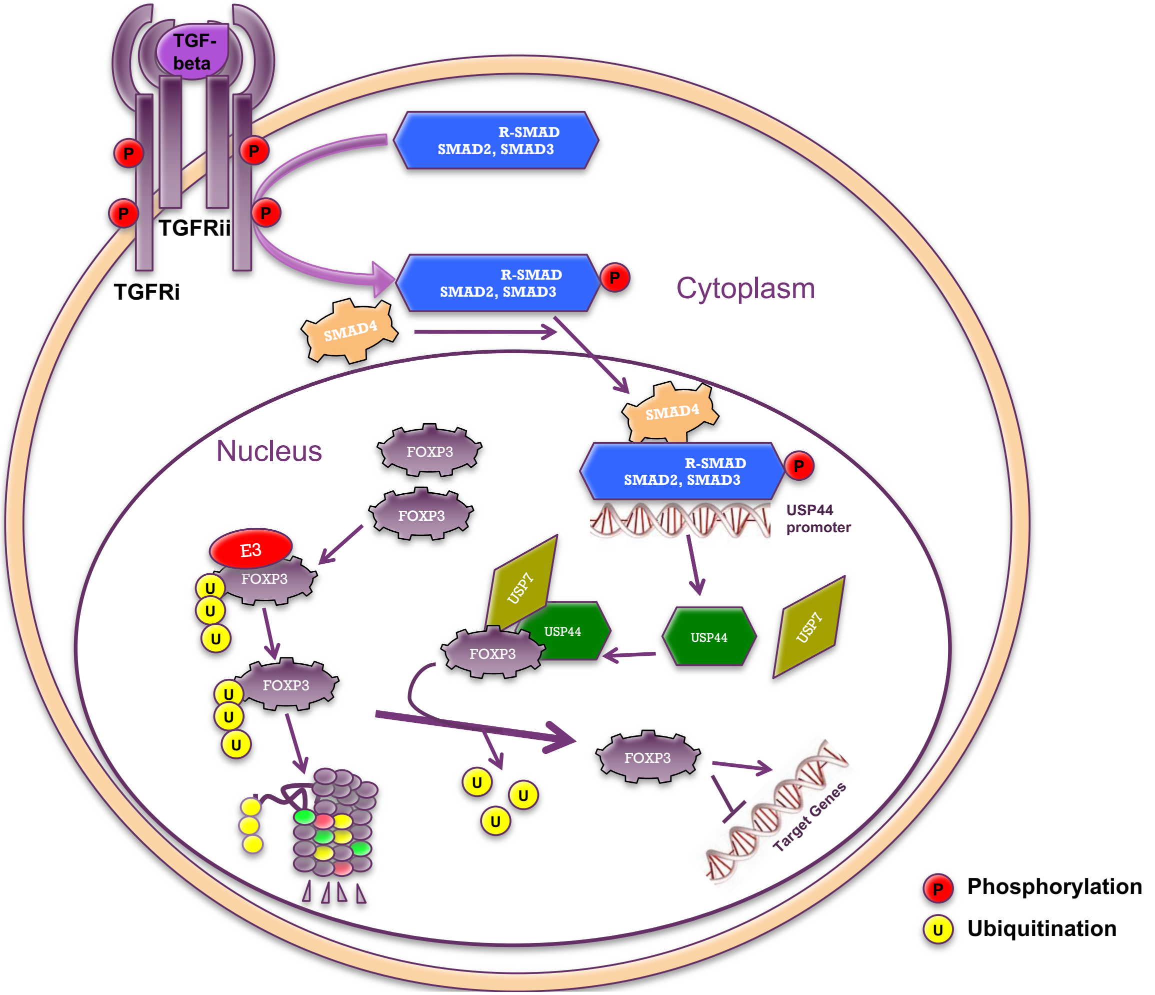

Figure S5. Summarized Model of USP44's role in Foxp3 regulation. TGF-β induces USP44 transcription by triggering SMADs binding to the USP44 promoter. USP44 interacts with FOXP3 and cooperates with USP7 to deubiquintinate the transcription factor and stabilizes its expression by counteracting specific ubiquitination modifications and preventing its proteasomal turnover.
