# Supplementary material for: The deubiquitinase USP44 promotes Treg function during inflammation by preventing FOXP3 degradation
Source: EMBO Rep. 2020 Jul 9;21(9):e50308. doi: 10.15252/embr.202050308 (PMC7507386; doi:10.15252/embr.202050308)
Supplement: Supplementary file 2 — Expanded View Figures PDF [file EMBR-21-e50308-s002.pdf]

Expanded View Figures

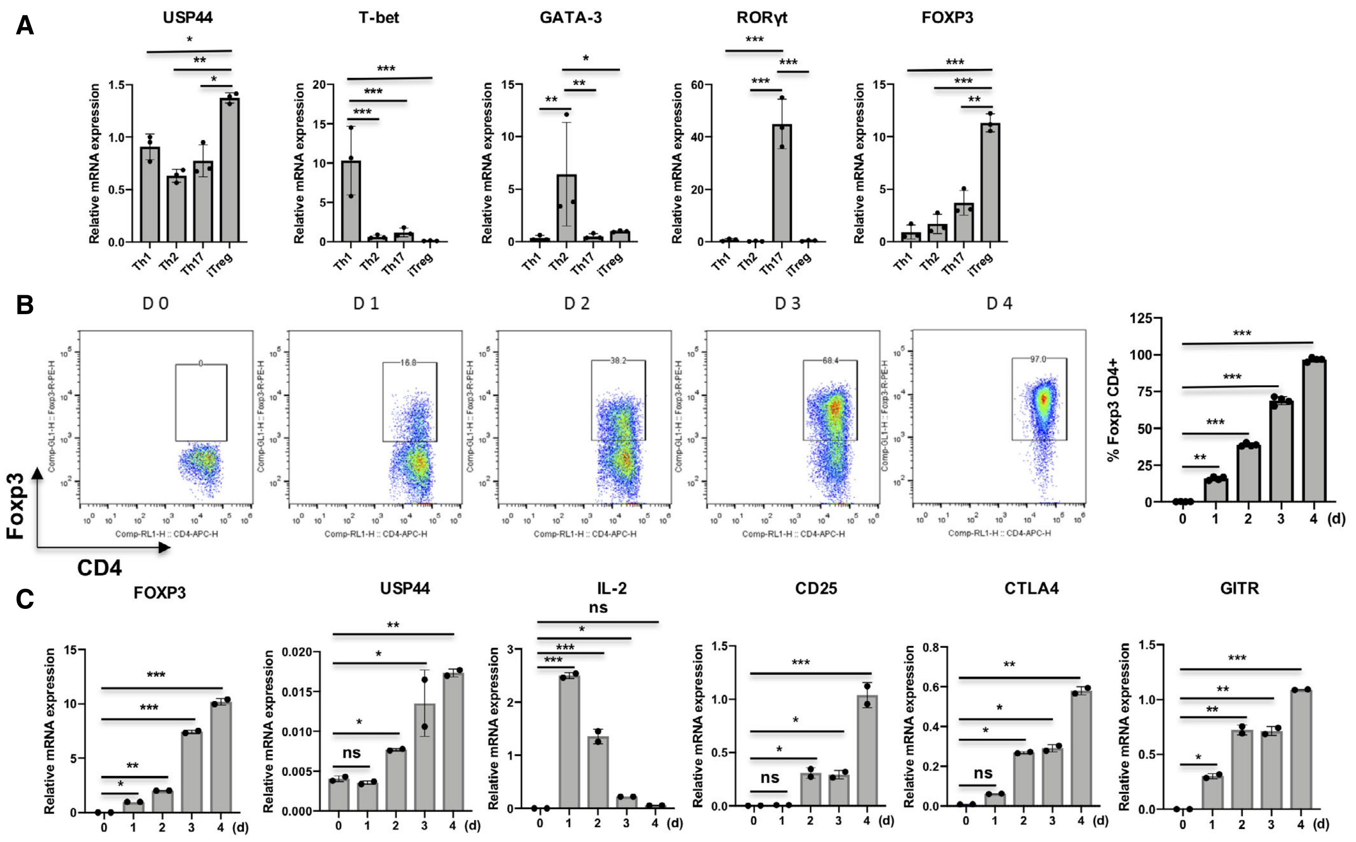

Figure EV1. USP44 is up-regulated in Tregs and largely tracked with the progressive elevation of Treg-associated transcript levels.

A Naïve CD4+ T cells were isolated from the peripheral blood of healthy donors (n = 2–3/experiment) and differentiated into the indicated T helper lineages by activation (anti-CD3/anti-CD28 antibodies; 1 and 4 μg/ml, respectively) in the presence of specific skewing reagents (described in the Materials and Methods section). Cells were harvested after 4 days, and mRNA was isolated for qRT–PCR analysis of USP44 message and that of key lineage-defining transcription factors.

B iTregs were generated by *in vitro* skewing as above, and cells were harvested at different time points. Flow cytometry confirmed the progressive up-regulation of FOXP3 under iTreg skewing conditions, and bar graph was displayed.

C qRT–PCR analysis revealed the levels of FOXP3, USP44, IL-2, CD25, CTLA-4, and GITR encoding transcripts in iTregs.

Data information: Shown in panel (B) are representative flow plots. For panels (A) and (C), mRNA expression was normalized for housekeeping gene GAPDH. Shown are mean values of biological replicates across three independent experiments ± SEM.

 © 2020 The Authors

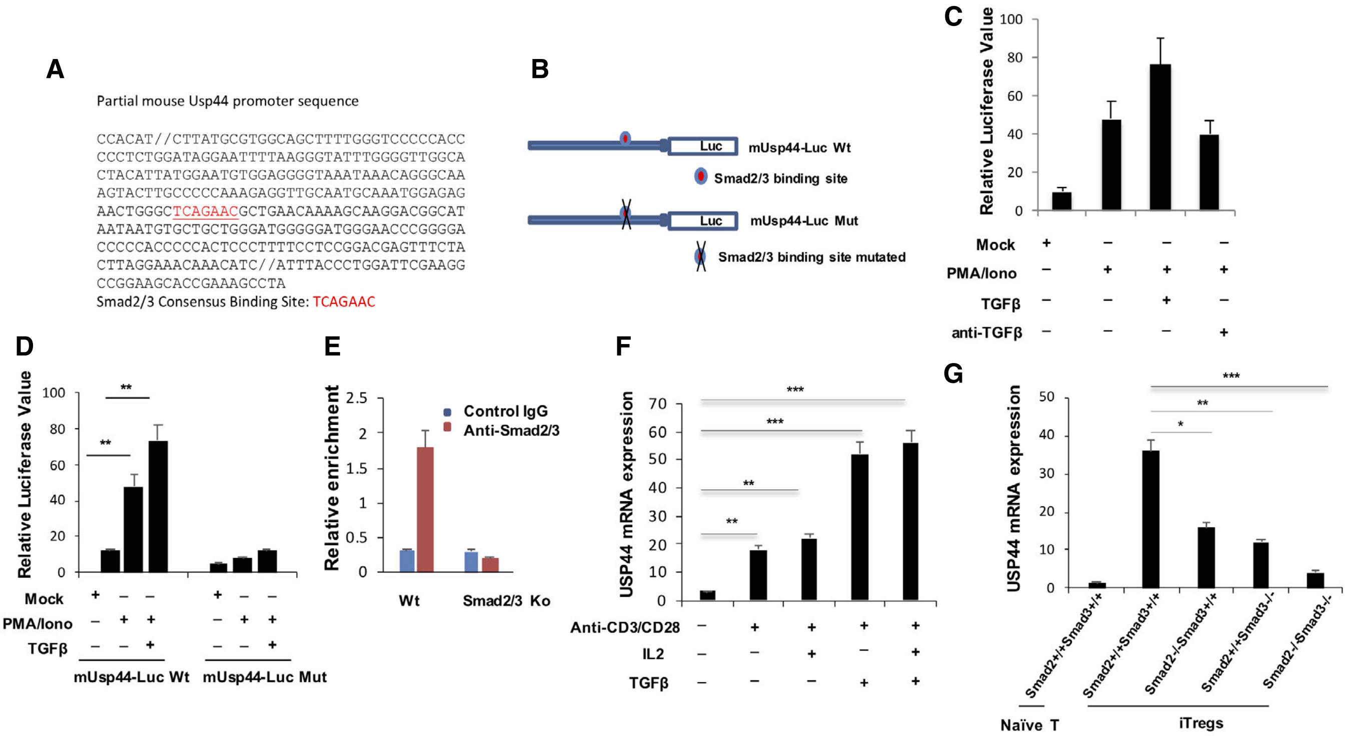

Figure EV2. USP44 expression is induced by TGFβ under Treg differentiating conditions.

A, B The USP44 promoter sequence is represented with its SMAD binding site indicated. The effect of SMAD2/3 binding site loss was found by making the mutations depicted in (B).

C Jurkat T cells were transfected with an USP44 promoter-driven firefly luciferase reporter by electroporation. Cells were stimulated with PMA and ionomycin in the presence or absence of TGF-β (2 ng/ml) or anti-TGF-β (10 μg/ml) for 8 h before being harvested for measurement of luciferase activity, which was normalized to Renilla luciferase activity.

D Jurkat T cells were transfected with an USP44 promoter-driven firefly luciferase reporter by electroporation. Cells were stimulated with PMA and ionomycin in the presence or absence of TGF-β (2 ng/ml) for 8 h before being harvested for measurement of luciferase activity, which was normalized to Renilla luciferase activity.

E ChIP analysis of SMAD occupancy of the *Usp44* promoter. Naïve T cells were isolated from C57BL/6 mice and polarized to iTreg before harvest and lysis. SMAD2 and SMAD3 factors were immunoprecipitated, and PCR was used to detect binding to the *Usp44* promoter sequence.

F FACS isolated naïve T cells were exposed to the indicated stimuli during *ex vivo* culture and were harvested at different time points for mRNA isolation and measurement of USP44 expression by qRT–PCR analysis.

G Naïve $CD4^+$ T cells were isolated from wild-type mice ($Smad2^{+/+}Smad3^{+/+}$) as well as mice lacking SMAD2 ($Smad2^{-/-}Smad3^{+/+}$), SMAD3 ($Smad2^{+/+}Smad3^{-/-}$), or both SMAD2 and SMAD3 ($Smad2/3^{-/-}$), all on a C57BL/6 background (n = 3/group/experiment). Cells were activated under *in vitro* Treg-inducing conditions (anti-CD3/CD28, 1 and 4 μg/ml, respectively) in the presence of IL-2 (100 U/ml) and TGF-β (5 ng/ml) for 4 days. Then, RNA was extracted, and cDNA was prepared in order to assess USP44 expression levels in these cells by qRT–PCR.

Data information: Panels (C–G) depict mean results from three independent experiments (biological replicates) ± SEM. *P < 0.05, **P < 0.02, ***P < 0.002; Student's t-test.

© 2020 The Authors

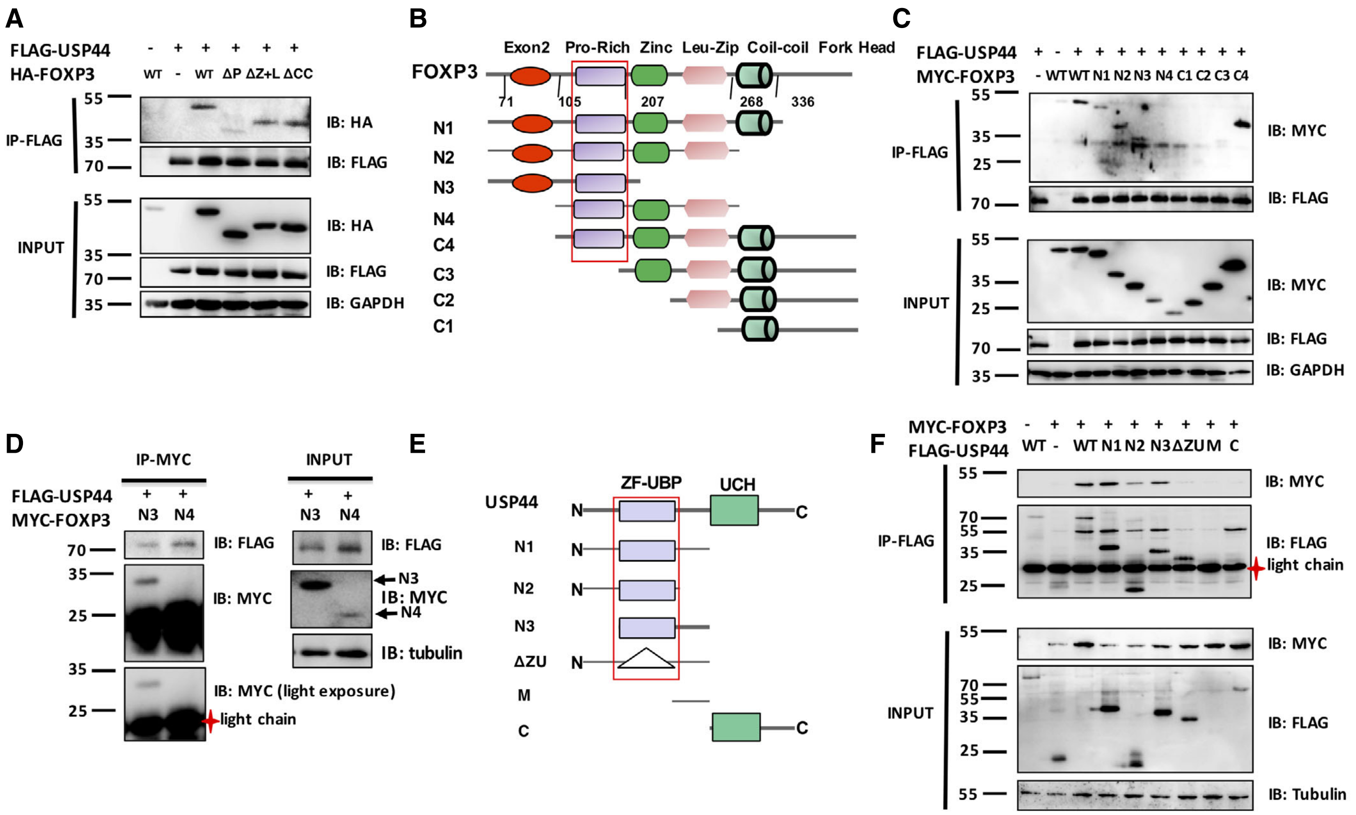

Figure EV3. The Pro-rich domain of FOXP3 is required for the association between USP44 and FOXP3 and the stabilization of FOXP3.

A Different FOXP3 deletion constructs were generated as shown and were co-transfected with or without FLAG-USP44 into HEK293T cells. The cell lysate was immunoprecipitated by anti-FLAG antibody, and FOXP3 levels were detected by Western blotting.

B, C Different truncated FOXP3 constructs were generated as shown and were co-transfected with or without FLAG-USP44 into HEK293T cells. The cell lysate was immunoprecipitated by anti-FLAG antibody, and FOXP3 levels were detected by immunoblotting for the MYC tag.

D HEK293T cells were transfected with constructs encoding MYC-FOXP3N3 or N4 truncations and FLAG-USP44. These cells were lysed and pertinent factors were immunoprecipitated using anti-MYC. Pulled-down proteins were analyzed by immunoblot using anti-FLAG and anti-MYC antibodies.

E, F Different truncated USP44 constructs were generated as shown and were transfected with or without a MYC-FOXP3 encoding plasmid into HEK293T cells. FOXP3 protein was immunoprecipitated from cell lysate by anti-MYC antibody, and USP44 levels were detected by immunoblotting. Shown are representative findings from at least three experiments.

Source data are available online for this figure.

 © 2020 The Authors

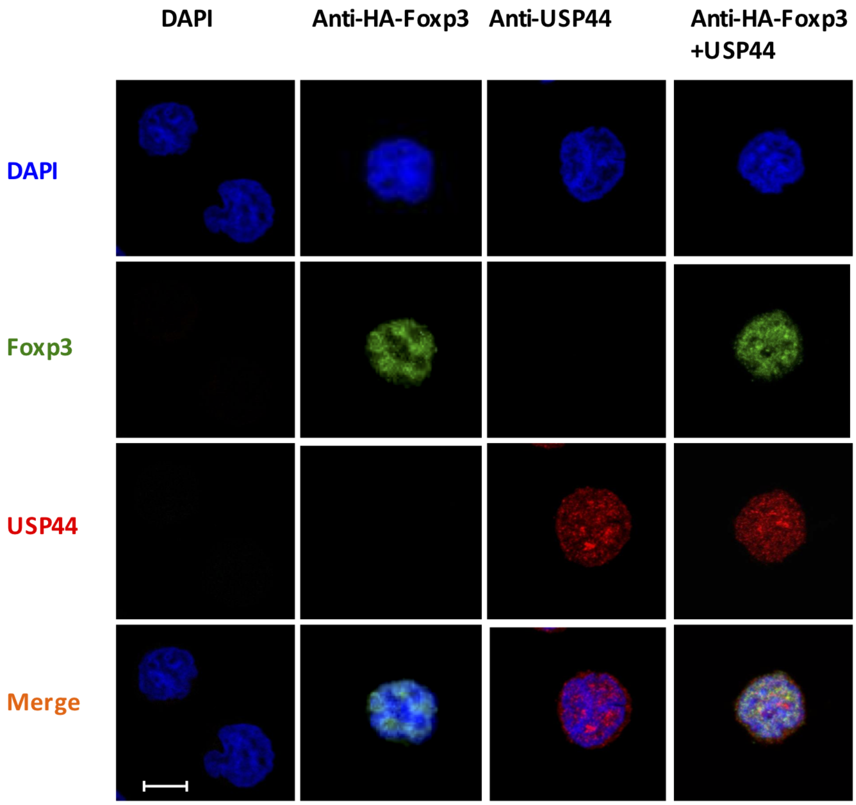

Figure EV4. Physical interaction between USP44 and FOXP3a was visualized by immunofluorescence microscopy.

Jurkat-HA-FOXP3 cells were adhered to slides coated with poly-L-lysine (1:4) for 1 h and then fixed by PFA for 0.5 h. Samples were blocked for 1 h and then incubated with the indicated specific antibodies. Representative confocal microscopy images were visualized for endogenous USP44 (red) and FOXP3. DAPI was used to visualize the nuclei (blue). Shown are the findings representative of three biological replicates; scale bar 5 µm.

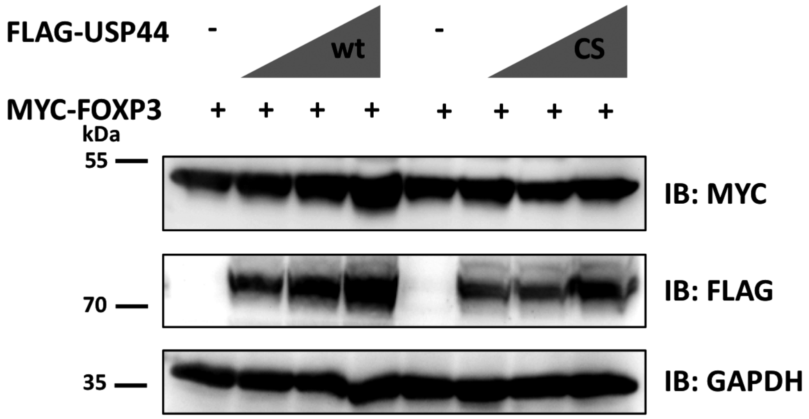

Figure EV5. USP44 stabilizes FOXP3 in a dose-dependent manner.

HEK293T cells were transfected with an expression construct encoding MYC-FOXP3 (0.5 µg), and varying levels (0.5, 1, 1.5 µg) of vectors encoding FLAG-tagged wild-type USP44 (wt) or the mutant (Kralovics *et al*, 2005), after 48 h transfected cells were harvested and lysed in RIPA buffer. Immunoblots were analyzed using anti-FLAG or anti-MYC antibodies. Shown is a representative result of three independent experiments (biological replicates).

Source data are available online for this figure.

© 2020 The Authors
