# Supplementary figures and images for: The deubiquitinase USP44 promotes Treg function during inflammation by preventing FOXP3 degradation
Source: EMBO Rep. 2020 Jul 9;21(9):e50308. doi: 10.15252/embr.202050308 (PMC7507386; doi:10.15252/embr.202050308)

# Figure EV3

## Figure EV3A

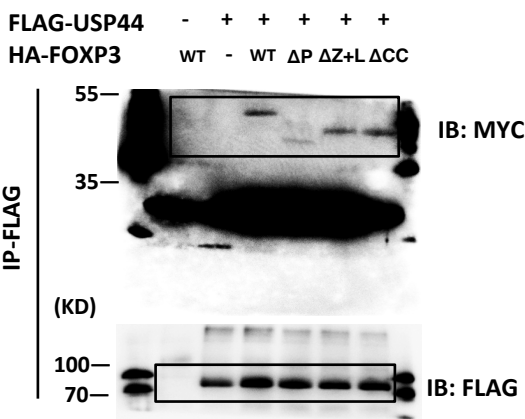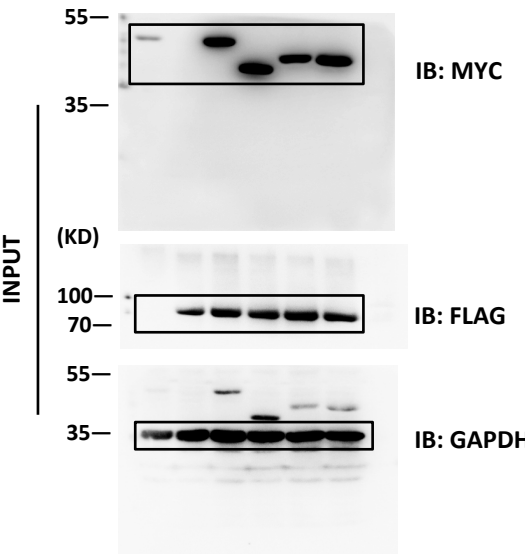

## Figure EV3C

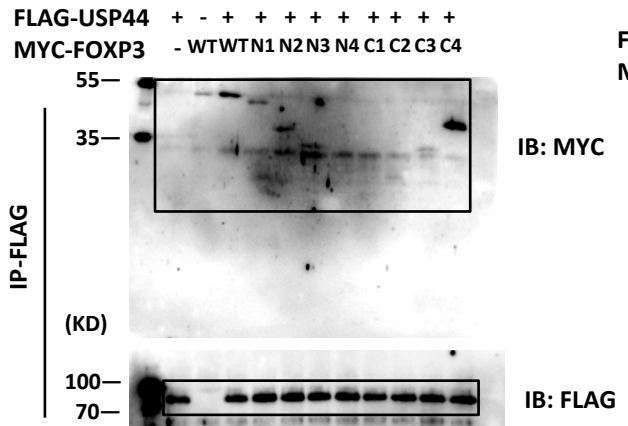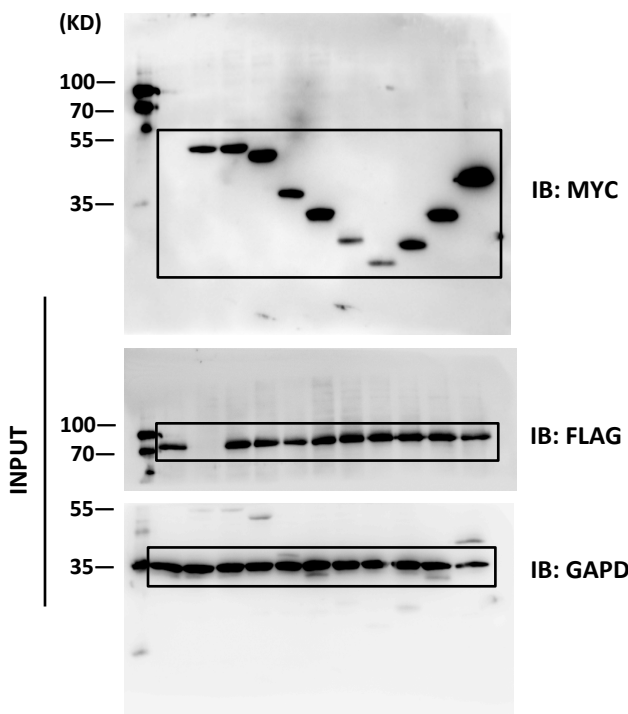

## Figure EV3D

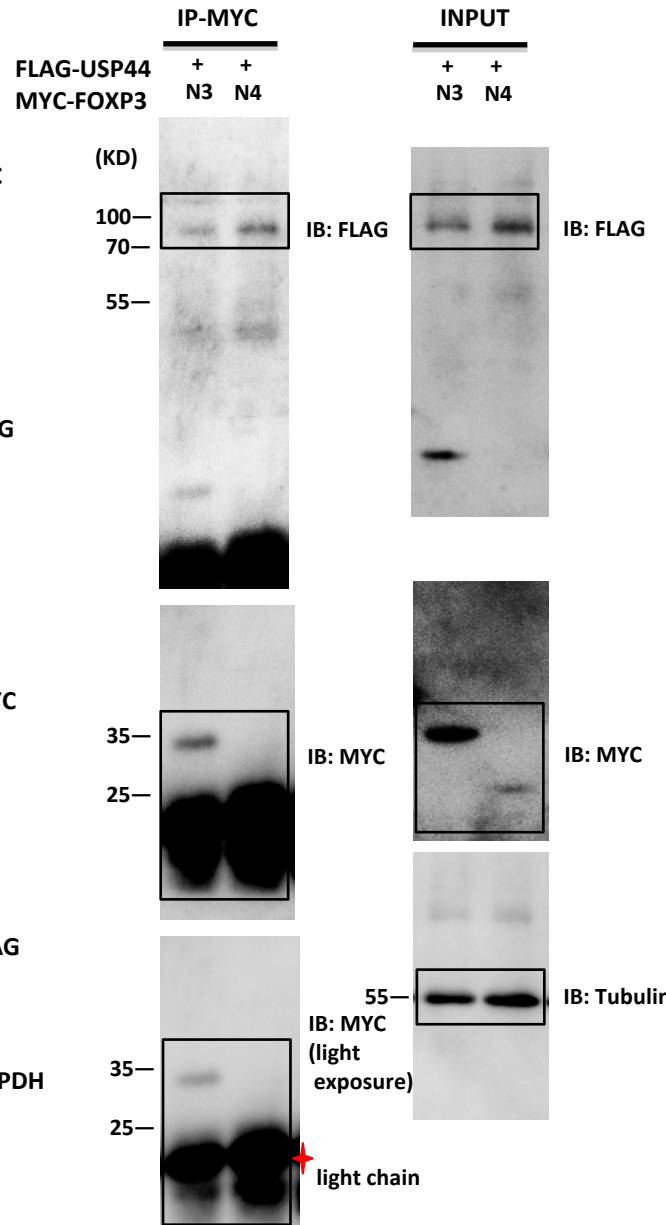

## Figure EV3F

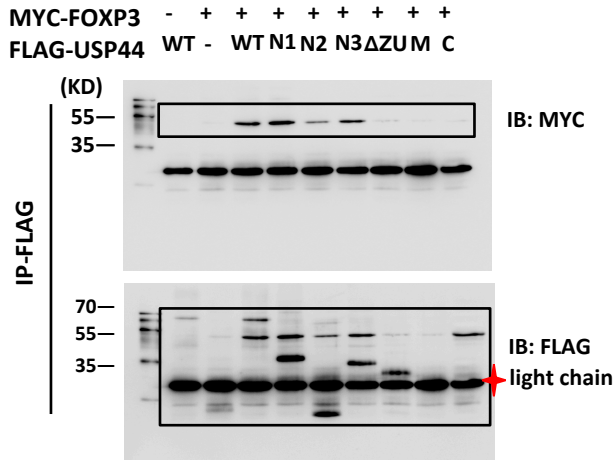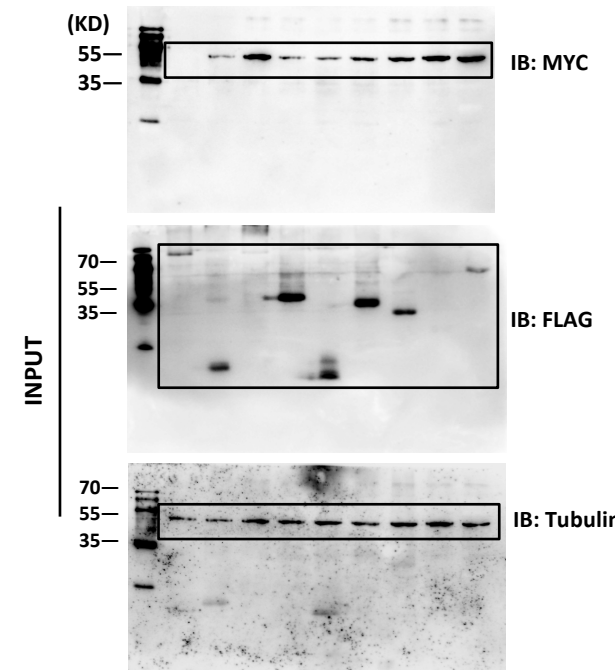

Supplement: Supplementary file 3 — Source Data for Expanded View and Appendix [file EMBR-21-e50308-s007.zip › Appendix_and_EV_Source_Data/Source_Data_FigureEV3.pdf]

Figure EV5

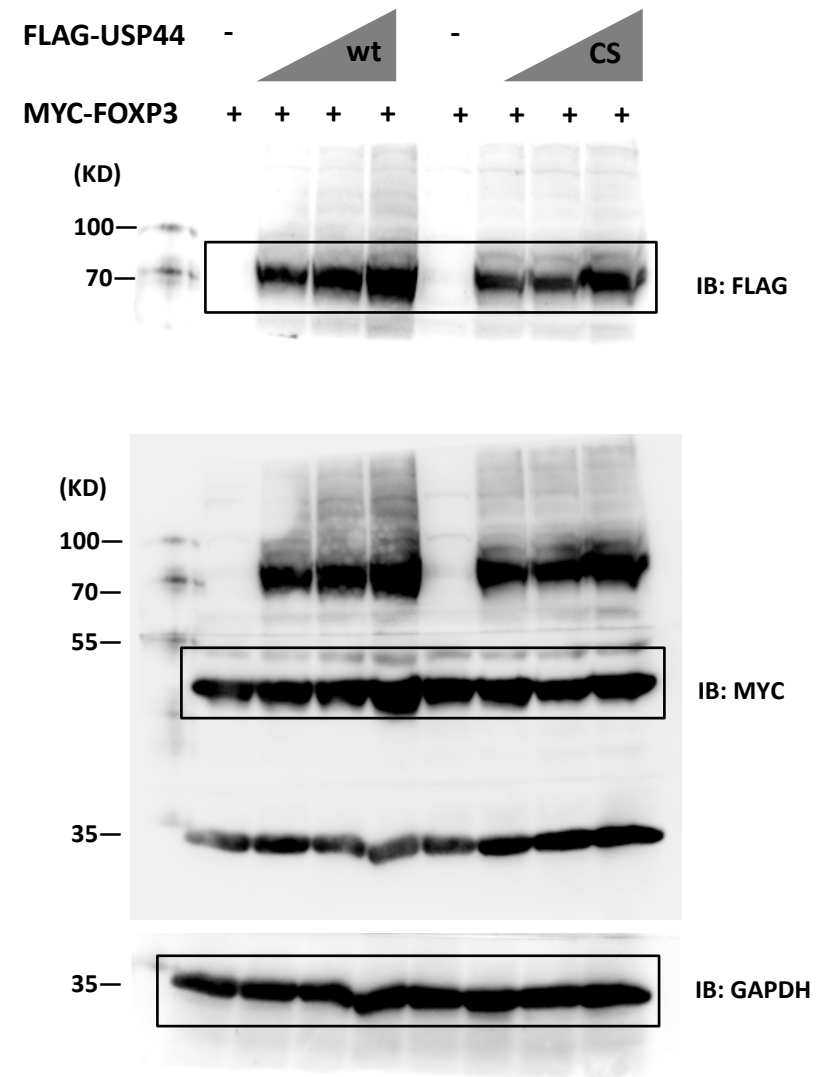

Supplement: Supplementary file 3 — Source Data for Expanded View and Appendix [file EMBR-21-e50308-s007.zip › Appendix_and_EV_Source_Data/Source_Data_FigureEV5.pdf]

# Appendix Figure S1

## Appendix Figure S1A

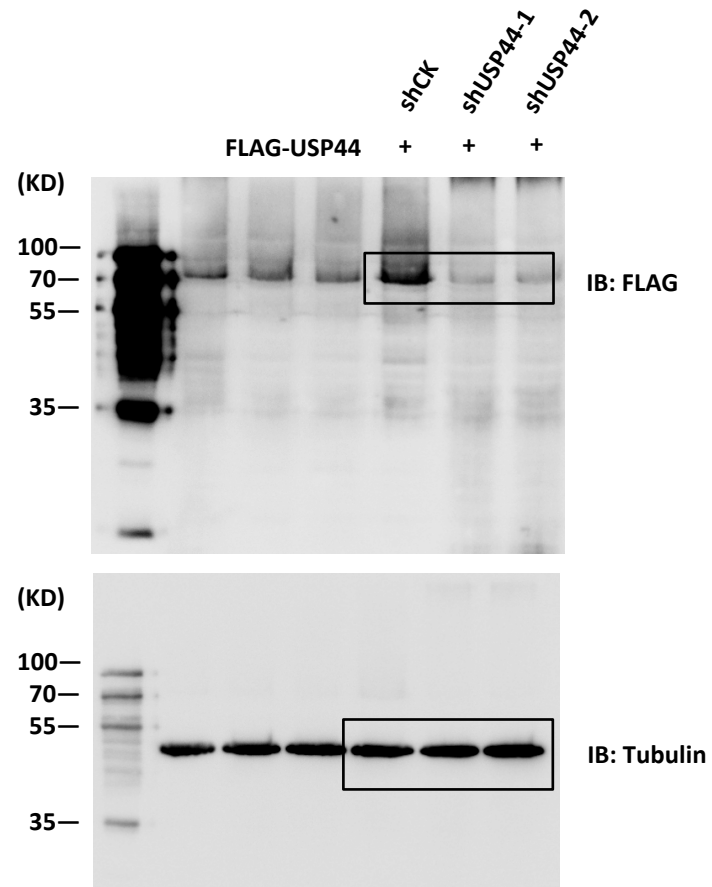

## Appendix Figure S1B

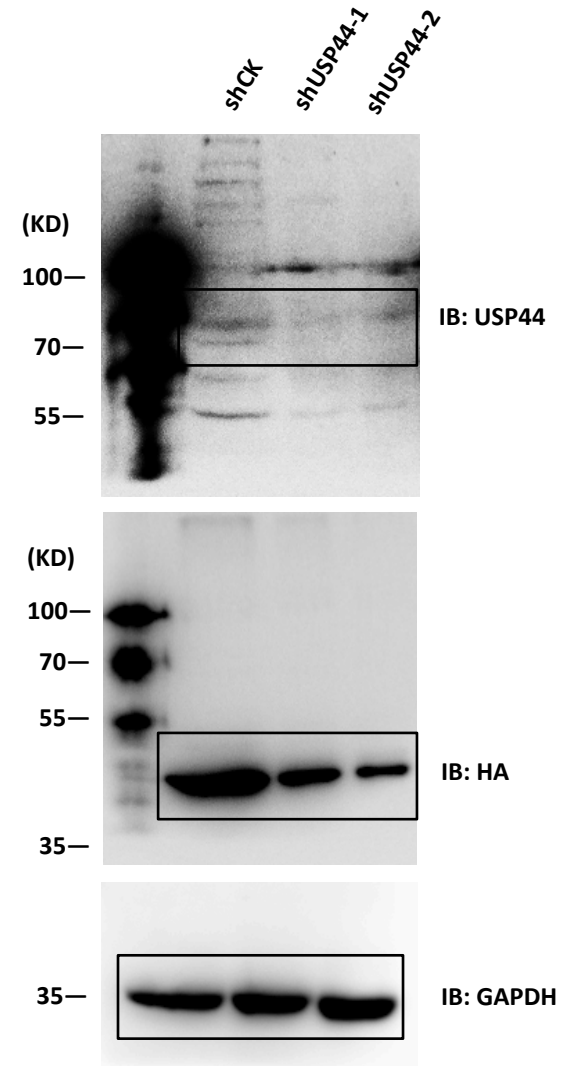

Supplement: Supplementary file 3 — Source Data for Expanded View and Appendix [file EMBR-21-e50308-s007.zip › Appendix_and_EV_Source_Data/Source_Data_Appendix_FigureS1.pdf]

Appendix Figure S3

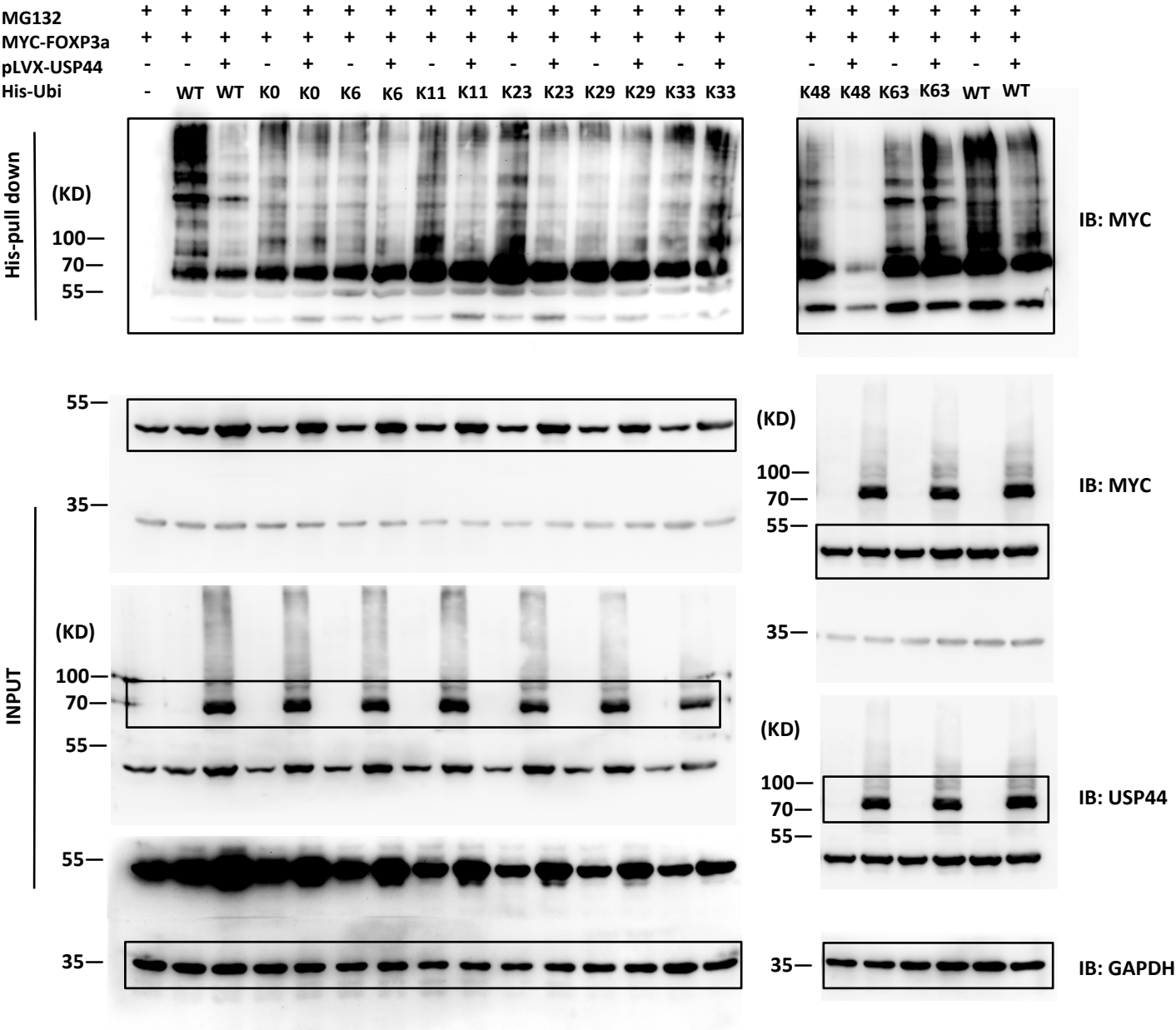

Supplement: Supplementary file 3 — Source Data for Expanded View and Appendix [file EMBR-21-e50308-s007.zip › Appendix_and_EV_Source_Data/Source_Data_Appendix_FigureS3.pdf]

# Figure 1

Figure 1A  
Bottom

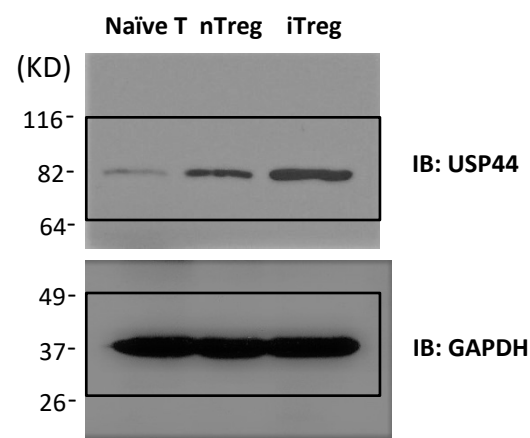

Figure 1C

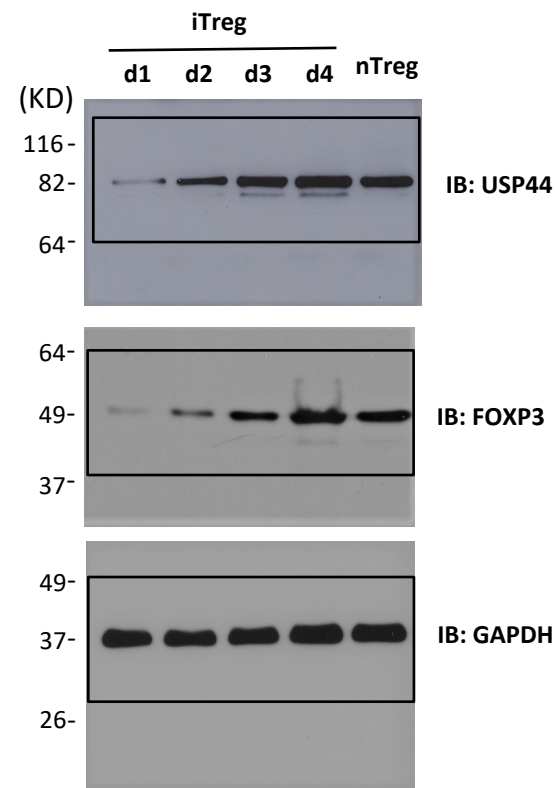

Figure 1D

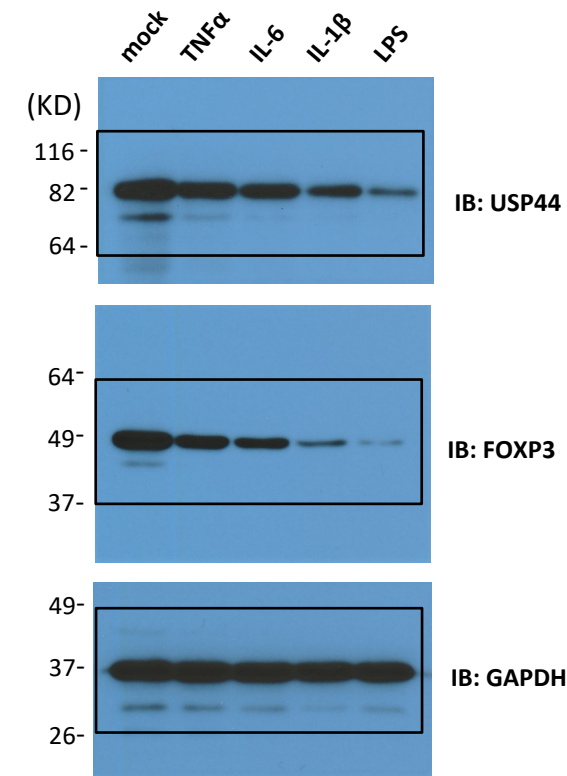

Supplement: Supplementary file 5 — Source Data for Figure 1 [file EMBR-21-e50308-s003.pdf]

Figure 2

Figure 2A left

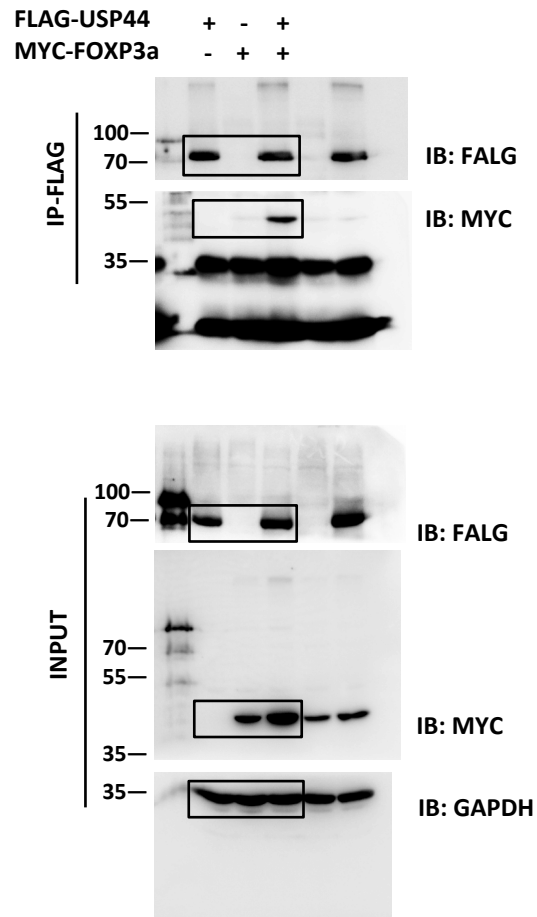

Figure 2A right

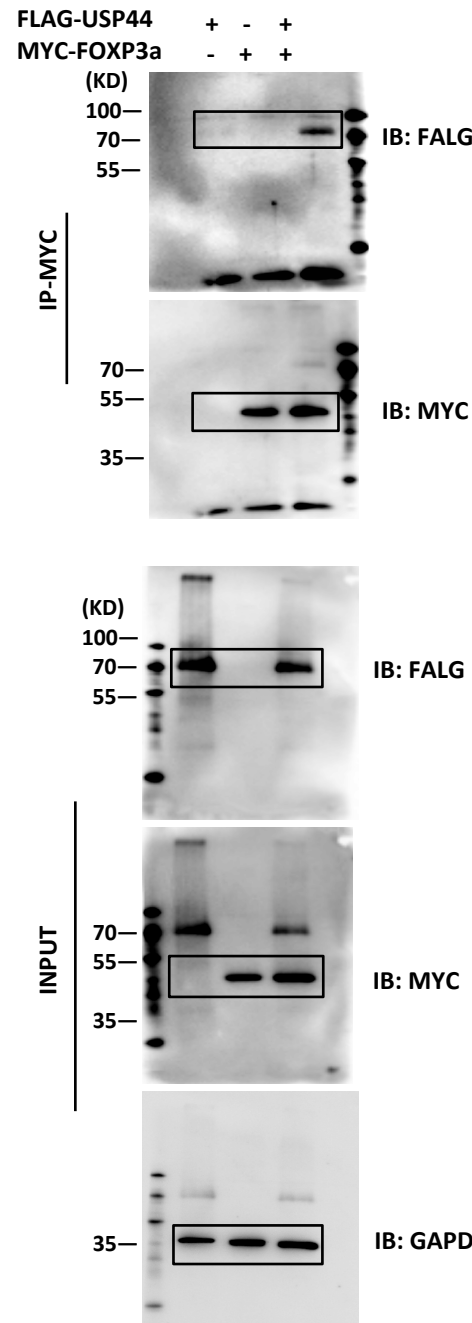

Figure 2B

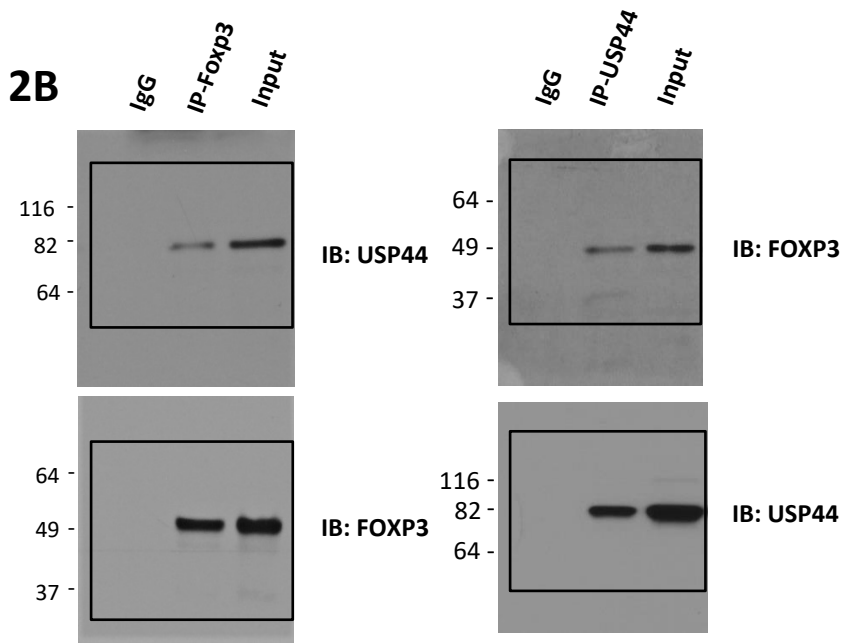

Figure 2C

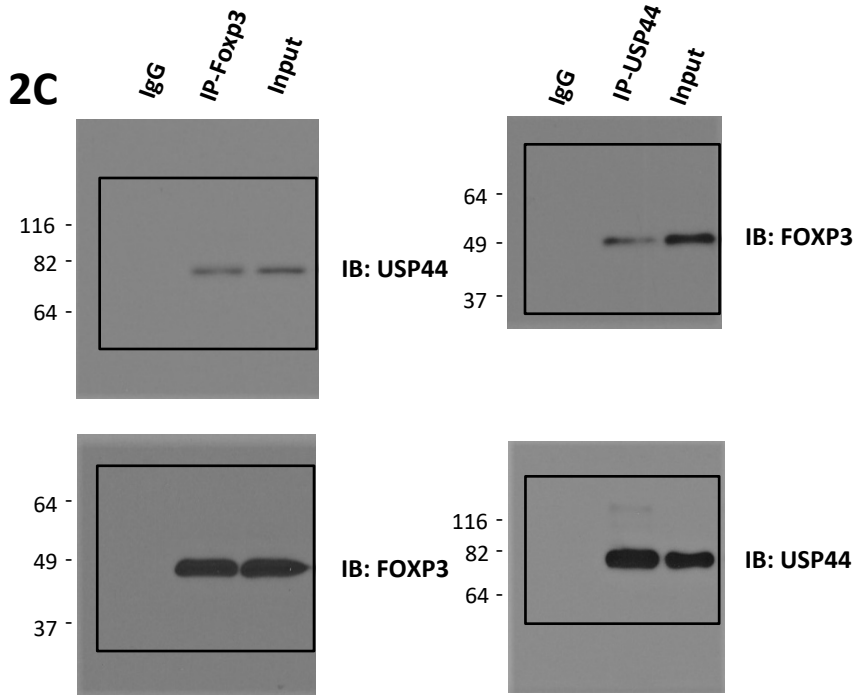

Supplement: Supplementary file 6 — Source Data for Figure 2 [file EMBR-21-e50308-s004.pdf]

Figure 3

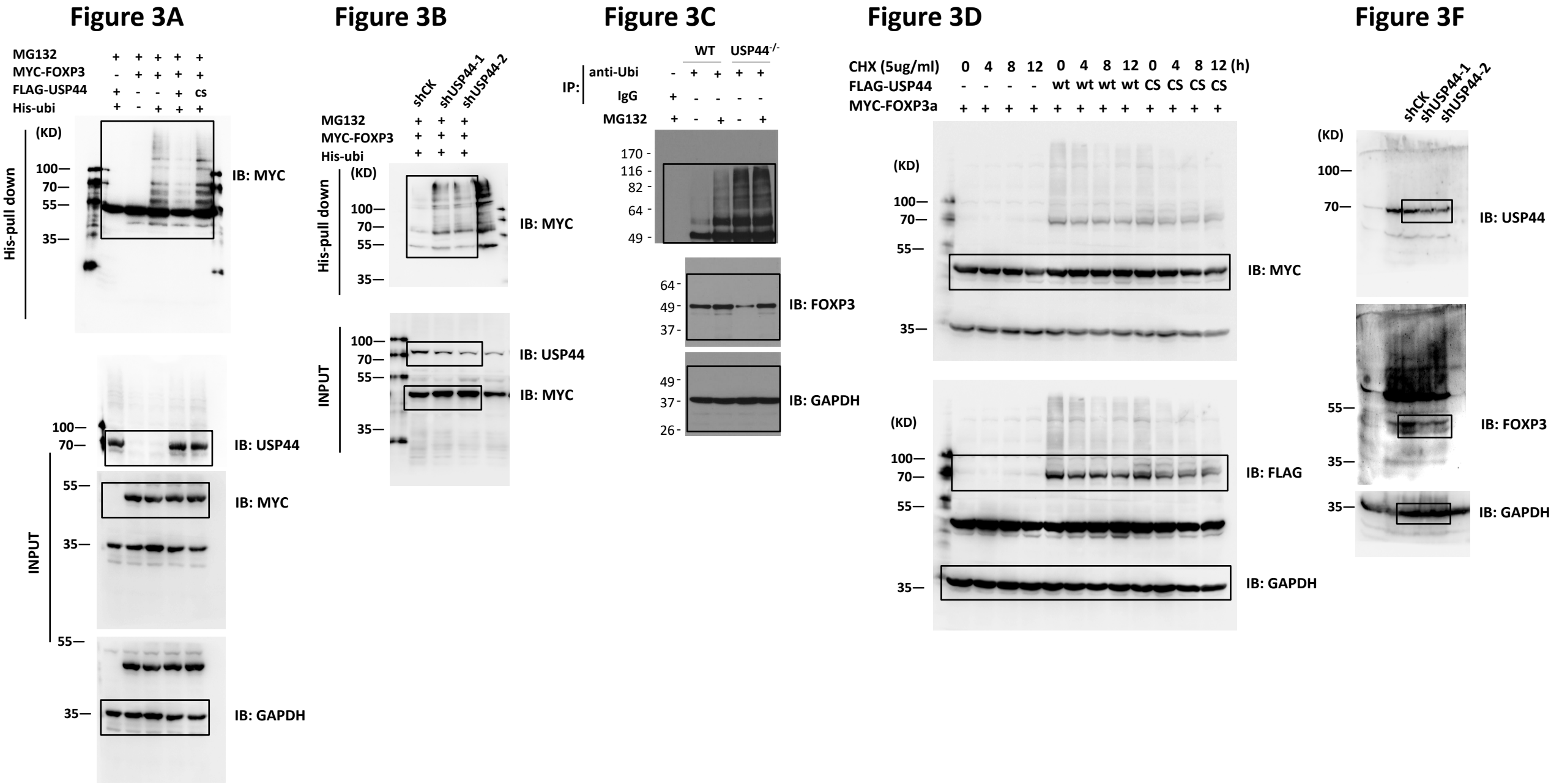

Supplement: Supplementary file 7 — Source Data for Figure 3 [file EMBR-21-e50308-s005.pdf]

Figure 4

Figure 4A left

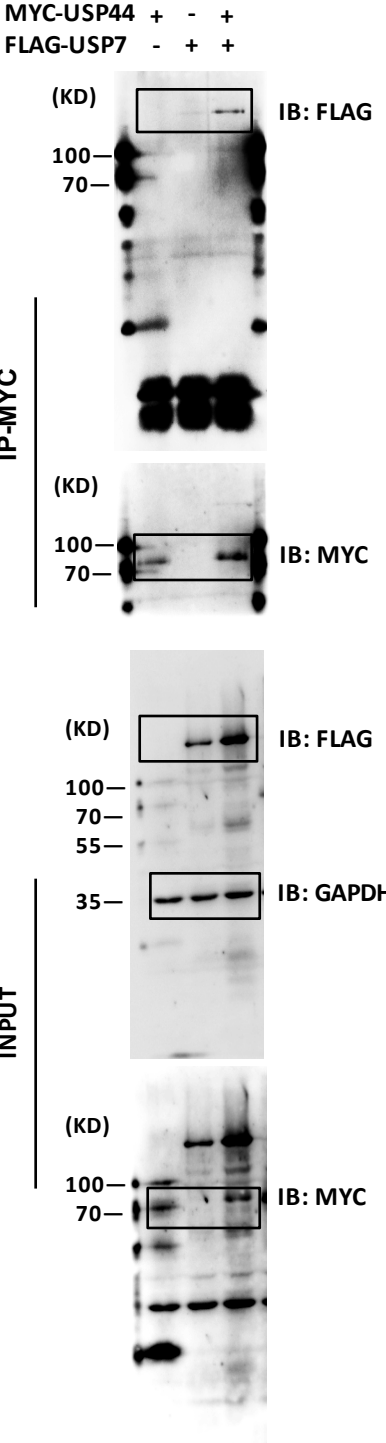

Figure 4A right

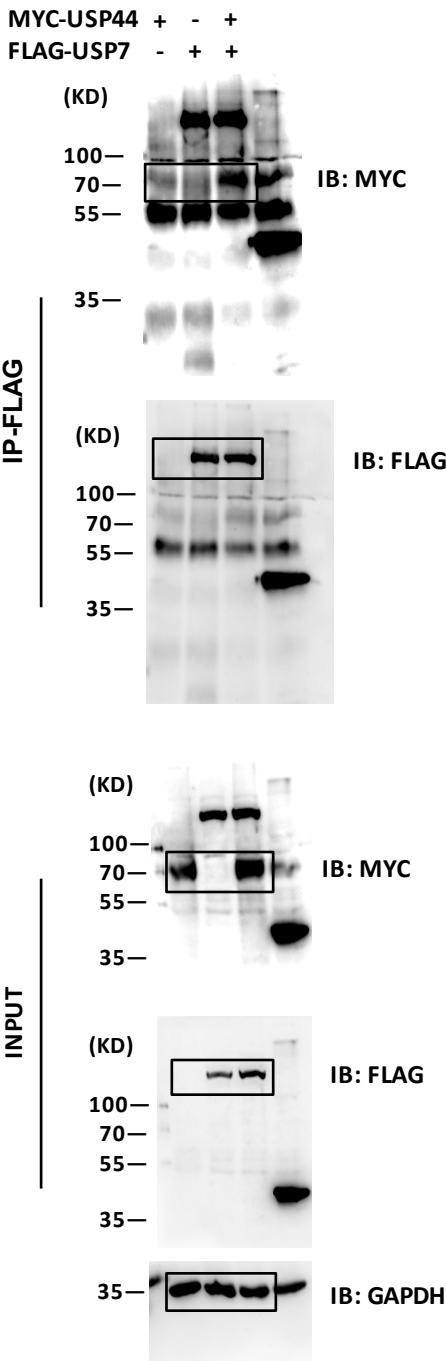

Figure 4B

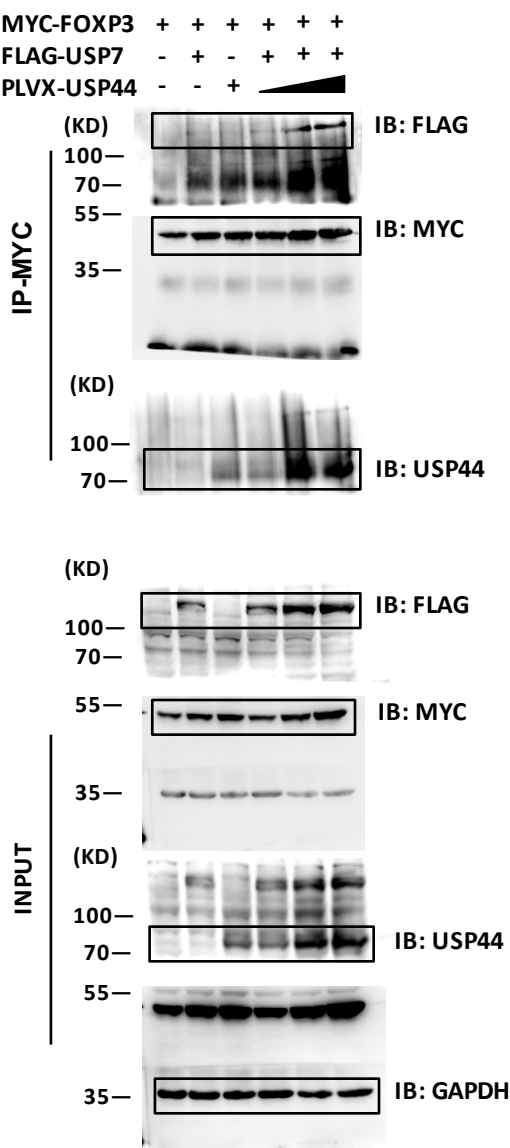

Figure 4C

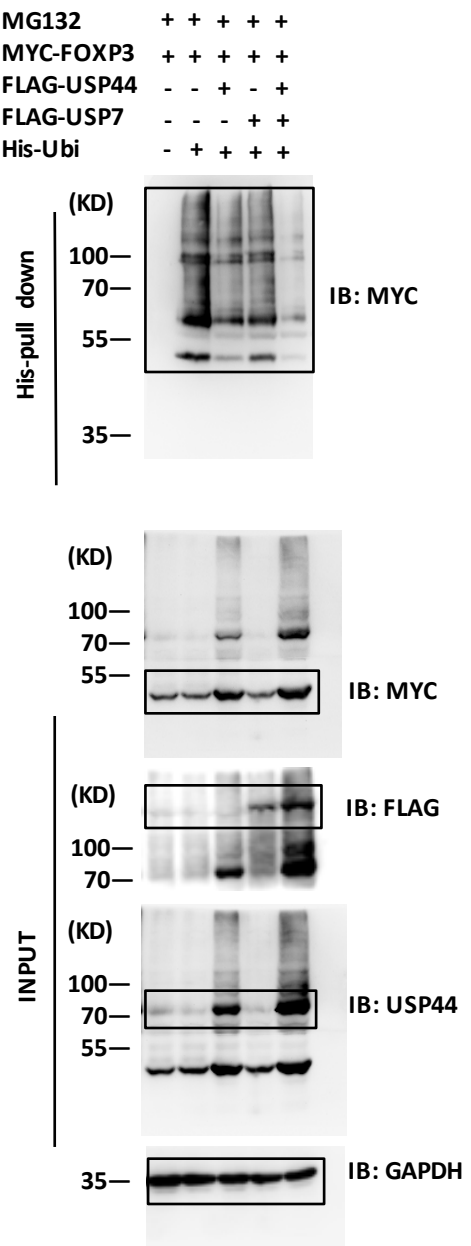

Figure 4D

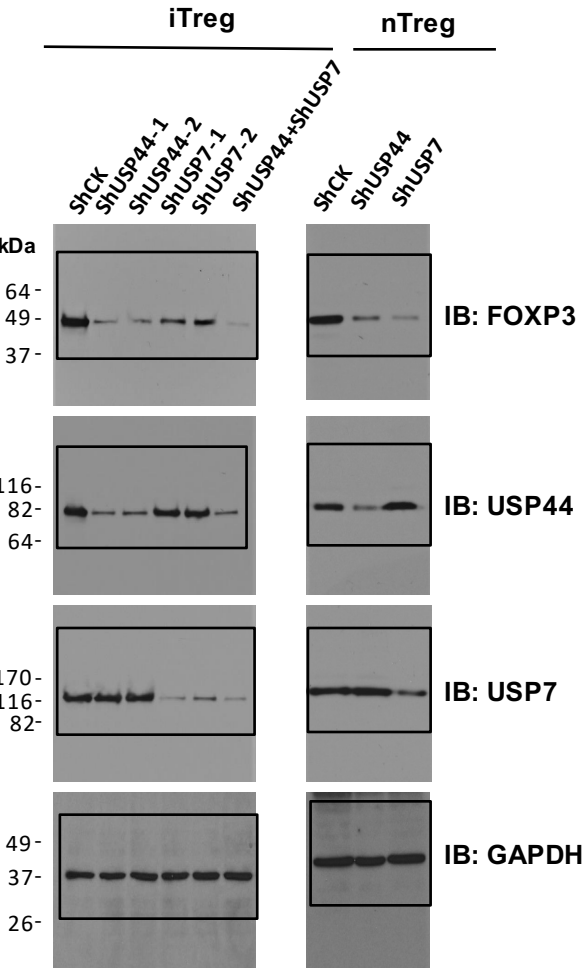

Supplement: Supplementary file 8 — Source Data for Figure 4 [file EMBR-21-e50308-s006.pdf]
