# Supplementary material for: The deubiquitinase USP44 promotes Treg function during inflammation by preventing FOXP3 degradation
Source: EMBO Rep. 2020 Jul 9;21(9):e50308. doi: 10.15252/embr.202050308 (PMC7507386; doi:10.15252/embr.202050308)
Supplement: Supplementary file 4 — Review Process File [file EMBR-21-e50308-s008.pdf]

EMBO reports

The deubiquitinase USP44 promotes Treg function during inflammation by preventing FOXP3 degradation

Bin Li, Jing Yang, Ping Wei, Joseph Barbi, Qianru Huang, Yakun Bai, Jia Nie, Yanhang Gao, Evan Yang, Jinhui Tao, Ying Lu, Chichu Xie, Xiaoxia Hou, Jiazi Ren, Xingmei Wu, Jian Meng, Ying Zhang, Juan Fu, Wei Kou, Yayi Gao, Zuojia Chen, Rui Liang, Andy Tsun, Dan Li, Wenzhi Guo, Shuijun Zhang, Song-Guo Zheng, Junqi Niu, Paul Galardy, Xuemei Tong, Guochao Shi, Huabin Li, and Fan Pan
DOI: 10.15252/embr.202050308

Corresponding author(s): Bin Li (binli@shsmu.edu.cn) , Fan Pan (fpan1@jhmi.edu), Huabin Li (allergyli@126.com)

Review Timeline:

Submission Date:	26th Feb 20
Editorial Decision:	16th Mar 20
Revision Received:	5th May 20
Editorial Decision:	28th May 20
Revision Received:	10th Jun 20
Accepted:	18th Jun 20

Editor: Achim Breiling

Transaction Report:

No Peer Review Process File is available with this article, as the authors have chosen not to make the review process public.

EMBO PRESS

YOU MUST COMPLETE ALL CELLS WITH A PINK BACKGROUND ➔

PLEASE NOTE THAT THIS CHECKLIST WILL BE PUBLISHED ALONGSIDE YOUR PAPER

Corresponding Author Name: Bin Li, Fan Pan, Huabin Li
Journal Submitted to: EMBO Reports
Manuscript Number: EMBOR-2020-50308-T

Reporting Checklist For Life Sciences Articles (Rev. June 2017)

This checklist is used to ensure good reporting standards and to improve the reproducibility of published results. These guidelines are consistent with the Principles and Guidelines for Reporting Preclinical Research issued by the NIH in 2014. Please follow the journal's authorship guidelines in preparing your manuscript.

A- Figures
1. Data
The data shown in figures should satisfy the following conditions:

- ➔ the data were obtained and processed according to the field's best practice and are presented to reflect the results of the experiments in an accurate and unbiased manner.
- ➔ figure panels include only data points, measurements or observations that can be compared to each other in a scientifically meaningful way.
- ➔ graphs include clearly labeled error bars for independent experiments and sample sizes. Unless justified, error bars should not be shown for technical replicates.
- ➔ if n< 5, the individual data points from each experiment should be plotted and any statistical test employed should be justified
- ➔ Source Data should be included to report the data underlying graphs. Please follow the guidelines set out in the author ship guidelines on Data Presentation.

2. Captions
Each figure caption should contain the following information, for each panel where they are relevant:

- ➔ a specification of the experimental system investigated (eg cell line, species name).
- ➔ the assay(s) and method(s) used to carry out the reported observations and measurements
- ➔ an explicit mention of the biological and chemical entity(ies) that are being measured.
- ➔ an explicit mention of the biological and chemical entity(ies) that are altered/varied/perturbed in a controlled manner.
- ➔ the exact sample size (n) for each experimental group/condition, given as a number, not a range;
- ➔ a description of the sample collection allowing the reader to understand whether the samples represent technical or biological replicates (including how many animals, litters, cultures, etc.).
- ➔ a statement of how many times the experiment shown was independently replicated in the laboratory.
- ➔ definitions of statistical methods and measures:
  - common tests, such as t-test (please specify whether paired vs. unpaired), simple χ2 tests, Wilcoxon and Mann-Whitney tests, can be unambiguously identified by name only, but more complex techniques should be described in the methods section;
  - are tests one-sided or two-sided?
  - are there adjustments for multiple comparisons?
  - exact statistical test results, e.g., P values = x but not P values < x;
  - definition of 'center values' as median or average;
  - definition of error bars as s.d. or s.e.m.

Any descriptions too long for the figure legend should be included in the methods section and/or with the source data.

In the pink boxes below, please ensure that the answers to the following questions are reported in the manuscript itself. Every question should be answered. If the question is not relevant to your research, please write NA (non applicable). We encourage you to include a specific subsection in the methods section for statistics, reagents, animal models and human subjects.

USEFUL LINKS FOR COMPLETING THIS FORM

http://www.antibodypedia.com	Antibodypedia
http://1degreebio.org	1DegreeBio
http://www.equator-network.org/reporting-guidelines/improving-bioscience-research-repor	ARRIVE Guidelines
http://grants.nih.gov/grants/olaw/olaw.htm	NIH Guidelines in animal use
http://www.mrc.ac.uk/Ourresearch/Ethicsresearchguidance/Useofanimals/index.htm	MRC Guidelines on animal use
http://ClinicalTrials.gov	Clinical Trial registration
http://www.consort-statement.org	CONSORT Flow Diagram
http://www.consort-statement.org/checklists/view/32-consort/66-title	CONSORT Check List
http://www.equator-network.org/reporting-guidelines/reporting-recommendations-for-tumo	REMARK Reporting Guidelines (marker prognostic studies)
http://datadryad.org	Dryad
http://figshare.com	Figshare
http://www.ncbi.nlm.nih.gov/gap	dbGAP
http://www.ebi.ac.uk/ega	EGA
http://biomodels.net/	Biomodels Database
http://biomodels.net/miriam/	MIRIAM Guidelines
http://jjj.biochem.sun.ac.za	JWS Online
http://oba.od.nih.gov/biosecurity/biosecurity_documents.html	Biosecurity Documents from NIH
http://www.selectagents.gov/	List of Select Agents

B- Statistics and general methods

Please fill out these boxes ➔ (Do not worry if you cannot see all your text once you press return)

1.a. How was the sample size chosen to ensure adequate power to detect a pre-specified effect size?	For cell lines testing, our sample sizes are similar to those reported in previously published papers, in which these sample sizes have been demonstrated to be appropriate to detect significant effects. This is reported in the Statistics section, within the methods; for animal studies, we used 1x105 cells/mouse; n=5-10 mice/group age- and sex-matched animals.
1.b. For animal studies, include a statement about sample size estimate even if no statistical methods were used.	For animal study, we used 1x105 cells/mouse; n=5-10 mice/group age- and sex-matched animals.
2. Describe inclusion/exclusion criteria if samples or animals were excluded from the analysis. Were the criteria pre-established?	No data points were excluded. All sample sizes are reported throughout the text.
3. Were any steps taken to minimize the effects of subjective bias when allocating animals/samples to treatment (e.g. randomization procedure)? If yes, please describe.	No
For animal studies, include a statement about randomization even if no randomization was used.	Mice were randomized from each group of animal models.
4.a. Were any steps taken to minimize the effects of subjective bias during group allocation or/and when assessing results (e.g. blinding of the investigator)? If yes please describe.	No
4.b. For animal studies, include a statement about blinding even if no blinding was done	For animal study, n=5-10 mice/group age- and sex-matched animals. Investigators were not blinded to the conditions of the experiment.
5. For every figure, are statistical tests justified as appropriate?	For each experimental condition, at least 3 animals were analyzed per condition. All attempts at replication were reproducible. For representative images, quantification of replicates is provided.
Do the data meet the assumptions of the tests (e.g., normal distribution)? Describe any methods used to assess it.	Tumor volume: v= (L×W2)/2.<br>Imaging analysis: Imaris v 9.2 and ImageJ<br>Flow cytometry analysis: FlowJo v7.6.1<br>Statistical analyses: Graphpad Prism Version 6, Microsoft Excel version 16.15
Is there an estimate of variation within each group of data?	NA
Is the variance similar between the groups that are being statistically compared?	NA

C- Reagents

6. To show that antibodies were profiled for use in the system under study (assay and species), provide a citation, catalog number and/or clone number, supplementary information or reference to an antibody validation profile. e.g., Antibodypedia (see link list at top right), 1DegreeBio (see link list at top right).	The antibodies used for western blotting: USP44 antibody (Abmart 1:200 and Santa Cruz 1:1000), anti-USP7 (Cell signaling 1:1000), anti-Foxy (eBioscience 1:500), anti-HA (Santa Cruz 1:1000), anti-Flag (Sigma 1:10000), anti-Myc (Santa Cruz 1:1000), anti-GAPDH (1C4, Sungene Biotech 1:5000). Confocal imaging: USP44 antibody (Abmart 1:50), anti-Foxp3 (eBioscience 1:50) anti-mouse-Dylight 488 (1:1000) and goat anti-Rabbit-Dylight 555 (1:1000), DAPI dye (1:5000). anti-HA (1:200). Flow cytometry: Fixable Viability dye eFluor 450 (BD Biosciences 1:1000), anti-CD3 (eBioscience 1:500) , anti-CD4 (eBioscience 1:400), anti-CD8 (eBioscience 1:600), anti-Foxp3 (eBioscience 1:400), anti-IFNγ (eBioscience 1:500), anti-TNFα (eBioscience 1:600), anti-IL-17(eBioscience 1:400), anti-Granzyme B (eBioscience 1:500). anti-CD25 (PC61, 1:100), anti-CD44 (IM7.8.1, 1:100), anti-CD62L (MEL14, 1:100) were from BD Biosciences, eBioscience or Miltenyl Biotec. T cell differentiation: anti-CD3 and anti-CD28 antibodies (2 and 4 ug/ml, respectively; Biolegend). anti-IFNγ (10ug/ml), anti-IL-4 (10ug/ml),anti-IL-12 (10ug/ml) all from Biolegend. The antibodies used for western blotting are widely used and were validated in the listed studies. USP44 antibody (Abmart 1:200 and Santa Cruz 1:1000), Chen, S. et al. 2016. Nucleic Acids Res. anti-USP7 (Cell signaling 1:1000),Nijman, S.M. et al. (2005) Cell 123, 773-86. Nalepa, G. et al. (2006) Nat Rev Drug Discov 5, 596-613. anti-Foxy (eBioscience 1:500), anti-HA (Santa Cruz 1:1000), anti- Flag (Sigma 1:10000), anti-Myc (Santa Cruz 1:1000), anti-GAPDH (1C4, Sungene Biotech 1:5000). Immunity. 2013 Aug 22; 39(2): 10.1016/j.immuni.2013.08.006.

	The antibodies used for flow cytometry and T cell differentiation are widely used and were validated in the listed studies. Immunity. 2013 Aug 22; 39(2): 10.1016/j.immuni.2013.08.006. The EMBO Journal (2019) 38: e99766. Nature Communications volume 7, Article number: 13559 (2016).
7. Identify the source of cell lines and report if they were recently authenticated (e.g., by STR profiling) and tested for mycoplasma contamination.	HEK 293T (ATCC CRL-11268) cells and Jurkat T cells were gift from University of Pennsylvania. Freshly isolated murine Tregs ("nTregs") were isolated from suspension of lymph node and spleen cells by FACS. Human primary cells were purified from PBMCs of healthy donors at the Shanghai Blood Center using a FACS ARIA II cell sorter (BD). MC38 colon carcinoma, B16F10 melanoma, and EL4 thymoma cell lines were purchased from ATCC. These cell lines are widely used and were validated in the listed studies. Immunity. 2013 Aug 22; 39(2): 10.1016/j.immuni.2013.08.006. The EMBO Journal (2019) 38: e99766. Nature Communications volume 7, Article number: 13559 (2016). All the cell lines tested negative for micro-plasma contamination.

* for all hyperlinks, please see the table at the top right of the document

D- Animal Models

8. Report species, strain, gender, age of animals and genetic modification status where applicable. Please detail housing and husbandry conditions and the source of animals.	The Usp44fl/fl mouse was generated by inserting Loxp sites flanking exon 1 of USP44 gene. This strain was further back crossed to C57BL/6 mouse for 8 generation to obtain the Usp44fl/fl in pure C57BL/6 background (6-8 weeks of age), which was confirmed by chromosome typing. The Foxp3yfpcre mice used in this study were purchased from Jackson Laboratory. The Usp44fl/fl and Foxp3yfpcre mice were crossed to generate mice specifically lacking USP44 in the Tregs (Usp44fl/flFoxp3Cre+) and wild type littermate controls (Usp44fl/flFoxp3Cre-). All animal experiments were performed in specific-pathogen-free, Helicobacter-free facilities in the Johns Hopkins Animal Resource Center following national, state and institutional guidelines.
9. For experiments involving live vertebrates, include a statement of compliance with ethical regulations and identify the committee(s) approving the experiments.	Animal protocols were approved by the Johns Hopkins University Institutional Animal Care and Use Committee.
10. We recommend consulting the ARRIVE guidelines (see link list at top right) (PLoS Biol. 8(6), e1000412, 2010) to ensure that other relevant aspects of animal studies are adequately reported. See author guidelines, under 'Reporting Guidelines'. See also: NIH (see link list at top right) and MRC (see link list at top right) recommendations. Please confirm compliance.	The ARRIVE guidelines is attahed, too.

E- Human Subjects

11. Identify the committee(s) approving the study protocol.	HEK 293T (ATCC CRL-11268) cells and Jurkat T cells were gift from University of Pennsylvania. Human primary cells were purified from PBMCs of healthy donors at the Shanghai Blood Center using a FACS ARIA II cell sorter (BD).
12. Include a statement confirming that informed consent was obtained from all subjects and that the experiments conformed to the principles set out in the WMA Declaration of Helsinki and the Department of Health and Human Services Belmont Report.	Population characteristics: Human blood donors for experiments were anonymous. Recruitment: Primary human cells for all experiments were sources from healthy donors or freshly drawn whole blood under a protocol approved by the Shanghai Jiao Tong University Review Board. Ethics oversight: The informed consent for all human experiments were approved by the Institutional Review Boards of Shanghai Jiao Tong University and Institut Pasteur of Shanghai. Our studies were performed in accordance with the Declaration of Helsinki.
13. For publication of patient photos, include a statement confirming that consent to publish was obtained.	NA
14. Report any restrictions on the availability (and/or on the use) of human data or samples.	NA
15. Report the clinical trial registration number (at ClinicalTrials.gov or equivalent), where applicable.	NA
16. For phase II and III randomized controlled trials, please refer to the CONSORT flow diagram (see link list at top right) and submit the CONSORT checklist (see link list at top right) with your submission. See author guidelines, under 'Reporting Guidelines'. Please confirm you have submitted this list.	NA
17. For tumor marker prognostic studies, we recommend that you follow the REMARK reporting guidelines (see link list at top right). See author guidelines, under 'Reporting Guidelines'. Please confirm you have followed these guidelines.	NA

F- Data Accessibility

18: Provide a "Data Availability" section at the end of the Materials & Methods, listing the accession codes for data generated in this study and deposited in a public database (e.g. RNA-Seq data: Gene Expression Omnibus GSE39462, Proteomics data: PRIDE PXD000208 etc.) Please refer to our author guidelines for 'Data Deposition'.<br><br>Data deposition in a public repository is mandatory for:<br>a. Protein, DNA and RNA sequences<br>b. Macromolecular structures<br>c. Crystallographic data for small molecules<br>d. Functional genomics data<br>e. Proteomics and molecular interactions	All data generated or analyzed during this study are included in this published article and its supplementary information files. The datasets used and/or analyzed during the current study are available from the corresponding author on reasonable request.
19. Deposition is strongly recommended for any datasets that are central and integral to the study; please consider the journal's data policy. If no structured public repository exists for a given data type, we encourage the provision of datasets in the manuscript as a Supplementary Document (see author guidelines under 'Expanded View' or in unstructured repositories such as Dryad (see link list at top right) or Figshare (see link list at top right).	Source data is attahced as supplementary document.
20. Access to human clinical and genomic datasets should be provided with as few restrictions as possible while respecting ethical obligations to the patients and relevant medical and legal issues. If practically possible and compatible with the individual consent agreement used in the study, such data should be deposited in one of the major public access-controlled repositories such as dbGAP (see link list at top right) or EGA (see link list at top right).	NA
21. Computational models that are central and integral to a study should be shared without restrictions and provided in a machine-readable form. The relevant accession numbers or links should be provided. When possible, standardized format (SBML, CellML) should be used instead of scripts (e.g. MATLAB). Authors are strongly encouraged to follow the MIRIAM guidelines (see link list at top right) and deposit their model in a public database such as Biomodels (see link list at top right) or JWS Online (see link list at top right). If computer source code is provided with the paper, it should be deposited in a public repository or included in supplementary information.	NA

G- Dual use research of concern

22. Could your study fall under dual use research restrictions? Please check biosecurity documents (see link list at top right) and list of select agents and toxins (APHIS/CDC) (see link list at top right). According to our biosecurity guidelines, provide a statement only if it could.	NA
